# 4-(Aryl)-Benzo[4,5]imidazo[1,2-*a*]pyrimidine-3-Carbonitrile-Based Fluorophores: Povarov Reaction-Based Synthesis, Photophysical Studies, and DFT Calculations

**DOI:** 10.3390/molecules27228029

**Published:** 2022-11-19

**Authors:** Victor V. Fedotov, Maria I. Valieva, Olga S. Taniya, Semen V. Aminov, Mikhail A. Kharitonov, Alexander S. Novikov, Dmitry S. Kopchuk, Pavel A. Slepukhin, Grigory V. Zyryanov, Evgeny N. Ulomsky, Vladimir L. Rusinov, Valery N. Charushin

**Affiliations:** 1 Chemical Engineering Institute, Ural Federal University, 19 Mira St., 620002 Yekaterinburg, Russia; 2 Institute of Chemistry, Saint Petersburg State University, 7/9 Universitetskaya Nab., 199034 Saint Petersburg, Russia

**Keywords:** pyrimidine, benzimidazole, aza-Diels–Alder reaction, Povarov reaction, oxidation, fluorescence, aggregation-induced emission, mechanochromic properties

## Abstract

A series of novel 4-(aryl)-benzo[4,5]imidazo[1,2-*a*]pyrimidine-3-carbonitriles were obtained through the Povarov (aza-Diels–Alder) and oxidation reactions, starting from benzimidazole-2-arylimines. Based on the literature data and X-ray diffraction analysis, it was discovered that during the Povarov reaction, [1,3] sigmatropic rearrangement leading to dihydrobenzimidazo[1,2-*a*]pyrimidines took place. The structures of all the obtained compounds were confirmed based on the data from ^1^H- and ^13^C-NMR spectroscopy, IR spectroscopy, and elemental analysis. For all the obtained compounds, their photophysical properties were studied. In all the cases, a positive emission solvatochromism with Stokes shifts from 120 to 180 nm was recorded. Aggregation-Induced Emission (AIE) has been illustrated for compound **6c** using different water fractions (fw) in THF. The compounds **6c** and **6f** demonstrated changes in emission maxima or/and intensities after mechanical stimulation.

## 1. Introduction

Azolopyrimidines are ubiquitous heterocyclic systems, particularly important in living organisms as a core of purine bases, and these heterocycles are widely present among biologically active compounds, including those with antiviral [1,2,3,4], anticancer [5,6,7], antibacterial [8,9], and antidiabetic activity [10,11]. In addition to a wide range of biological activities, azolopyrimidines are considered promising candidates for important fluorescence applications [12,13,14,15]. Furthermore, strongly electron-withdrawing pyrimidine derivatives have found applications for the synthesis of push-pull molecules and the construction of functionalized π-conjugated materials such as dye-sensitized solar cells [16], non-doped OLED and laser dyes [17], and nonlinear optical materials [18]. Among the methods for the structural modification of azolopyrimidines, the approaches based on the creation of polycyclic fused analogs of azolopyrimidines such as benzo[4,5]imidazo[1,2-*a*]pyrimidines are of growing interest and significance [19,20,21]. Since polycyclic fused systems with a conjugated planar structure exhibit relevant photophysical properties, they have found applications as phosphors in optoelectronics or as fluorescent dyes for textile and polymer materials [22].

Among the methods for constructing heterocyclic systems is the aza-Diels–Alder [4 + 2] cycloaddition reaction between various dienophiles and N-aryl-substituted imines, which yields a wide range of azaheterocycles. This reaction, also known as the Povarov reaction [23,24,25,26], is a convenient tool for the construction of six-membered rings with high molecular complexity via the direct construction of carbon–carbon and carbon–heteroatom bonds [27]. In addition, the Povarov reaction is considered an important and efficient approach for creating large libraries of bioactive compounds in drug discovery programs [28]. From this point of view, the use of such a powerful synthetic methodology can be useful for the creation of new derivatives of azolopyrimidines, in particular benzo[4,5]imidazo[1,2-*a*]pyrimidines.

The use of molecules with aggregation-induced emission (AIE) properties, including those with reversible mechanochromism properties, is of great research interest due to their potential applications in biomedical imaging, sensors, and organic light-emitting diodes [29]. Additionally, fluorophores based on acceptor azaheteroarene domains, such as triazoles, oxadiazoles, thiadiazoles, benzothiazoles, quinoxalines, s- or as-triazines, and pyrimidines, are of particular interest [30,31,32,33,34]. Apart from these acceptors, imidazole-based units have been reported as electron acceptors for blue emission acquisition due to their low LUMO energy level [35]. However, the imidazole unit has been less studied for the development of efficient fluorescent materials due to its weak electron-accepting ability [36,37]. Wang et al. reported the synthesis of TPE-substituted phenanthroimidazole derivatives [38]. These compounds exhibited AIE properties as well as an intriguing mechanofluorochromism: after a short-time grinding, the blue emitting in a solid-state fluorophores (with maxima around 438 nm) changed their emission color to sky blue with a maxima near 450 nm. The functionalization of the imidazole-containing domain with a strongly electron-withdrawing cyano-group and a reduced singlet-triplet energy gap, on the other hand, has received special attention as a universal and appealing strategy for creating AIE-active fluorophores, including those with thermally activated delayed fluorescence (TADF) [39]. For instance, the authors of [40] recently developed TADF materials with C3-functionalized cyano-group 2-phenylimidazopyrazine as an acceptor unit linked to either acridine or phenoxazine donor units, and for these fluorophores an EQE of about 12.7% was achieved. In addition, the use of 2-phenylimidazo[1,2-*a*]pyridine containing cyano-group as an acceptor has been reported as a tool for designing dark blue emitters with a relatively high fluorescence quantum yield [36,41].

**Scheme 1:**
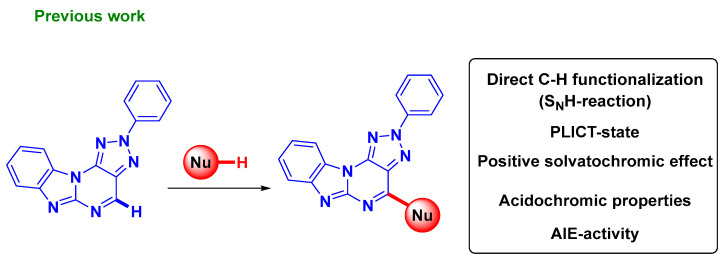
Nucleophilic substitution of hydrogen (S_N_H) in 2-phenyl-2*H*-benzo[4,5]imidazo[1,2-*a*][1,2,3]triazolo[4,5-*e*]pyrimidine [42].

We recently reported the synthesis of asymmetric donor-acceptor azoloazine fluorophors based on 4-heteroaryl-substituted 2-phenyl-2*H*-benzo[4,5]imidazo[1,2-*a*][1,2,3]triazolo[4,5-*e*]pyrimidine via the reaction of nucleophilic aromatic hydrogen substitution (S_N_H) and studied their microenvironmental sensitivity in the PLICT process (Scheme 1) [42].

Herein, we wish to report a synthetic design of novel benzo[4,5]imidazo[1,2-*a*]pyrimidines bearing cyano-group (instead of a 1,2,3-triazole fragment) via the combination of the Povarov reaction and oxidative aromatization of the resulting dihydro derivatives, as well as studies of their aggregation-induced fluorescence behavior and mechanofluorochromic properties, as well as structure-property correlation studies involving DFT methods.

## 2. Results

### 2.1. Synthesis

**Scheme 2:**
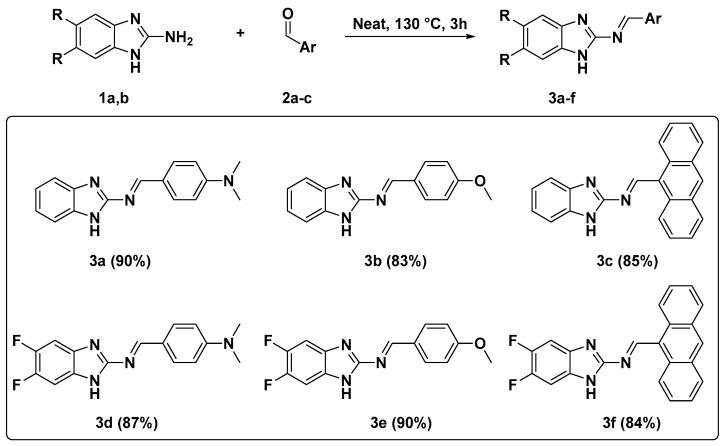
Scope of benzimidazole-2-arylimine **3a**–**f**.

Arylimines (the diene component) and various dienophiles are the classical substrates used for the Povarov reaction (the aza-Diels–Alder reaction). For the preparation of arylimines, Brønsted acid catalysis [43,44,45] and Lewis acid catalysis [46,47] are traditionally used, as are various modifications, including those involving microwave radiation [48,49,50]. Within the frame of current research, we have proposed a new catalyst-free and solvent-free method for obtaining benzimidazole-2-arylimine **3a**–**f** by heating 2-aminobenzimidazoles **1a**,**b** and aromatic aldehydes **2a**–**c** at 130 °C for 3 h. This method afforded desired diene substrates **3a**–**f** in good to excellent yields (83–90%) (Scheme 2).

The structure of all intermediates **3a**–**f** was confirmed by means of the data from ^1^H NMR spectroscopy, as well as ^13^C NMR spectroscopy, IR spectroscopy, and elemental analysis. These data were also considered for the identification of previously undescribed benzimidazole-2-arylimines **3a**, **3c**–**f** (Figures S4–S8 and S21–S23, Supplementary Materials).

**Scheme 3:**
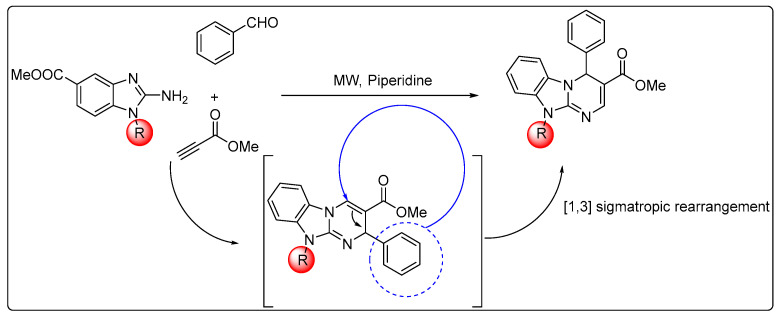
Povarov reaction and rearrangement [49].

It is worth mentioning that Chen et al. previously reported an unprecedented in situ [1,3] sigmatropic rearrangement that resulted in 4,10-dihydropyrimido[1,2-*a*]benzimidazoles [49]. Additionally, the same rearrangement was observed by us in the case of using *N*-2-substituted benzimidazoles (Scheme 3).

Inspired by this fact, we decided to investigate the possibility of rearrangement in the case of unsubstituted benzimidazole-2-arylimine. To test this possibility, derivatives **3a**–**f** were used as diene substrates in the Povarov reaction, and 3-morpholinoacrylonitrile **4** was chosen as an EWG-dienophile (Table 1). A careful literature survey revealed that the most commonly used catalysts for this type of reaction are Brønsted acids [51,52] and Lewis acids [27,53]. However, there are examples of using basic catalysts [49] as well as electrochemical methods [23]. To optimize the synthetic procedure for the reaction between benzimidazole-2-arylimine **3a** and 3-morpholinoacrylonitrile **4**, leading to the target, 4-(4-(dimethylamino)phenyl)-1,4-dihydrobenzo[4,5]imidazo[1,2-*a*]pyrimidine-3-carbonitrile **5a** was chosen. Next, the influence of the nature of the solvents and activating agents, their amounts, as well as the reaction time, on the yields of the target product was assessed (Table 1). The obtained results clearly demonstrated that BF_3_∙Et_2_O was the best activating agent when used at an amount of 1.5 equivalents in *n*-BuOH for 5 h (Table 1, entry 9).

**Scheme 4:**
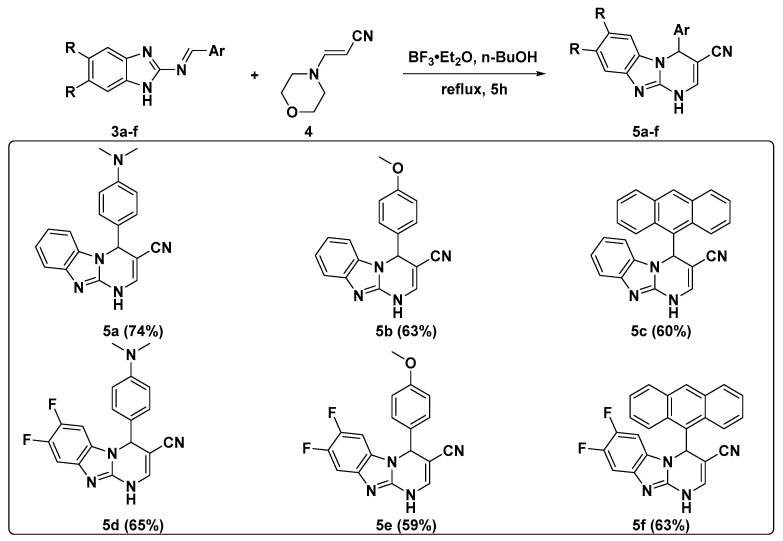
Substrate scope of dihydropyrimidines **5a**–**f**.

As a next step, by using the optimized reaction conditions, we have prepared a series of annulated dihydropyrimidines **5a**–**f** in moderate to good yields (59–74%) (Scheme 4).

The structures of the obtained dihydropyrimidines **5a**–**f** were confirmed by means of IR-, ^1^H-, and ^13^C-NMR spectroscopy as well as elemental analysis data. Due to the very low solubility of derivatives **5a**–**f** a mixture of CDCl_3_−CF_3_COOD (*v/v* = 10:1) was used as a solvent for NMR measurements. All the prepared compounds provided satisfactory analytical data. The signals H-4 are the characteristic ones for the products **5a**–**f** in the corresponding ^1^H NMR spectra. It should be noted that in compounds **5a**,**b** and **5d**,**e** the H-4 signals are located at *δ* 6.16–6.42 ppm, whereas for the derivatives **5c** and **5f**, bearing an anthracene fragment, the H-4 proton shifts downfield to the region of *δ* 7.60–7.63 ppm. Apparently, it occurs due to the deshielding effect of the H-4 proton because of the presence of the anthracene substituent. In the IR spectra, for all the series of dihydropyrimidines **5a**–**f** the characteristic stretching vibrations of (-C≡N) bonds are observed at ν 2202–2215 cm^−^^1^ (see Supplementary Materials).

The Povarov reaction is a versatile and efficient method to access the tetrahydroquinoline scaffolds [26], and, as a rule, the research on this reaction is limited only by the availability of such systems. At the same time, the oxidative aromatization products of the Povarov reaction may be of interest from the point of view of studying their properties, in particular their photophysical ones. Therefore, as a next step, the aromatization of these novel dihydropyrimidine systems **5a**–**f** was carried out.

By using compound **5a** as a key heterocyclic substrate, the most suitable solvent for the oxidation reaction was selected (Table 2). Thus, DMF seems to be the most suitable solvent for the reaction since substrate **5a** has good solubility in this solvent. Moreover, the boiling point of DMF makes it possible to carry out the reaction at high temperatures. As a first step, the blank experiments without oxidation agents (Table 2 entries 1–4) were carried out. It was found that heating the substrate **5a** in DMF resulted in the formation of the oxidation product **6a** in some amounts (according to TLC data), possibly, due to the oxidation in the ambient air. However, even after the prolonged heating (12 h) at the evaluated 140 °C temperature, the complete conversion of compound **5a** to the target product **6a** was not observed. The use of mild oxidizing agents at 120 °C, such as MnO2, reduced the reaction time to 6 h (Table 2 entries 5-8). Subsequently, the increase in the amount of MnO_2_ to four equivalents resulted in the complete conversion of **5a** within 1 h (Table 2, entry 8).

**Scheme 5:**
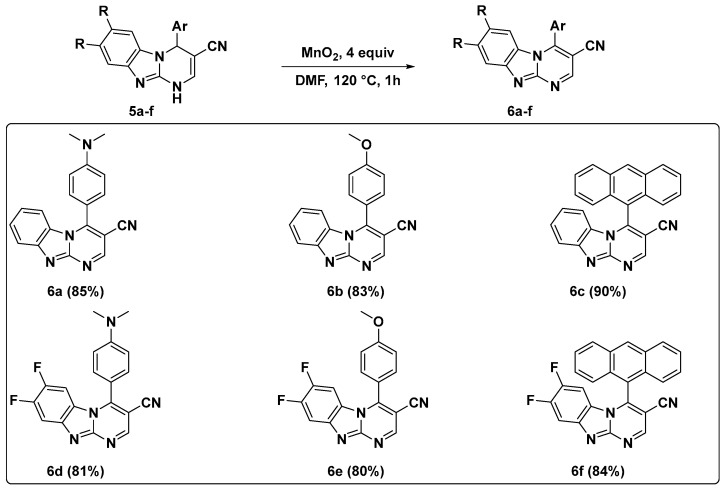
Scope of the 4-(aryl)benzo[4,5]imidazo[1,2-*a*]pyrimidine-3-carbonitriles **6a**–**f**.

This newly developed methodology was then used to synthesize a series of new 4-(aryl)benz[4,5]imidazo[1,2-a]pyrimidine-3-carbonitriles **6b**–**f** with yields in the range of 80–90% (Scheme 5).

All derivatives **6a**–**f** were obtained with comparable yields, which indicates an insignificant influence of the nature of the substituents on the oxidation process. All the synthesized compounds were fully characterized by ^1^H-NMR, ^13^C-NMR, IR-spectroscopy, and elemental analysis (Supplementary Materials). In particular, in the ^1^H-NMR spectra, the aromatic proton signals were observed at δ 5.00–9.41 ppm, whereas the aliphatic proton signals were observed at δ 3.11–3.96 ppm. In the ^13^C-NMR spectra, (hetero)aryl carbon nuclei are located at δ 94.6–162.2 ppm, while signals corresponding to aliphatic carbon were observed at δ 39.6–55.6 ppm. It should be emphasized that for the difluoro derivatives **6d**–**f** in both the ^1^H- and ^13^C-NMR spectra, a characteristic multiplicity was observed, due to the spin–spin interaction of the H-F and C-F nuclei. It is also interesting that in the IR spectra of compounds **6a**–**f**, the characteristic stretching vibrations of (-C≡N) bonds at ν 2227–2230 cm^−^^1^ were observed.

**Figure 1:**
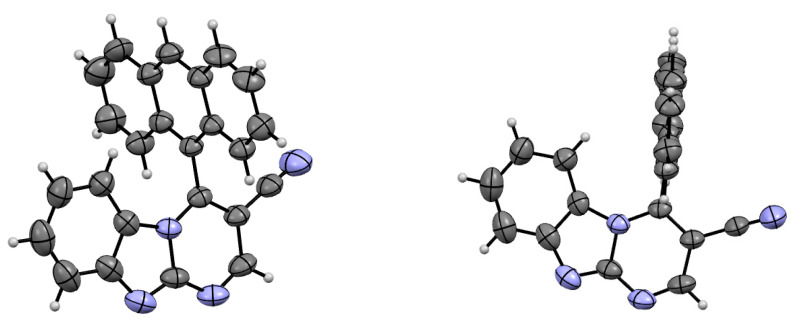
Molecular structure of **6c**.

As previously stated, an unprecedented in situ [1,3] sigmatropic rearrangement was reported for the related *N*-10 substituted systems. However, the spectral data obtained for compounds **5a**–**f** and **6a**–**f** do not allow one to determine the position of the Ar substituent in the dihydropyrimidine system with certainty. Single crystal X-ray diffraction analysis was performed on compound **6c** to confirm the structure of the obtained compound and to prove the hypotheses about the possibility of rearrangement in the case of unsubstituted benzimidazole-2-arylimine (Figure 1).

According to the XRD data, in compound **6c**, the (Ar) substituent is located in the position of C4 of the pyrimidine ring, which indicates the possibility of the rearrangement in the herein reported systems.

**Scheme 6:**
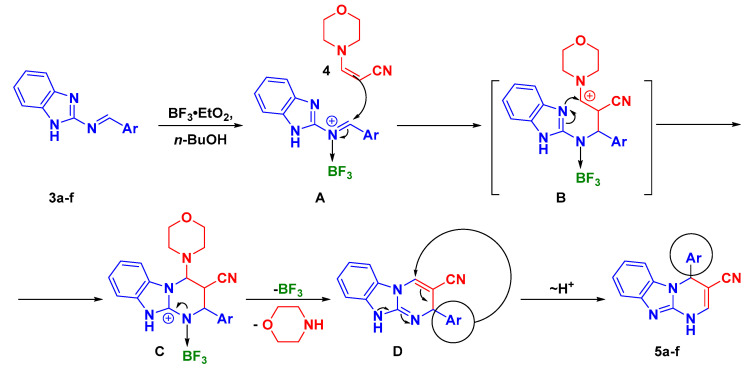
Plausible reaction mechanisms of dihydropyrimidines **5a**–**f** formation and [1,3] sigmatropic rearrangement.

The proposed mechanism of the interaction between benzimidazole-2-arylimines **3a**–**f** and 3-morpholinoacrylonitrile (**4**), based on the reactivity of these substrates and literature data [23,26,49], is shown in Scheme 6.

As a first stage, the benzimidazole-2-arylimines **3a**–**f** are activated via the interaction with BF_3_∙Et_2_O, resulting in the formation of activated complex **A.** At the next stage, there is an asynchronous concerted process interaction of the intermediate **A** with 3-morpholinoacrylonitrile (**4**) through an ephemeral transition state **B** resulting in the formation of a tetrahydropyrimide system **C**. The removal of the morpholine molecule results in system **D**, which undergoes [1,3] sigmatropic rearrangement and yields derivatives **5a**–**f**.

In addition, we discovered that all of the 4-(aryl)benzo[4,5]imidazo[1,2-a]pyrimidine-3-carbonitriles **6a**–**f** obtained are fluorescent in solution and solid form. Therefore, photophysical studies of the obtained products **6a**–**f** were carried out.

### 2.2. Photophysical Studies

#### 2.2.1. Absorption/Fluorescence Studies in Solution and Solvent Effect

All the obtained fluorophores were soluble in concentrations less than 2 × 10^−5^ M both in nonpolar (cyclohexane and toluene) and in weakly and strongly apolar aprotonic solvents (THF, acetonitrile, DMSO). Additionally, all the compounds have exhibited an intense fluorescence in solution. Taking into account the subsequent study of the phenomenon of aggregation-induced emission (AIE), THF, which is located at the interface between nonpolar and polar solvents with an average value of orientational polarizability, was chosen as the optimal aprotonic solvent (Δf = 0.21). The results of the photophysical studies are presented below (Table 3).

**Figure 2:**
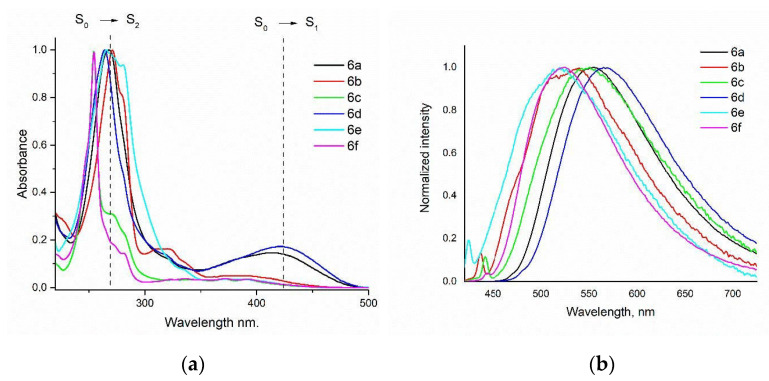
Absorption (**a**) and emission (**b**) spectra of fluorophores **6a**–**f** in THF (c = 10^−5^ M).

Emission spectra for all the compounds were measured at low concentrations of 10^−5^ M to avoid any concentration-dependent dimerization and fluorescence quenching. All the graphs were normalized for comparative analysis (Figure 2).

The absorption spectra of the fluorophores **6a**–**f** are presented by two absorption bands with different intensities at maximum wavelengths in the 220–300 nm and 350–500 nm ranges, which correspond to S_0_→S_2_ and S_0_→S_1_ transitions. In this case, all the compounds show a dominant absorption band due to the transition S_0_ → S_2_ with εM < 14.5 × 104 M^−1^ cm^−1^.

The emission spectra of the fluorophores **6a**–**f** are presented by the solid unstructured emission bands with maximums from 520 to 567 nm, referring to the excited ICT-state in a polar aprotic solvent [36]. A significant bathochromic shift was observed for the two 2-dimethylaminophenyl substituted imidazopyrimidine fluorophores **6a**,**d**, which have some of the most energetically favorable states among the obtained series of fluorophores (3.39 eV for **6a** and 3.24 eV for **6b**) (See Section 2.2.5. Theoretical Calculations). The fluorescence lifetimes of the investigated compounds **6a**–**f** exhibited a two-exponential decay in THF. The lifetime of the excited state of the fluorophores was measured at r.t. in THF using a nanosecond LED with an excitation wavelength of 370 nm. The average lifetime was calculated using the expression τ_av_ = Σ (τ_i_ × α_i_) (Table S1). Overall, the average fluorescence lifetime (τ_av_) ranged from 1.59 ns (lowest for **6d**) to 8.78 ns (highest for **6c**) (Table 3). The compounds were characterized by large Stokes shift values (<140 nm), while the quantum yield values in THF were not higher than 7.5%.

Compounds **6a**–**f** with variation of electron-donating fragments (4-methoxyphenyl, 4-(dimethylamino)phenyl and anthracen-9-yl) based on the 3-cyanosubstituted benzo [4,5]imidazo [1,2-*a*]pyrimidine, including those substituted with fluorine atoms in positions 7,8 implies that the solvent polarity may influence the electronic state properties of the chromophore (See Section 2.2.5. Theoretical Calculations).

We studied the emission characteristics of **6a**–**f** compounds in various solvents (Tables S2–S7). Indeed, the effect of the solvent polarity was observed for the chromophores of the entire series with Stokes shifts from 120 to 180 nm. However, only for anthracenyl substituted fluorophores, upon the increasing solvent polarity in a row from nonpolar cyclohexane to the polar DMSO and MeCN, the emission bands of the fluorophores **6c**,**f** became broad and significantly shifted to the red region, which agrees with the character of strong intramolecular charge transfer (ICT) and is confirmed by the values of theoretically calculated descriptors. Interestingly, in a study of the AIE effect, fluorophore **6f** showed a solvatochromic shift in the THF—water binary system of 10–90% water content in the 520–610 nm wavelength range (Figure S3).

#### 2.2.2. Solid State Fluorescence Studies

**Figure 3:**
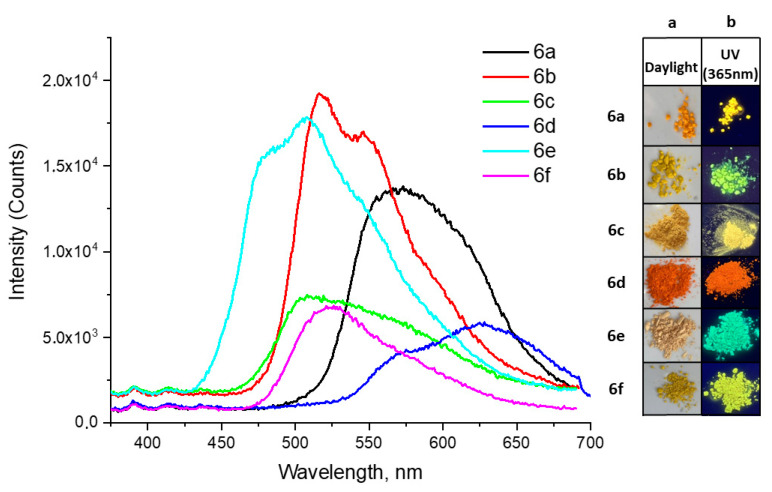
Emission spectra of dyes **6a**–**f** in powders (**a**) and photographs of the solid samples under daylight and 365 nm UV irradiation (**b**).

**Figure 4:**
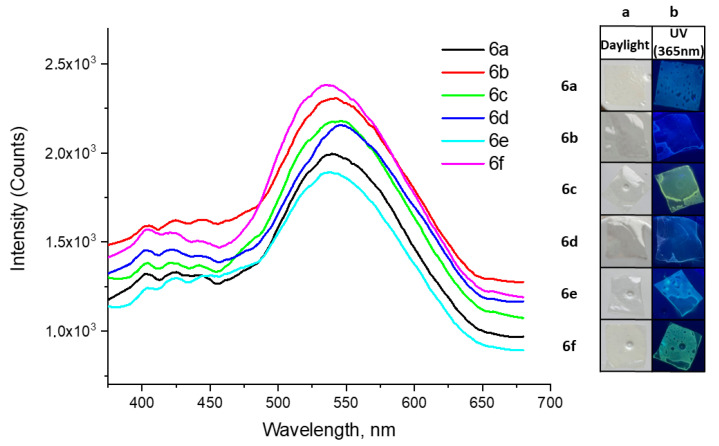
Emission spectra of dyes **6a**–**f** in PVA films (**a**) and photographs of the samples under daylight and 365 nm UV irradiation (**b**).

The emission spectra of fluorophores **6a**–**f** in the powder/film as well as the experimental data are presented in Table 4 and Figure 3 and Figure 4. Interestingly, only the dimethoxyphenyl-substituted fluorophores **6a** and **6d** exhibited a redshifted emission in a powder when compared to the spectra in THF solution, implying specific π-π interactions in the solid state.

In the manufacture of OLED devices, thin films of compounds are applied in layers; therefore, it is necessary to conduct optical studies with thin films of materials [54]. To examine the emission in the films, thin films of PVA with integrated fluorophores **6** were deposited on quartz plates, and their emission spectra were measured by using the integrating sphere. In all the spectra, the emission maxima were observed at about 545 nm and were quite similar to the ones collected in THF solution. Thus, the absence of an anomalous red shift in the solid emission demonstrates the useful role of the cyano-group in the phenylimidazopyridine chromophore for restraining the formation of heavy J-aggregates in the solid state [55]. In contrast to the emission in powder, the **6b**–**f** samples in the PVA film showed a significant improvement in fluorescence along with an up to 50% increase in quantum yields, which demonstrates the existence of AIE effects similar to those in solutions.

#### 2.2.3. Aggregation Studies

**Figure 5:**
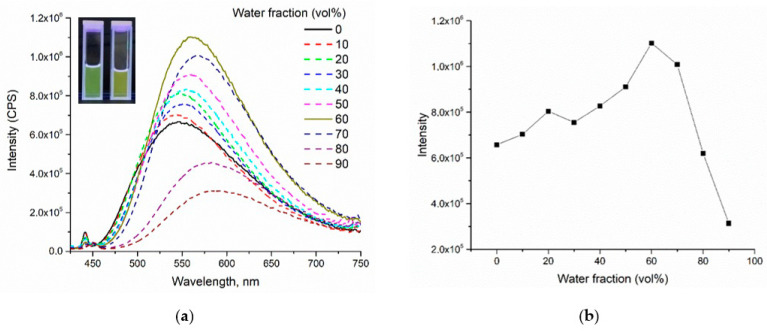
Emission spectra of **6c** in different ratios of THF–water (*v*/*v*) mixtures (**a**). Plot of I/I_0_ versus water fraction (vol%), where I_0_ is the fluorescence intensity in pure THF and emission images of the **6c** in different water fraction mixtures under 365 nm UV illumination (λ_ex_ = 365 nm) with the concentration of 10^−5^ M (**b**).

The phenomenon of aggregation-induced emission (AIE) is usually associated with the well-known Mie scattering effect and is a signal of nanoaggregate formation [56]. The AIE properties of the **6a**–**f** dyes were investigated using different water fractions (fw) in THF. As shown in Table 3, anthracenyl substituted fluorophore **6c** almost does not emit in pure THF with a fluorescence quantum yield of less than 0.1%. However, when the water content in the THF solution was increased to 60%, a new green emission band with a maximum at 555 nm was observed for this dye. At the same time, the emission intensity increased approximately two-fold. In addition, the absorption spectra of **6c** with a water fraction of 60% did not coincide with the spectra of pure THF and contained an additional absorption peak in the 425–500 nm range, which may be associated with light scattering due to the formation of nanoaggregates (Figure 5) [57]. In addition, the time-resolved fluorescence curves of **6c** in pure THF and with a water fraction of 60% did not coincide (Table S8, Figure 5). Apparent changes in the mean fluorescence lifetime (τ_av_) of **6c** from 6.9 ns in THF to 8.8 ns after the addition of water were observed. The experimental results of the effect of the nature of solvents and the values of the theoretically calculated descriptors are consistent with the fluorescence enhancement behavior of **6c** and indicate that the AIE process is accompanied by the formation of molecular aggregates. The optimized **6c** geometries for the ground and excited states in the THF were calculated to interpret the AIE process (See Section 2.2.5. Theoretical Calculations).

#### 2.2.4. Mechanochromic Properties

In general, non-planar push-pull luminophores with AIE properties tend to show mechanochromic response [58]. As shown above, fluorophores **6c**,**f** turned out to be AIE-active; their emission maximums were different in the solid state and in aggregate (Table 3 and Table 4); therefore, these two fluorophores were selected as the most suitable candidates for the study of mechanochromic properties. As crystalline samples, anthracenyl substituted fluorophores **6c** and **6f** were obtained with low emission intensities, QYs of 3.9% (6c) and 3.4% (6f), and emission maxima of 511 and 525 nm, respectively (Table 3).

**Figure 6:**
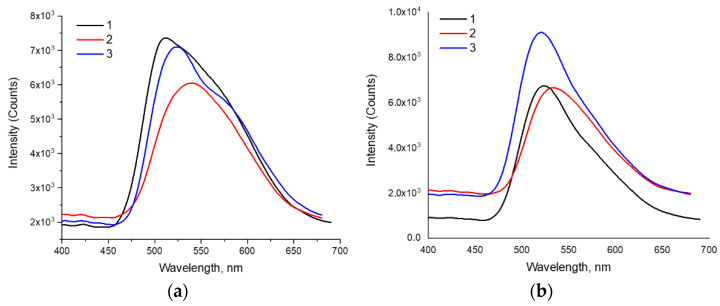
(**a**) Emission spectra of **6c** in solid states (λ_ex_ = 350 nm): as prepared (1), after grinding (2), and after treatment with CH_2_Cl_2_ (3). (**b**) Emission spectra of **6f** in solid states (λ_ex_ = 350 nm): as prepared (1), after grinding (2), and after treatment with CH_2_Cl_2_ (3).

**Figure 7:**
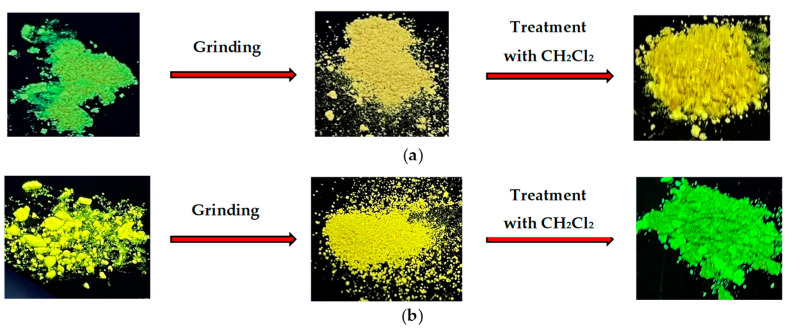
Photographs of **6c** (**a**) and **6f** (**b**) taken under 365 nm UV irradiation.

After grinding with a mortar and pestle, the fluorescence emission of compounds **6c** and **6f** was measured. As it turned out, the compounds demonstrated different responses to mechanical (grinding) stimulation. Thus, the grinding of the yellow powder **6c** led to a red-shift of the fluorescence spectra by 31 nm (the red line) and a decrease in fluorescence intensity (Figure 6a and Figure 7a, Table S9). Additionally, after the resuspension of the sample from CH_2_Cl_2_, yellow crystals were formed (Figure 7a) and a slight shift of the emission peak to the blue region was recorded.

The **6f** derivative was obtained as yellow crystals with poor emission intensity (Table 3). The grinding of the crystals of 6f resulted in a bright yellow powder (Figure 7b), along with a low red-shift of the fluorescence by 10 nm (the red line) with the same fluorescence intensity. (Figure 6b). Interestingly, after resuspension of the sample in CH_2_Cl_2_, a mixture of crystals and powder formed, as well as a slightly blue-shifted emission peak that increased with fluorescence intensity (Figure 6b and Figure 7b, Table S9).

Most probably, the fluorescence response of the samples **6c** and **6f** during grinding depends on the molecular stacking structures in the solid state [59].

#### 2.2.5. Theoretical Calculations

The DFT-calculations were performed in order to evaluate the donor-acceptor properties and the nature of intramolecular charge transfer based on the obtained optimized model structures of fluorophores **6a**–**f** in the ground and excited states in the solvent phase, energy levels and electron density distribution in frontier molecular orbitals (FMOs), and descriptors—charge-transfer indices (CT-indexes).

**Figure 8:**
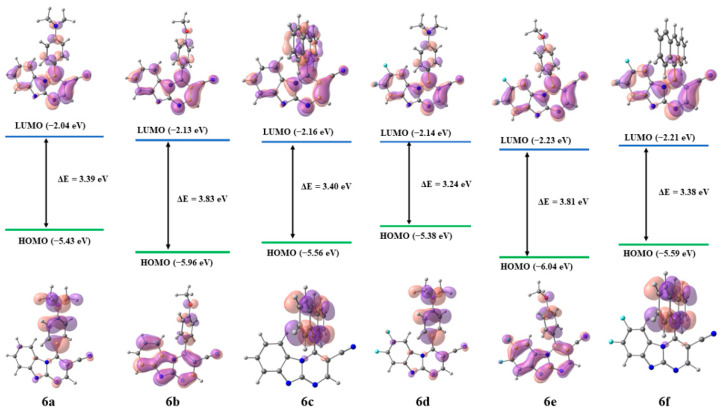
Energy gaps of fluorophores **6a**–**f** in THF phase.

The electron density distributions of the boundary molecular orbitals of FMO **6a**–**f** are shown in Figure 8 and in Table 5.

The highest occupied molecular orbitals (HOMOs) of the anthracenyl substituted dyes **6c**,**f** delocalized exclusively on the donor group, whereas the acceptor group based on the 3-cyano substituted benzo [4,5]imidazo [1,2-*a*]pyrimidine domain is responsible for the contribution to the lowest unoccupied molecular orbitals (LUMOs). Charge delocalization was less pronounced in the electron density distribution in the FMO for dimethylaminophenyl substituted fluorophores **6a**,**d**. In fact, there was no delocalization of electron density for methoxyphenyl substituted samples **6b**,**e**.

**Figure 9:**
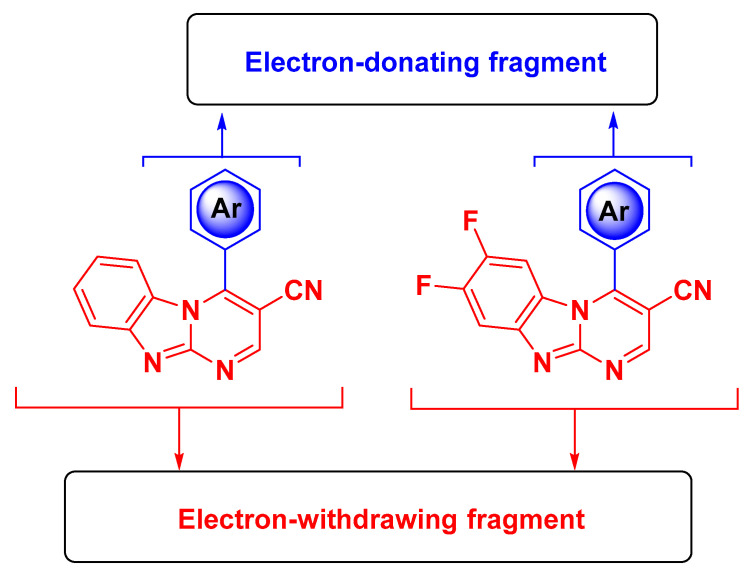
Donor-acceptor structure 4-aryl-substituted benzo[4,5]imidazo[1,2-*a*]pyrimidine chromophors **6a**–**f**.

Thus, based on theoretical calculations and experimental data, one can present a general model of the studied fluorophores consisting of a donor methoxyphenyl/dimethylaminophenyl/anthracenyl fragment (Ar, blue) and an acceptor 3-cyano-substituted benzo[4,5]imidazo[1,2-*a*]pyrimidine domain (red), including substituted fluorine atoms at positions 7 and 8 (Figure 9).

To obtain a deeper understanding of the correlation between charge transfer and fluorophore structures, additional calculations of CT-indices were performed [60]. The corresponding indices (D, S_r_, and t) presented in Table 6 were calculated for all fluorophores in the Multiwfn program [61].

The highest D index values [60], as the distance between the centers of gravity of the donor and acceptor, were 4.4 and 4.5 Å for anthracenyl substituted **6c** and **6f**, respectively, which result from the highest degree of intramolecular charge transfer. The S_r_ index introduced by Tozer in 2008 [62] gives a good correlation between the value of the Stokes shift and the CT junction value; that is, the smaller S_r_ corresponds to the larger Stokes shifts. The lowest values of this index correspond to compounds **6c**,**f**, as confirmed by studies of the solvatochromic effect with the highest values of the 168–181 nm Stokes shift. The index t > 0 confirms the very fact of charge separation (CD) between the chromophore donor and acceptor due to charge excitation. Thus, the analysis of CT indices confirmed the ICT process for the anthracenyl and dimethylaminophenyl substituted chromophores **6a**,**d** and **6c**,**f**, and also made it possible to predict a significant overlap between the centroids of the positive charge of the donor and the negative charge of the acceptor, representing the zones of increase and decrease in electron density upon excitation, based on the calculated values of D at t > 0.

### 2.3. Crystallography

**Figure 10:**
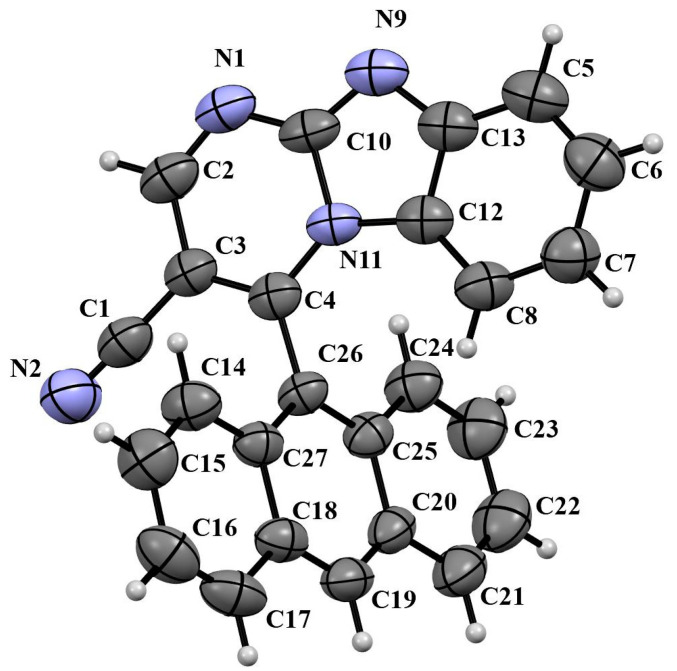
The compound **6c** in the thermal ellipsoid at the 50% probability level.

**Figure 11:**
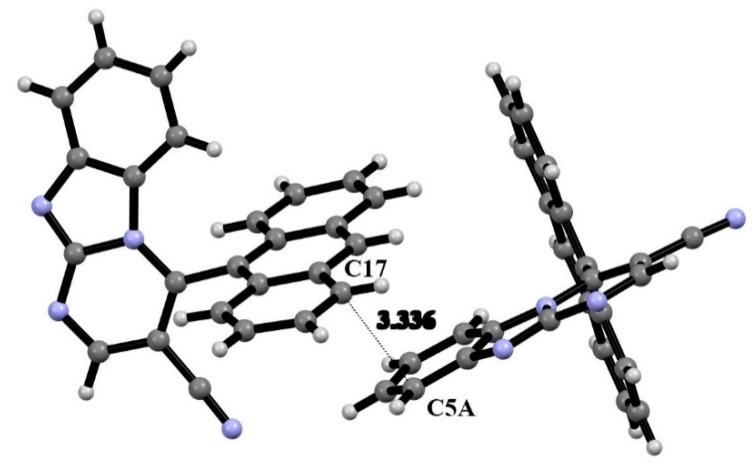
π-π-contacts in the crystal of the compound **6c**.

According to the XRD data, two independent molecules of the compound **6c** crystallize with a molecule of CH_2_Cl_2_ in the centrosymmetric space group of the triclinic system. In the result, the structurally independent unit C_51_H_30_Cl_2_N_8_ (M = 825.73 g/mol) was used for all calculations. The molecule CH_2_Cl_2_ is disordered and demonstrates the high magnitude of the anisotropic displacement parameters. The geometry of independent heterocyclic molecules differs only slightly, primarily in the dihedral angles between the heterocyclic and anthracene planes. The general geometry of the molecule was shown in Figure 10. The mean bond distances and angles in the molecules are close to expectations. The heterocyclic and anthracene parts of the molecule are non-conjugated due to high dihedral angles between their planes. In the crystal some polar CArH…NC- contacts are observed with participation of the CN-group, in particular, H(9A)…N(2) [x − 1, y + 1, z] 2.66 Å (on a scale of 0.09 Å less than the sum of the VdW radii) and N(2A)…H(19A) [−x, 1 − y, 1 − z] 2.71 Å (on the order of 0.04 Å less than the sum of the VdW radii ). The π-π-contacts in the crystal are presented only as shortened π-π-contact between the heterocycle and anthracene moiety C(5A)…C (17) at a distance of 3.336(4) Å (0.064 Å less than the sum of the VdW radii, Figure 11).

## 3. Materials and Methods

### 3.1. Chemical Experiment

Commercial reagents were obtained from Sigma-Aldrich, Acros Organics, or Alfa Aesar and used without any preprocessing. All workup and purification procedures were carried out using analytical-grade solvents. One-dimensional ^1^H- and ^13^C-NMR spectra were acquired on a Bruker DRX-400 instrument (Karlsruhe, Germany) (400 and 101 MHz, respectively), utilizing DMSO-*d*_6_, CDCl_3_, and CF_3_COOD as solvents and an external reference, respectively. Chemical shifts are expressed in δ (parts per million, ppm) values, and coupling constants are expressed in hertz (Hz). The following abbreviations are used for the multiplicity of NMR signals: s, singlet; d, doublet; t, triplet; dd, doublet of doublet; m, multiplet; and AN, anthracene. IR spectra were recorded on a Bruker α spectrometer equipped with a ZnSe ATR accessory. Elemental analysis was performed on a PerkinElmer PE 2400 elemental analyzer (Waltham, MA, USA). Melting points were determined on a Stuart SMP3 (Staffordshire, UK) and are uncorrected. The monitoring of the reaction progress was performed using TLC on Sorbfil plates (Imid LTD, Russia, Krasnodar) (the eluent is EtOAc). The spectral characteristics of the compound **3b** correspond to the data [63]. The compound 3-Morpholinoacrylonitrile (**4**) was prepared according to a literature procedure [64].

General procedure for the synthesis of N-(4-arylidene)-1H-benzo[d]imidazol-2-amine (**3a**,**c** and **3d**–**f**).

Corresponding 1*H*-benzo[*d*]imidazol-2-amine **1a**,**b** (0.01 mol) was mixed with corresponding aldehydes **2a**,**c** and **2d**–**f** (0.0105 mol) and the mixture was heated at 130 °C for 3 h. The reaction mixture was cooled to room temperature and ground up to give the expected pure product.

4-Dimethylaminobenzylidene-1H-benzo[*d*]imidazol-2-amine (**3a**). Yellow powder (2.37 g, yield 90%), m.p. 245–247 °C. FT-IR (neat) ν_max_ (cm^−1^): 3051, 1614, 1584, 1443, 1415, 1167. ^1^H-NMR (400 MHz, DMSO-*d_6_*) *δ* (ppm) 3.04 (6H, s, -N(CH_3_)_2_), 6.82 (2H, d, *J* = 8.6 Hz, H-2′, H-6′), 7.09–7.15 (2H, m, H-5, H-6), 7.29–7.44 (1H, m, H-4), 7.43–7.57 (1H, m, H-7), 7.86 (2H, d, *J* = 8.4 Hz, H-3′, H-5′), 9.24 (1H, s, N=CH), 12.36 (1H, s, NH). ^13^C{^1^H}-NMR (100 MHz, DMSO-*d*_6_) *δ* (ppm) 40.1 (2C), 66.8, 111.1, 112.1 (2C), 118.5, 121.8 (2C), 123.0, 132.0, 134.6, 143.1, 153.8, 157.5, 164.9 Calcd for C_16_H_16_N_4_: C 72.70, H 6.10, N 21.20; found: C 72.63, H 6.15, N 21.22.

N-(Anthracen-9-ylidene)-1H-benzo[*d*]imidazol-2-amine (**3c**). Orange powder (2.73 g, yield 85%), m.p. 277–279 °C. FT-IR (neat) ν_max_ (cm^−1^): 3046, 1790, 1620, 1553, 1517, 1338. ^1^H-NMR (400 MHz, DMSO-*d_6_*) *δ* (ppm) 7.15–7.22 (2H, m, H-5, H-6), 7.44–7.51 (1H, m, H-4), 7.56–7.65 (3H, m, H-7, 2xH_AN_), 7.67–7.73 (2H, m, 2xH_AN_), 8.17 (2H, d, *J* = 8.3 Hz, 2xH_AN_), 8.80 (1H, s, H_AN_), 9.13 (2H, d, *J* = 9.0 Hz, 2xH_AN_), 10.74 (1H, s, N=CH), 12.73 (1H, s, NH). ^13^C{^1^H}-NMR (100 MHz, DMSO-*d*_6_) *δ* (ppm) 111.2, 118.9, 122.1, 124.6, 124.8, 125.8, 128.3, 129.4, 130.9, 131.0, 132.9, 134.4, 142.5, 156.1, 163.9. Calcd for C_22_H_15_N_3_: C 82.22, H 4.70, N 13.08; found: C 82.25, H 4.66, N 13.03.

5,6-Difluoro-N-(4-dimethylaminobenzylidene)-1H-benzo[*d*]imidazol-2-amine (**3d**). Yellow powder (2.61 g, yield 87%), m.p. 294–296 °C. FT-IR (neat) ν_max_ (cm^−1^): 3045, 1636, 1614, 1549, 1353, 1155. ^1^H-NMR (400 MHz, DMSO-*d_6_*) *δ* (ppm) 3.07 (6H, s, -N(CH_3_)_2_), 6.83 (2H d, *J* = 8.6 Hz, H-2′, H-6′), 7.31–7.61 (2H, m, H-4, H-7), 7.86 (2H, d, *J* = 8.5 Hz, H-3′, H-5′), 9.19 (1H, s, N=CH), 12.59 (1H, s, NH). ^13^C{^1^H}-NMR (100 MHz, DMSO-*d*_6_) *δ* (ppm) 31.2 (2C), 99.2, 105.9, 112.1 (2C), 122.7, 129.9 (d, *J* = 8.9 Hz) 132.2 (2C), 138.7 (d, *J* = 9.7 Hz), 144.6 (d, *J* = 249.0 Hz), 145.1 (d, *J* = 227.2 Hz), 154.0 (2C), 159.2, 165.3. ^19^F-NMR (376 MHz, DMSO-*d_6_*) *δ* (ppm) −145.9 (d, *J* = 22.2 Hz), -145.14 (d, *J* = 20.8 Hz). Calcd for C_16_H_14_F_2_N_4_: C 63.99, H 4.70, N 18.66; found: C 63.81, H 4.73, N 18.53.

5,6-Difluoro-N-(4-methoxybenzylidene)-1H-benzo[*d*]imidazol-2-amine (**3e**). Yellow powder (2.58 g, yield 90%), m.p. 254–256 °C. FT-IR (neat) ν_max_ (cm^−1^): 3062, 1593, 1568, 1509, 1453, 1256. ^1^H-NMR (400 MHz, DMSO-*d_6_*) *δ* (ppm) 3.86 (3H, s, OCH_3_), 7.12 (2H d, *J* = 8.4 Hz, H-2′, H-6′), 7.37–7.62 (2H, m, H-4, H-7), 8.01 (2H, d, *J* = 8.3 Hz, H-3′, H-5′), 9.32 (1H, s, N=CH), 12.79 (1H, s, NH). ^13^C{^1^H}-NMR (100 MHz, DMSO-*d*_6_) *δ* (ppm) 55.6, 99.0, 105.8, 114.6 (2C), 127.8, 129.7, 137.7 (2C), 138.0, 146.5 (d, *J* = 237.6 Hz), 146.6 (d, *J* = 237.2 Hz), 157.8, 163.2, 165.1. ^19^F-NMR (376 MHz, DMSO-*d_6_*) *δ* (ppm) -145.3, -144.3. Calcd for C_15_H_11_F_2_N_3_O: C 62.72, H 3.86, N 13.23; found: C 62.65, H 3.91, N 13.17.

5,6-Difluoro-N-(Anthracen-9-ylidene)-1H-benzo[*d*]imidazol-2-amine (**3f**). Orange powder (3.00 g, yield 84%), m.p. 282–284 °C. FT-IR (neat) ν_max_ (cm^−1^): 3145, 1666, 1553, 1479, 1452, 1199. ^1^H-NMR (400 MHz, DMSO-*d_6_*) *δ* (ppm) 7.52–7.76 (6H, m, H-4, 5xH_AN_), 8.20 (2H, d, *J* = 8.4 Hz, 2xH_AN_), 8.89 (1H, s, H_AN_), 8.96–9.09 (2H, m, H-7, H_AN_), 10.63 (1H, s, N=CH), 13.15 (1H, s, NH). ^13^C{^1^H}-NMR (100 MHz, DMSO-*d*_6_) *δ* (ppm) 99.9 (d, *J* = 22.5 Hz), 106.7 (d, *J* = 19.8 Hz), 124.0, 125.0, 126.3, 128.9, 129.76, 129.83, 129.9, 130.4 (d, *J* = 11.5 Hz), 131.2, 131.4, 131.6, 131.9, 133.7, 135.7, 138.6 (d, *J* = 10.9 Hz), 147.2 (d, *J* = 237.7 Hz), 147.4 (d, *J* = 238.0 Hz), 158.2, 164.9, 194.7. ^19^F-NMR (376 MHz, DMSO-*d_6_*) *δ* (ppm) -144.7 (d, *J* = 21.8 Hz), -143.54 (d, *J* = 22.1 Hz). Calcd for C_22_H_13_F_2_N_3_: C 73.94, H 3.67, N 11.76; found: C 74.03, H 3.53, N 11.46.

General procedure for the synthesis of 4-(aryl)-1,2-dihydrobenzo[4,5]imidazo[1,2-*a*]pyrimidine-3-carbonitriles (**5a**–**c**).

To a suspension of the corresponding derivative **3a**–**c** (0.01 mol, 1 equivalent) in 30 mL of *n*-BuOH, 1.88 mL (0.015 mol., 1.5 equiv.) of BF_3_·Et_2_O was added. To the resulting solution, 1.38 g (0.01 mol, 1 equivalent) of 3-morpholinoacrylonitrile (**4**) was added. The reaction mixture was heated in an oil bath at 130 °C for 5 h. The resulting mixture was cooled to room temperature and stirred for 15 min. The obtained precipitate was filtered off and washed with *i*-PrOH, water, and acetone to give the expected pure product.

4-(Dimethylaminophenyl)-1,2-dihydrobenzo[4,5]imidazo[1,2-*a*]pyrimidine-3-carbonitrile (**5a**). White powder (2.33 g, yield 74%), m.p. > 300 °C. FT-IR (neat) ν_max_ (cm^−1^): 3071, 2805, 2215, 1621, 1578, 1459. ^1^H-NMR (400 MHz, CDCl_3_ + 0.1 mL CF_3_COOD) *δ* (ppm) 3.36 (6H, s, -N(CH_3_)_2_), 6.42 (1H, s, H-4), 6.97 (1H, d, *J* = 8.3 Hz, H-6), 7.35 (1H, t, *J* = 7.9 Hz, H-7), 7.50–7.56 (2H, m, H-8, H-2), 7.64–7.73 (5H, m, H-9, H-2′, H-3′, H-5′ H-6′), 11.30 (1H, s, -NH). ^13^C{^1^H}-NMR (100 MHz, CDCl_3_ + 0.1 mL CF_3_COOD) *δ* (ppm) 47.6 (2C), 57.3, 87.6, 111.5, 114.1, 114.4, 122.4 (2C), 126.7, 127.3, 127.8, 128.6, 129.7 (2C), 135.8, 138.4, 141.6, 143.6. Calcd for C_19_H_17_N_5_: C 72.36, H 5.43, N 22.21; found: C 72.45, H 5.51, N 22.04.

4-(4-Methoxyphenyl)-1,2-dihydrobenzo[4,5]imidazo[1,2-*a*]pyrimidine-3-carbonitrile (**5b**). White powder (1.90 g, yield 63%), m.p. 270–272 °C. FT-IR (neat) ν_max_ (cm^−1^): 3376, 3109, 2209, 1659, 1624, 1254. ^1^H-NMR (400 MHz, CDCl_3_ + 0.1 mL CF_3_COOD) *δ* (ppm) 3.82 (3H, s, OCH_3_), 6.16 (1H, s, H-4), 6.89–7.05 (3H, m, H-6, H-3′, H-5′), 7.25 (1H, t, *J* = 7.8 Hz, H-7), 7.28–7.36 (3H, m, H-2, H-2′, H-6′), 7.40 (1H, t, *J* = 7.8 Hz, H-8), 7.62 (1H, d, *J* = 8.2 Hz, H-9). ^13^C{^1^H}-NMR (100 MHz, CDCl_3_ + 0.1 mL CF_3_COOD) *δ* (ppm) 55.6 (2C), 58.1, 89.3, 111.9, 114.0, 115.4 (2C), 125.4, 126.5, 127.5, 128.0, 128.6 (2C), 129.4, 133.8, 142.6, 161.3. Analytical calculated for C_18_H_14_N_4_O: C 71.51, H 4.67, N 18.53; found: C 71.58, H 4.61, N 18.45.

4-(Anthracen-9-yl)-1,2-dihydrobenzo[4,5]imidazo[1,2-*a*]pyrimidine-3-carbonitrile (**5c**). White powder (2.23 g, yield 60%), m.p. > 300 °C. FT-IR (neat) ν_max_ (cm^−1^): 3062, 2635, 2202, 1666, 1502, 1447. ^1^H-NMR (400 MHz, CDCl_3_ + 0.1 mL CF_3_COOD) *δ* (ppm) 6.11 (1H, d, *J* = 8.4 Hz, H_AN_), 6.86 (1H, t, *J* = 8.0 Hz, H-6), 7.21– 7.27 (1H, m, H-7), 7.42–7.55 (3H, m, H-2, 2xH_AN_), 7.60 (1H, s, H-4), 7.64–7.70 (1H, m, H-8), 7.76–7.85 (2H, m, 2xH_AN_), 7.97 (1H, s, H_AN_), 8.05–8.12 (1H m, H_AN_), 8.21 (1H, d, *J* = 8.5 Hz, H-9), 8.49 (1H, d, *J* = 9.1 Hz, H_AN_), 8.72 (1H, s, H_AN_). ^13^C{^1^H}-NMR (100 MHz, CDCl_3_ + 0.1 mL CF_3_COOD) *δ* (ppm) 53.3, 88.8, 112.0, 113.8, 114.1, 120.3, 120.5, 121.5, 125.9, 126.1, 126.3, 127.0, 128.2, 128.3, 129.1, 129.9, 130.3, 130.9, 131.2, 131.3, 131.5, 132.0, 133.4, 135.5, 141.8. Calcd for C_25_H_16_N_4_: C 80.63, H 4.33, N 15.04; found: C 80.53, H 4.42, N 15.05.

4-(4-(Dimethylamino)phenyl)-7,8-difluoro-1,2-dihydrobenzo[4,5]imidazo[1,2-*a*]pyrimidine-3-carbonitrile (**5d**). White powder (2.28 g, yield 65%), m.p. > 300 °C. FT-IR (neat) ν_max_ (cm^−1^): 3106, 2886, 2216, 1658, 1495, 1463. ^1^H-NMR (400 MHz, DMSO-*d_6_*) *δ* (ppm) 2.88 (6H, s, -N(CH_3_)_2_), 6.25 (1H, s, H-4), 6.69 (2H, d, *J* = 8.3 Hz, H-3′, H-5′), 6.98 (1H, dd, *J* = 10.5, 7.3 Hz, H-6), 7.20 (2H, d, *J* = 8.3 Hz, H-2′, H-6′), 7.42 (1H, dd, *J* = 11.2, 7.3 Hz, H-9), 7.58 (1H, s, H-2), 11.14 (1H, s, -NH). ^13^C{^1^H}-NMR (100 MHz, CDCl_3_ + 0.1 mL CF_3_COOD) *δ* (ppm) 47.4 (2C), 57.5, 87.7, 101.1 (d, *J* = 25.4 Hz), 104.2 (d, *J* = 24.1 Hz), 113.8, 122.7 (2C), 123.1 (d, *J* = 12.7 Hz), 124.6 (d, *J* = 13.1 Hz), 129.8 (2C), 135.6, 137.8, 142.9, 143.9, 149.7 (dd, *J* = 253.1, 15.0 Hz), 150.5 (dd, *J* = 251.6, 15.4 Hz). Calcd for C_19_H_15_F_2_N_5_: C 64.95, H 4.30, N 19.93; found: C 64.78, H 4.47, N 19.87.

7,8-Difluoro-4-(4-methoxyphenyl)-1,2-dihydrobenzo[4,5]imidazo[1,2-*a*]pyrimidine-3-carbonitrile (**5e**). White powder (1.99 g, yield 59%), m.p. > 300 °C. FT-IR (neat) ν_max_ (cm^−1^): 3109, 2213, 1586, 1462, 1374, 1252. ^1^H-NMR (400 MHz, CDCl_3_ + 0.1 mL CF_3_COOD) *δ* (ppm) 3.84 (3H, s, OCH_3_), 6.09 (1H, s, H-4), 6.76–6.83 (1H, m, H-6), 6.98 (2H, d, *J* = 8.6 Hz, H-3′, H-5′), 7.28–7.35 (3H, m, H-2, H-2′, H-6′), 7.48–7.55 (1H, m, H-9). ^13^C{^1^H}-NMR (100 MHz, CDCl_3_ + 0.1 mL CF_3_COOD) *δ* (ppm) 55.6, 58.4, 89.4, 101.4 (d, *J* = 24.5 Hz), 103.5 (d, *J* = 24.1 Hz), 114.9, 115.7 (2C), 123.7 (d, *J* = 10.2 Hz), 125.5 (d, *J* = 11.8 Hz), 126.7, 128.6 (2C), 133.5, 143.8, 148.7 (dd, *J* = 249.3, 14.4 Hz), 149.6 (dd, *J* = 249.4, 13.7 Hz), 161.6. Calcd for C_18_H_12_F_2_N_4_O: C 63.90, H 3.58, N 16.56; found: C 63.83, H 3.61, N 16.38.

4-(Anthracen-9-yl)-7,8-difluoro-1,2-dihydrobenzo[4,5]imidazo[1,2-*a*]pyrimidine-3-carbonitrile (**5f**). White powder (2.57 g, yield 63%), m.p. > 300 °C. FT-IR (neat) ν_max_ (cm^−1^): 3069, 2204, 1636, 1465, 1384, 1268. ^1^H-NMR (400 MHz, CDCl_3_ + 0.1 mL CF_3_COOD) *δ* (ppm) 5.80–5.96 (1H, m, H-6), 7.42–7.56 (3H, m, 3x H_AN_), 7.63 (1H, s, H-4), 7.70 (1H, t, *J* = 7.5 Hz, H_AN_), 7.77–7.87 (2H, m, H-9, H_AN_), 7.95 (1H, s, H_AN_), 8.11–8.18 (1H, m, H_AN_), 8.26 (1H, d, *J* = 8.5 Hz, H_AN_), 8.46 (1H, d, *J* = 9.1 Hz, H_AN_), 8.78 (1H, s, H-2). ^13^C{^1^H}-NMR (100 MHz, CDCl_3_ + 0.1 mL CF_3_COOD) *δ* (ppm) 53.5, 77.4, 89.0, 101.4 (d, *J* = 24.8 Hz), 103.5 (d, *J* = 24.5 Hz), 120.0, 120.2, 120.4, 123.8 (d, *J* = 10.1 Hz), 124.3 (d, *J* = 11.1 Hz), 126.0, 126.3, 129.4, 130.2, 130.3, 131.0, 131.3, 131.4, 131.5, 132.0, 133.8, 135.3, 143.0, 149.2 (dd, *J* = 248.1, 11.8 Hz), 150.0 (dd, *J* = 256.5, 19.6 Hz). Calcd for C_25_H_14_F_2_N_4_: C 73.52, H 3.46, N 13.72; found: C 73.63, H 3.49, N 13.58.

General procedure for the synthesis of 4-(aryl)benzo[4,5]imidazo[1,2-*a*]pyrimidine-3-carbonitriles (**6a**–**f**).

To a stirred solution of the appropriate derivatives **5a**–**f** (0.005 mol, 1 equivalent) in DMF (30 mL), MnO_2_ (1.74 g, 0.02 mol, 4 equivalent) was added. The resulting mixture was stirred for 2 h at 130 °C (oil bath temperature) in an open air atmosphere until TLC (EtOAc as eluent) indicated total consumption of starting dihydropyrimidines **5a**–**f**. The reaction mixture was filtered through ceolite, the filtrate was poured into 150 mL of water, and the solid product was collected by filtration to give the expected pure product.

4-(4-(Dimethylamino)phenyl)benzo[4,5]imidazo[1,2-*a*]pyrimidine-3-carbonitrile (**6a**). Orange powder (1.33 g, yield 85%), m.p. > 300 °C. FT-IR (neat) ν_max_ (cm^−1^): 2232, 1604, 1538, 1400, 1372, 1189. ^1^H-NMR (400 MHz, DMSO-*d_6_*) *δ* (ppm) 3.10 (6H, s, -N(CH_3_)_2_), 6.84 (1H, d, *J* = 8.4 Hz, H-6), 7.00 (2H, d, *J* = 8.4 Hz, H-3′, H-5′), 7.21 (1H, t, *J* = 7.9 Hz, H-7), 7.54 (1H, t, *J* = 7.8 Hz, H-8), 7.60 (2H, d, *J* = 8.4 Hz, H-2′, H-6′), 7.90 (1H, d, *J* = 8.2 Hz, H-9), 9.04 (1H, s, H-2). ^13^C{^1^H}-NMR (100 MHz, DMSO-*d*_6_) *δ* (ppm) 39.6 (2C), 94.6, 111.6 (2C), 114.8, 115.1, 116.1, 119.9, 122.2, 126.8, 127.6, 129.6 (2C), 144.6, 150.1, 152.3, 155.3, 156.7. Calcd for C_19_H_15_N_5_: C 72.83, H 4.82, N 22.35; found: C 72.71, H 5.06, N 22.23.

4-(4-Methoxyphenyl)benzo[4,5]imidazo[1,2-*a*]pyrimidine-3-carbonitrile (**6b**). Yellow powder (1.25 g, yield 83%), m.p. 233–235 °C. FT-IR (neat) ν_max_ (cm^−1^): 2230, 1667, 1473, 1091, 1058, 1020. ^1^H-NMR (400 MHz, DMSO-*d_6_*) *δ* (ppm) 3.95 (3H, s, OCH_3_), 6.54 (1H, d, *J* = 8.5 Hz, H-6), 7.20 (1H, t, *J* = 7.8 Hz, H-7), 7.34 (2H, d, *J* = 8.7 Hz, H-3′, H-5′), 7.55 (1H, t, *J* = 7.7 Hz, H-8), 7.77 (2H, d, *J* = 8.5 Hz, H-2′, H-6′), 7.92 (1H, d, *J* = 8.1 Hz, H-9), 9.11 (1H, s, H-2). ^13^C{^1^H}-NMR (100 MHz, DMSO-*d*_6_) *δ* (ppm) 55.6, 95.0, 114.8, 115.1 (2C), 115.6, 120.0, 121.1, 122.5, 127.0, 127.4, 130.1 (2C), 144.6, 149.8, 155.1, 155.9, 161.9. Calcd for C_18_H_12_N_4_O: C 71.99, H 4.03, N 18.66; found: C 71.80, H 3.91, N 18.70.

4-(Anthracen-9-yl)benzo[4,5]imidazo[1,2-*a*]pyrimidine-3-carbonitrile (**6c**). Yellow powder (1.66 g, yield 90%), m.p. > 300 °C. FT-IR (neat) ν_max_ (cm^−1^): 3051, 2227, 1621, 1483, 1446, 1350. ^1^H-NMR (400 MHz, DMSO-*d_6_*) *δ* (ppm) 5.29 (1H, d, *J* = 8.4 Hz, H-6), 6.72–6.80 (1H, m, H-7), 7.39 (1H, t, *J* = 7.3 Hz, H-8), 7.46–7.53 (2H, m, 2xH_AN_), 7.62–7.73 (4H, m, 4xH_AN_), 7.92 (1H, d, *J* = 8.3 Hz, H-9), 8.38 (2H, d, *J* = 8.5 Hz, 2xH_AN_), 9.20 (1H, s, H_AN_), 9.37 (1H, s, H-2). ^13^C{^1^H}-NMR (100 MHz, DMSO-*d*_6_) *δ* (ppm) 97.2, 113.5, 115.0, 120.1, 121.0, 123.0, 123.7 (2C), 126.5 (2C), 126.6, 127.0, 128.6 (2C), 128.8 (2C), 129.3 (2C), 130.6 (2C), 132.1, 144.7, 149.9, 153.0, 155.4. Calcd for C_25_H_14_N_4_: C 81.06, H 3.81, N 15.13; found: C 80.94, H 3.58, N 15.12.

4-(4-(Dimethylamino)phenyl)-7,8-difluorobenzo[4,5]imidazo[1,2-*a*]pyrimidine-3-carbonitrile (**6d**). Orange powder (1.41 g, yield 81%), m.p. 284–286 °C. FT-IR (neat) ν_max_ (cm^−1^): 3082, 2225, 1603, 1438, 1398, 1377. ^1^H-NMR (400 MHz, DMSO-*d_6_*) *δ* (ppm) 3.11 (6H, s, -N(CH_3_)_2_), 6.63 (1H, t, *J* = 9.3 Hz, H-6), 7.02 (2H, d, *J* = 8.4 Hz, H-3′, H-5′), 7.61 (2H, d, *J* = 8.3 Hz, H-2′, H-6′), 8.02 (1H, t, *J* = 9.2 Hz, H-9), 9.07 (1H, s, H-2). ^13^C{^1^H}-NMR (100 MHz, DMSO-*d*_6_) *δ* (ppm) 95.0, 103.1 (d, *J* = 24.4 Hz), 107.1 (d, *J* = 19.9 Hz), 111.5 (2C), 113.7, 115.4, 122.7 (d, *J* = 10.7 Hz), 129.5 (2C), 140.8 (d, *J* = 11.6 Hz), 145.3 (dd, *J* = 241.7, 15.4 Hz), 149.1 (dd, *J* = 245.8, 14.8 Hz), 151.2, 152.5, 155.4, 156.1. Calcd for C_19_H_13_F_2_N_4_: C 65.32, H 3.75, N 20.05; found: C 65.53, H 3.89, N 19.92.

7,8-Difluoro-4-(4-methoxyphenyl)benzo[4,5]imidazo[1,2-*a*]pyrimidine-3-carbonitrile (**6e**). Beige powder (1.34 g, yield 80%), m.p. 246–248 °C. FT-IR (neat) ν_max_ (cm^−1^): 3046, 2229, 1595, 1530, 1490, 1101. ^1^H NMR (400 MHz, DMSO-*d_6_*) *δ* (ppm) 3.96 (3H, s, OCH_3_), 6.31 (1H, dd, *J* = 10.8, 7.3 Hz, H-6), 7.37 (2H, d, *J* = 8.3 Hz, H-3′, H-5′), 7.77 (2H, d, J = 8.3 Hz, H-2′, H-6′), 8.05 (1H, dd, *J* = 10.8, 7.5 Hz, H-9), 9.15 (1H, s, H-2). ^13^C{^1^H}-NMR (100 MHz, DMSO-*d*_6_) *δ* (ppm) 55.6, 95.6, 103.0 (d, *J* = 24.4 Hz), 107.5 (d, *J* = 19.8 Hz), 115.2, 115.3 (2C), 120.3, 122.7 (d, *J* = 10.9 Hz), 130.2 (2C), 140.9 (d, *J* = 11.8 Hz), 145.7 (dd, *J* = 242.2, 15.6 Hz), 149.3 (dd, *J* = 245.8, 15.0 Hz), 151.0, 155.5, 155.6, 162.2. Calcd for C_18_H_10_F_2_N_4_O: C 64.29, H 3.00, N 16.66; found: C 64.35, H 3.19, N 16.52.

4-(Anthracen-9-yl)-7,8-difluorobenzo[4,5]imidazo[1,2-*a*]pyrimidine-3-carbonitrile (**6f**). Yellow powder (1.71 g, yield 84%), m.p. > 300 °C. FT-IR (neat) ν_max_ (cm^−1^): 3088, 2230, 1594, 1505, 1465, 1074. ^1^H NMR (400 MHz, DMSO-*d_6_*) *δ* (ppm) 5.00 (1H, t, *J* = 8.9 Hz, H-6), 7.52 (2H, t, *J* = 7.7 Hz, 2xH_AN_), 7.67 (2H, t, *J* = 7.5 Hz, 2xH_AN_), 7.75 (2H, d, *J* = 8.8 Hz, 2xH_AN_), 8.07 (1H, t, *J* = 9.3 Hz, H-9), 8.38 (2H, d, *J* = 8.6 Hz, 2xH_AN_), 9.23 (1H, s, H-2), 9.41 (1H, s, H_AN_). ^13^C{^1^H}-NMR (100 MHz, DMSO-*d*_6_) *δ* (ppm) 98.0, 101.5 (d, *J* = 24.5 Hz), 107.9 (d, *J* = 19.9 Hz), 114.7, 119.9, 121.9 (d, *J* = 10.8 Hz), 123.6 (2C), 126.6 (2C), 128.7 (2C), 129.1 (2C), 129.3 (2C), 130.5 (2C), 132.5, 141.1 (d, *J* = 11.8 Hz), 145.9 (dd, *J* = 243.2, 15.6 Hz), 149.3 (dd, *J* = 246.6, 14.9 Hz), 151.3, 152.6, 155.7. Calcd for C_25_H_12_F_2_N_4_: C 73.89, H 2.98, N 13.79; found: C 74.05, H 2.85, N 13.62.

### 3.2. Crystallography Experiment

The XRD analyses were carried out using equipment of the Center for Joint Use “Spectroscopy and Analysis of Organic Compounds” at the Postovsky Institute of Organic Synthesis of the Russian Academy of Sciences (Ural Branch). The experiment was carried out on a standard procedure (MoK_α_-irradiation, graphite monochromator, ω-scans with 1⁰ step at T = 295(2) K) on an automated X-ray diffractometer Xcalibur 3 with a CCD detector. Empirical absorption correction was applied. The solution and refinement of the structures were accomplished using the Olex program package [65]. The structures were solved by the method of the intrinsic phases in the ShelXT program and refined by the ShelXL by full-matrix least-squares method for non-hydrogen atoms [66]. The H atoms were placed in the calculated positions and refined in isotropic approximation.

Crystal Data for C_51_H_30_Cl_2_N_8_ (M = 825.73 g/mol): triclinic, space group P-1, a = 8.5135(4) Å, b = 10.4646(5) Å, c = 22.9053(12) Å, α = 88.784(4)°, β = 85.741(4)°, γ = 82.301(4)°, V = 2016.53(17) Å^3^, Z = 2, T = 295(2) K, μ(MoK_α_) = 0.210 mm^−1^, Dcalc = 1.360 g/cm^3^, 20,634 reflections measured (7.384° ≤ 2Θ ≤ 60.982°), 10,876 unique (R_int_ = 0.0577, R_sigma_ = 0.0845), which were used in all calculations. The final R_1_ = 0.0767, wR_2_ = 0.1916 (I > 2σ(I)) and R_1_ = 0.1463, wR_2_ = 0.2616 (all data). Largest peak/hole difference is 0.34/−0.35.

The XRD data were deposited in the Cambridge Structural Database with the number CCDC 2215090. This data can be requested free of charge via www.ccdc.cam.ac.uk (accessed on 17 November 2022).

### 3.3. DFT Calculations

The quantum chemical calculations were performed at the B3LYP/6-31G*//PM6 level of theory using the Gaussian-09 program package (M. J. Frisch, G. W. Trucks, H. B. Schlegel, G. E. Scuseria, M. A. Robb, J. R. Cheeseman, G. Scalmani, V. Barone, B. Mennucci, G. A. Petersson, H. Nakatsuji, M. Caricato, X. Li, H. P. Hratchian, A. F. Izmaylov, J. Bloino, G. Zheng, J. L. Sonnenberg, M. Had DJF. Gaussian 09, Revision C.01. Wallingford, CT 2010). No symmetry restrictions were applied during the geometry optimization procedure. The solvent effects were taken into account using the SMD (solvation model based on density) continuum solvation model suggested by Truhlar et al. [67] for THF. The Hessian matrices were calculated for all optimized model structures to prove the location of correct minima on the potential energy surface (no imaginary frequencies were found in all cases). The Chemcraft program http://www.chemcraftprog.com/ (accessed on 17 November 2022) was used for visualization. The hole-electron analysis was carried out in Multiwfn program (version 3.7) [61]. The Cartesian atomic coordinates for all optimized equilibrium model structures are presented in the attached xyz-files.

## 4. Conclusions

In summary, we have designed and synthesized a series of novel 4-(aryl)-benzo [4,5]imidazo[1,2-*a*]pyrimidine-3-carbonitriles by successive transformations, including the preparation of benzimidazole-2-arylimines, the Povarov reaction, and the oxidation of dihydrobenzo [4,5]imidazo[1,2-*a*]pyrimidine-3-carbonitriles. Based on the literature data and X-ray diffraction analysis, it was found that during the Povarov reaction, [1,3] sigmatropic rearrangement occurred. The structure of the synthesized compounds is unambiguously confirmed by the set of spectral data. For the derivatives **6a**–**f**, the ordinary photophysical properties such as absorption, emission, lifetime, and QY in solution, as well as emission and QY in powder, were studied. For the chromophore **6c**, Aggregation-Induced Emission (AIE) has been illustrated using different water fractions (fw) in THF. Finally, the mechanofluorochromic properties of derivatives **6c** and **6f** were investigated, and the response to mechanical stimulation with changing emission maxima or/and intensity was recorded. The significant photophysical properties and availability of 4-(aryl)-benzo[4,5]imidazo[1,2-a]pyrimidine-3-carbonitriles pave the way for future applications in biology, medicine, ecology, and photonics.

## Figures and Tables

**Table 1:** Optimization of the reaction conditions for dihydropyrimidin **5a** ^1^.

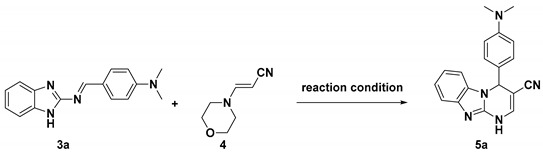					
No.	Solvent ^2^	Activating Agent (Catalysts)	X, Equiv	Reaction Condition ^3^	Yield, % ^4^
entry 1	EtOH	BF_3_∙Et_2_O	0.5	reflux, 5 h	35
entry 2	*i*-PrOH	BF_3_∙Et_2_O	0.5	reflux, 5 h	46
entry 3	*n*-BuOH	BF_3_∙Et_2_O	0.5	reflux, 5 h	50
entry 4	Toluene	BF_3_∙Et_2_O	0.5	reflux, 5 h	-
entry 5	*n*-BuOH	BF_3_∙Et_2_O	0.5	reflux, 6 h	51
entry 6	AcOH	-	-	reflux, 5 h	-
entry 7	*n*-BuOH	Et_3_N	0.5	reflux, 5 h	-
entry 8	*n*-BuOH	BF_3_∙Et_2_O	1.0	reflux, 5 h	63
entry 9	*n*-BuOH	BF_3_∙Et_2_O	1.5	reflux, 5 h	74
entry 10	*n*-BuOH	BF_3_∙Et_2_O	2.0	reflux, 5 h	76

^1^ Reaction conditions: **3a** (0.10 mmol) and **4** (0.10 mmol); ^2^ amount of solvent—5 mL; ^3^ conventional heating with an oil bath; and ^4^ isolated yield.

**Table 2:** Optimization of the oxidation reactions for dihydropyrimidin **5a** ^1^.

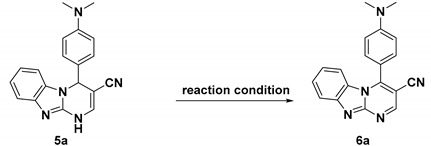						
No.	Solvent ^2^	Oxidant ^3^	X, Equiv ^3^	Reaction Condition ^4^	Conversion, % ^5^	Yield, % ^6^
entry 1	DMF	-	-	Heating 100 °C, 1 h	10	5
entry 2	DMF	-	-	Heating 140 °C, 1 h	15	6
entry 3	DMF	-	-	Heating 140 °C, 4 h	30	17
entry 4	DMF	-	-	Heating 140 °C, 12 h	40	30
entry 5	DMF	MnO_2_	1.0	Heating 120 °C, 6 h	100	83
entry 6	DMF	MnO_2_	2.0	Heating 120 °C, 4 h	100	84
entry 7	DMF	MnO_2_	3.0	Heating 120 °C, 1.5 h	100	86
entry 8	DMF	MnO_2_	4.0	Heating 120 °C, 1.0 h	100	85

^1^ Reaction conditions: **5a** (0.10 mmol); ^2^ amount of solvent—5 mL; ^3^ X equivalent of oxidant; ^4^ conventional heating with an oil bath; ^5^ in accordance with TLC; and ^6^ isolated yield.

**Table 3:** Data of photophysical properties of fluorophores (**6a**–**f**) (10^−5^ M) in THF solvent.

No.	λ_abs_^max^, nm (ε_M_, 10^4^ M^−1^ cm^−1^) ^1^	λ_em_^max^, nm ^2^	Stokes Shift, nm/cm^−1^	τ_av_, ns ^3^	*Φ_f_*, % ^4^
**6a**	268 (4.8) 413 (0.74)	554	141/6162	2.43	7.5
**6b**	271 (3.53) 312 (0.52) 320 (0.52) 387 (0.11)	540	153/7321	5.12	1.9
**6c**	254 (14.5) 327 (0.45) 342 (0.45) 371 (0.49) 391 (0.48)	550	159/7394	8.78	<0.1
**6d**	264 (4.12) 421 (0.76)	567	146/6116	1.59	2.9
**6e**	266 (5.65) 280 (5.30) 377 (0.14)	520	143/7294	6.24	1.1
**6f**	255 (13.21) 323 (0.43) 336 (0.47) 372 (0.48) 392 (0.45)	524	132/6426	2.34	4.8

^1^ Absorption spectra were measured at r.t. in THF in range from 230 to 500 nm; ^2^ emission spectra were measured at r.t. in THF; ^3^ weighted average decay time τ_av_ = Σ (τ_i_ × α_i_) in THF (LED 370 nm); and ^4^ absolute quantum yields were measured using the Integrating Sphere of the Horiba FluoroMax-4 at r.t. in THF.

**Table 4:** Optical properties of the compounds **6a**–**f** in the solid state and in PVA film.

No.	In PVA Film		In Powder	
	λ_em_^max^, nm	Φ*_f_*, (%) ^1^	λ_em_^max^, nm	Φ*_f_*, (%) ^1^
**6a**	546	4.8	572	20.5
**6b**	545	49.6	517	17.8
**6c**	546	25.6	511	3.9
**6d**	545	13.9	626	8.3
**6e**	542	12.0	509	19.3
**6f**	540	34.5	525	3.4

^1^ Absolute quantum yields were measured using the Integrating Sphere of the Horiba FluoroMax-4 at r.t. in film/powder form.

**Table 5:** HOMO/LUMO based on the functionality B3LYP/6-311G* in the THF phase.

Compound	HOMO, eV	LUMO, eV	ΔE, eV
**6a**	−5.43	−2.04	3.39
**6b**	−5.96	−2.13	3.83
**6c**	−5.56	−2.16	3.40
**6d**	−5.38	−2.14	3.24
**6e**	−6.04	−2.23	3.81
**6f**	−5.59	−2.21	3.38

**Table 6:** Calculated dipole moments for model structures in ground and excited multiplicity states and estimated indexes related to hole-electron distribution (CT-indexes).

Compound	Dipole Moment in Ground Multiplicity State (Debye)	Dipole Moment in Excited Multiplicity State (Debye)	D(Å)	S_r_ (a.u.)	t (Å)
**6b**	3.1676	4.3108	0.978	0.62822	−0.326
**6e**	4.0313	1.9340	0.927	0.62287	−0.550
**6a**	3.1463	9.2304	3.722	0.50976	0.617
**6d**	6.5432	13.7974	3.832	0.50975	0.678
**6c**	3.8859	1.8867	4.406	0.26547	2.599
**6f**	1.7563	3.5274	4.523	0.24338	2.707

## Data Availability

Data are contained within the article.

## Supplementary Materials

The following supporting information can be downloaded at: https://www.mdpi.com/article/10.3390/molecules27228029/s1, Table S1: Fluorescence lifetime of probes **6a**–**f** (C = 2 × 10^−6^ M) in THF; Table S2: Orientation polarizability for solvents (Δf), absorption and fluorescence emission maxima (λ_abs_, λ_em_, nm), and Stokes shift (nm, cm^−1^) of **6a** in different solvents; Table S3: Orientation polarizability for solvents (Δf), absorption and fluorescence emission maxima (λ_abs_, λ_em_, nm), and Stokes shift (nm, cm^−1^) of **6b** in different solvents; Table S4: Orientation polarizability for solvents (Δf), absorption and fluorescence emission maxima (λ_abs_, λ_em_, nm), and Stokes shift (nm, cm^−1^) of **6c** in different solvents; Table S5: Orientation polarizability for solvents (Δf), absorption and fluorescence emission maxima (λ_abs_, λ_em_, nm) and Stokes shift (nm, cm^−1^) of **6d** in different solvents; Table S6: Orientation polarizability for solvents (Δf), absorption and fluorescence emission maxima (λ_abs_, λ_em_, nm) and Stokes shift (nm, cm^−1^) of **6e** in different solvents; Table S7: Orientation polarizability for solvents (Δf), absorption and fluorescence emission maxima (λ_abs_, λ_em_, nm) and Stokes shift (nm, cm^−1^) of **6f** in different solvents; Table S8: Fluorescence lifetime of probe **6c** (C = 2 × 10^−6^ M) in THF/water mixtures with water fractions 0/60 (vol%); Table S9: Mechanochromic properties of probes **6a**–**f**; Table S10: Crystal data and structure refinement for **6c**; Table S11: Fractional Atomic Coordinates (×10^4^) and Equivalent Isotropic Displacement Parameters (Å2 × 10^3^) for **6c**; Table S12: Anisotropic Displacement Parameters (Å2 × 10^3^) for **6c**; Table S13: Bond Lengths for **6c**; Table S14: Bond Angles for **6c**; Table S15: Torsion Angles for **6c**; Table S16: Hydrogen Atom Coordinates (Å × 10^4^) and Isotropic Displacement Parameters (Å2 × 10^3^) Table S17: for **6c**; Atomic Occupancy for 6c; Figure S1: Solvent effect of **6c** and **6f**; Figure S2: UV-Vis absorption spectra of **6c** in THF/water mixtures with water fractions 0/60% (**A**). Time-resolved emission decay curves of **6c** in THF/water mixtures with water fractions 0/60% (**B**); Figure S3: Solvent effect for **6f** in THF/water; Figures S4–S20: ^1^H- and ^13^C-NMR spectra of compounds **3a**,**c**, **3d**–**f**, **5a**–**f**, and **6a**–**f**; Figures S21–S29 IR spectra of compounds **3a**,**c**, **3d**–**f**, **5a**–**f**, and **6a**–**f**.

## Sample Availability

Samples of the compounds **3a**–**c**, **4**, **5a**–**f**, and **6a**–**f** are available from the authors.

## References

[1] Alqarni S., Cooper L., Galvan Achi J., Bott R., Sali V.K., Brown A., Santarsiero B.D., Krunic A., Manicassamy B., Peet N.P. (2022). Synthesis, Optimization, and Structure–Activity Relationships of Imidazo[1,2-*a*]Pyrimidines as Inhibitors of Group 2 Influenza A Viruses. J. Med. Chem..

[2] Massari S., Bertagnin C., Pismataro M.C., Donnadio A., Nannetti G., Felicetti T., Di Bona S., Nizi M.G., Tensi L., Manfroni G. (2021). Synthesis and Characterization of 1,2,4-Triazolo[1,5-*a*]Pyrimidine-2-Carboxamide-Based Compounds Targeting the PA-PB1 Interface of Influenza A Virus Polymerase. Eur. J. Med. Chem..

[3] Perlíková P., Hocek M. (2017). Pyrrolo[2,3-*d*]Pyrimidine (7-Deazapurine) as a Privileged Scaffold in Design of Antitumor and Antiviral Nucleosides. Med. Res. Rev..

[4] Jung E., Soto-Acosta R., Geraghty R.J., Chen L. (2022). Zika Virus Inhibitors Based on a 1,3-Disubstituted 1H-Pyrazolo[3,4-*d*]Pyrimidine-Amine Scaffold. Molecules.

[5] Romagnoli R., Oliva P., Prencipe F., Manfredini S., Budassi F., Brancale A., Ferla S., Hamel E., Corallo D., Aveic S. (2022). Design, Synthesis and Biological Investigation of 2-Anilino Triazolopyrimidines as Tubulin Polymerization Inhibitors with Anticancer Activities. Pharmaceuticals.

[6] Yu G.-X., Hu Y., Zhang W.-X., Tian X.-Y., Zhang S.-Y., Zhang Y., Yuan S., Song J. (2022). Design, Synthesis and Biological Evaluation of [1,2,4]Triazolo[1,5-*a*]Pyrimidine Indole Derivatives against Gastric Cancer Cells MGC-803 via the Suppression of ERK Signaling Pathway. Molecules.

[7] Shi X., Quan Y., Wang Y., Wang Y., Li Y. (2021). Design, Synthesis, and Biological Evaluation of 2,6,7-Substituted Pyrrolo[2,3-*d*]Pyrimidines as Cyclin Dependent Kinase Inhibitor in Pancreatic Cancer Cells. Bioorganic Med. Chem. Lett..

[8] Ding R., Wang X., Fu J., Chang Y., Li Y., Liu Y., Liu Y., Ma J., Hu J. (2022). Design, Synthesis and Antibacterial Activity of Novel Pleuromutilin Derivatives with Thieno[2,3-*d*]Pyrimidine Substitution. Eur. J. Med. Chem..

[9] Sutherland H.S., Choi P.J., Lu G.-L., Giddens A.C., Tong A.S.T., Franzblau S.G., Cooper C.B., Palmer B.D., Denny W.A. (2022). Synthesis and Structure–Activity Relationships for the Anti-Mycobacterial Activity of 3-Phenyl-N-(Pyridin-2-Ylmethyl)Pyrazolo[1,5-*a*]Pyrimidin-7-Amines. Pharmaceuticals.

[10] Shen J., Deng X., Sun R., Tavallaie M.S., Wang J., Cai Q., Lam C., Lei S., Fu L., Jiang F. (2020). Structural Optimization of Pyrazolo[1,5-*a*]Pyrimidine Derivatives as Potent and Highly Selective DPP-4 Inhibitors. Eur. J. Med. Chem..

[11] Peytam F., Adib M., Shourgeshty R., Firoozpour L., Rahmanian-Jazi M., Jahani M., Moghimi S., Divsalar K., Faramarzi M.A., Mojtabavi S. (2020). An Efficient and Targeted Synthetic Approach towards New Highly Substituted 6-Amino-Pyrazolo[1,5-*a*]Pyrimidines with α-Glucosidase Inhibitory Activity. Sci. Rep..

[12] Tigreros A., Macías M., Portilla J. (2022). Expeditious Ethanol Quantification Present in Hydrocarbons and Distilled Spirits: Extending Photophysical Usages of the Pyrazolo[1,5-*a*]Pyrimidines. Dye. Pigment..

[13] Fedotov V.V., Ulomsky E.N., Belskaya N.P., Eltyshev A.K., Savateev K.V., Voinkov E.K., Lyapustin D.N., Rusinov V.L. (2021). Benzimidazoazapurines: Design, Synthesis, and Photophysical Study. J. Org. Chem..

[14] Tigreros A., Castillo J.C., Portilla J. (2020). Cyanide Chemosensors Based on 3-Dicyanovinylpyrazolo[1,5-a]Pyrimidines: Effects of Peripheral 4-Anisyl Group Substitution on the Photophysical Properties. Talanta.

[15] Zhang M., Cheng R., Lan J., Zhang H., Yan L., Pu X., Huang Z., Wu D., You J. (2019). Oxidative C-H/C-H Cross-Coupling of [1,2,4]Triazolo[1,5-a]Pyrimidines with Indoles and Pyrroles: Discovering Excited-State Intramolecular Proton Transfer (ESIPT) Fluorophores. Org. Lett..

[16] Ooyama Y., Uenaka K., Ohshita J. (2015). Development of a Functionally Separated D–π-A Fluorescent Dye with a Pyrazyl Group as an Electron-Accepting Group for Dye-Sensitized Solar Cells. Org. Chem. Front..

[17] Dinastiya E.M., Verbitskiy E.V., Gadirov R.M., Samsonova L.G., Degtyarenko K.M., Grigoryev D.V., Kurtcevich A.E., Solodova T.A., Tel’minov E.N., Rusinov G.L. (2021). Investigation of 4,6-Di(Hetero)Aryl-Substituted Pyrimidines as Emitters for Non-Doped OLED and Laser Dyes. J. Photochem. Photobiol. A Chem..

[18] Fecková M., le Poul P., Bureš F., Robin-le Guen F., Achelle S. (2020). Nonlinear Optical Properties of Pyrimidine Chromophores. Dye. Pigment..

[19] Debnath S., Parveen S., Pradhan P., Das I., Das T. (2022). Benzo[4,5]Imidazo[1,2-*a*]Pyridines and Benzo[4,5]Imidazo[1,2-*a*]Pyrimidines: Recent Advancements in Synthesis of Two Diversely Important Heterocyclic Motifs and Their Derivatives. New J. Chem..

[20] Fedotov V.V., Rusinov V.L., Ulomsky E.N., Mukhin E.M., Gorbunov E.B., Chupakhin O.N. (2021). Pyrimido[1,2-*a*]Benzimidazoles: Synthesis and Perspective of Their Pharmacological Use. Chem. Heterocycl. Comp..

[21] Fedotov V.V., Ulomskiy E.N., Gorbunov E.B., Eltsov O.S., Voinkov E.K., Savateev K.V., Drokin R.A., Kotovskaya S.K., Rusinov V.L. (2017). 3-Nitropyrimido[1,2-*a*]Benzimidazol-4-Ones: Synthesis and Study of Alkylation Reaction. Chem. Heterocycl. Comp..

[22] Manna S.K., Das T., Samanta S. (2019). Polycyclic Benzimidazole: Synthesis and Photophysical Properties. ChemistrySelect.

[23] Vil’ V.A., Grishin S.S., Baberkina E.P., Alekseenko A.L., Glinushkin A.P., Kovalenko A.E., Terent’ev A.O. (2022). Electrochemical Synthesis of Tetrahydroquinolines from Imines and Cyclic Ethers *via* Oxidation/Aza-Diels-Alder Cycloaddition. Adv. Synth. Catal..

[24] Steinke T., Wonner P., Gauld R.M., Heinrich S., Huber S.M. (2022). Catalytic Activation of Imines by Chalcogen Bond Donors in a Povarov [4+2] Cycloaddition Reaction. Chem. A Eur. J..

[25] Clerigué J., Ramos M.T., Menéndez J.C. (2021). Mechanochemical Aza-Vinylogous Povarov Reactions for the Synthesis of Highly Functionalized 1,2,3,4-Tetrahydroquinolines and 1,2,3,4-Tetrahydro-1,5-Naphthyridines. Molecules.

[26] Cores Á., Clerigué J., Orocio-Rodríguez E., Menéndez J.C. (2022). Multicomponent Reactions for the Synthesis of Active Pharmaceutical Ingredients. Pharmaceuticals.

[27] Jiménez-Aberásturi X., Palacios F., de los Santos J.M. (2022). Sc(OTf)_3_-Mediated [4 + 2] Annulations of *N*-Carbonyl Aryldiazenes with Cyclopentadiene to Construct Cinnoline Derivatives: Azo-Povarov Reaction. J. Org. Chem..

[28] Ghashghaei O., Masdeu C., Alonso C., Palacios F., Lavilla R. (2018). Recent Advances of the Povarov Reaction in Medicinal Chemistry. Drug Discov. Today Technol..

[29] Sun Y., Lei Z., Ma H. (2022). Twisted Aggregation-Induced Emission Luminogens (AIEgens) Contribute to Mechanochromism Materials: A Review. J. Mater. Chem. C.

[30] Cooper M.W., Zhang X., Zhang Y., Ashokan A., Fuentes-Hernandez C., Salman S., Kippelen B., Barlow S., Marder S.R. (2022). Delayed Luminescence in 2-Methyl-5-(Penta(9-Carbazolyl)Phenyl)-1,3,4-Oxadiazole Derivatives. J. Phys. Chem. A.

[31] Gong X., Xiang Y., Ning W., Zhan L., Gong S., Xie G., Yang C. (2022). A Heterocycle Fusing Strategy for Simple Construction of Efficient Solution-Processable Pure-Red Thermally Activated Delayed Fluorescence Emitters. J. Mater. Chem. C.

[32] Wang X., Li Y., Wu Y., Qin K., Xu D., Wang D., Ma H., Ning S., Wu Z. (2022). A 2-Phenylfuro[2,3-*b*]Quinoxaline-Triphenylamine-Based Emitter: Photophysical Properties and Application in TADF-Sensitized Fluorescence OLEDs. New J. Chem..

[33] Hojo R., Mayder D.M., Hudson Z.M. (2021). Donor–Acceptor Materials Exhibiting Deep Blue Emission and Thermally Activated Delayed Fluorescence with Tris(Triazolo)Triazine. J. Mater. Chem. C.

[34] Rodella F., Saxena R., Bagnich S., Banevičius D., Kreiza G., Athanasopoulos S., Juršėnas S., Kazlauskas K., Köhler A., Strohriegl P. (2021). Low Efficiency Roll-off Blue TADF OLEDs Employing a Novel Acridine–Pyrimidine Based High Triplet Energy Host. J. Mater. Chem. C.

[35] Devesing Girase J., Rani Nayak S., Tagare J., Shahnawaz, Ram Nagar M., Jou J.-H., Vaidyanathan S. (2022). Solution-Processed Deep-Blue (Y∼0.06) Fluorophores Based on Triphenylamine-Imidazole (Donor-Acceptor) for OLEDs: Computational and Experimental Exploration. J. Inf. Disp..

[36] Anupriya, Justin Thomas K.R., Nagar M.R., Jou J.-H. (2022). Effect of Cyano Substituent on the Functional Properties of Blue Emitting Imidazo[1,2-*a*]Pyridine Derivatives. Dye. Pigment..

[37] Ohsawa T., Sasabe H., Watanabe T., Nakao K., Komatsu R., Hayashi Y., Hayasaka Y., Kido J. (2019). A Series of Imidazo[1,2-f]Phenanthridine-Based Sky-Blue TADF Emitters Realizing EQE of over 20%. Adv. Opt. Mater..

[38] Wang Z.-Y., Zhao J.-W., Li P., Feng T., Wang W.-J., Tao S.-L., Tong Q.-X. (2018). Novel Phenanthroimidazole-Based Blue AIEgens: Reversible Mechanochromism, Bipolar Transporting Properties, and Electroluminescence. New J. Chem..

[39] Godumala M., Choi S., Cho M.J., Choi D.H. (2016). Thermally Activated Delayed Fluorescence Blue Dopants and Hosts: From the Design Strategy to Organic Light-Emitting Diode Applications. J. Mater. Chem. C.

[40] Kothavale S., Lee K.H., Lee J.Y. (2020). Molecular Design Strategy of Thermally Activated Delayed Fluorescent Emitters Using CN-Substituted Imidazopyrazine as a New Electron-Accepting Unit. Chem. Asian J..

[41] Anupriya, Thomas K.R.J., Nagar M.R., Shahnawaz, Jou J.-H. (2022). Phenanthroimidazole Substituted Imidazo[1,2-a]Pyridine Derivatives for Deep-Blue Electroluminescence with CIEy ∼ 0.08. J. Photochem. Photobiol. A Chem..

[42] Taniya O.S., Fedotov V.V., Novikov A.S., Sadieva L.K., Krinochkin A.P., Kovalev I.S., Kopchuk D.S., Zyryanov G.V., Liu Y., Ulomsky E.N. (2022). Abnormal Push-Pull Benzo[4,5]Imidazo[1,2-*a*][1,2,3]Triazolo[4,5-*e*]Pyrimidine Fluorophores in Planarized Intramolecular Charge Transfer (PLICT) State: Synthesis, Photophysical Studies and Theoretical Calculations. Dye. Pigment..

[43] Barik S., Skene W.G. (2013). Turning-on the Quenched Fluorescence of Azomethines through Structural Modifications: Turning-on the Quenched Fluorescence of Azomethines. Eur. J. Org. Chem..

[44] Barluenga J., Aznar F., Valdes C., Cabal M.P. (1993). Stereoselective Synthesis of 4-Piperidone and 4-Aminotetrahydropyridine Derivatives by the Imino Diels-Alder Reaction of 2-Amino-1,3-Butadienes. J. Org. Chem..

[45] Montalvo-González R., Ariza-Castolo A. (2003). Molecular Structure of Di-Aryl-Aldimines by Multinuclear Magnetic Resonance and X-Ray Diffraction. J. Mol. Struct..

[46] Nowicka A., Liszkiewicz H., Nawrocka W., Wietrzyk J., Kempińska K., Dryś A. (2014). Synthesis and Antiproliferative Activity in Vitro of New 2-Aminobenzimidazole Derivatives. Reaction of 2-Arylideneaminobenzimidazole with Selected Nitriles Containing Active Methylene Group. Open Chem..

[47] Bogolubsky A.V., Moroz Y.S., Mykhailiuk P.K., Panov D.M., Pipko S.E., Konovets A.I., Tolmachev A. (2014). A One-Pot Parallel Reductive Amination of Aldehydes with Heteroaromatic Amines. ACS Comb. Sci..

[48] Yu J., Hu P., Zhou T., Xu Y. (2011). Synthesis of Benzimidazole Thiazolinone Derivatives under Microwave Irradiation. J. Chem. Res..

[49] Chen C.-H., Yellol G.S., Lin P.-T., Sun C.-M. (2011). Base-Catalyzed Povarov Reaction: An Unusual[1,3]Sigmatropic Rearrangement to Dihydropyrimidobenzimidazoles. Org. Lett..

[50] Hsiao Y.-S., Narhe B.D., Chang Y.-S., Sun C.-M. (2013). One-Pot, Two-Step Synthesis of Imidazo[1,2-*a*]Benzimidazoles via A Multicomponent [4 + 1] Cycloaddition Reaction. ACS Comb. Sci..

[51] Shah A.P., Hura N., Kishore Babu N., Roy N., Krishna Rao V., Paul A., Kumar Roy P., Singh S., Guchhait S.K. (2022). A Core-Linker-Polyamine (CLP) Strategy Enables Rapid Discovery of Antileishmanial Aminoalkylquinolinecarboxamides That Target Oxidative Stress Mechanism. ChemMedChem.

[52] Kuznetsova E.A., Smolobochkin A.V., Rizbayeva T.S., Gazizov A.S., Voronina J.K., Lodochnikova O.A., Gerasimova D.P., Dobrynin A.B., Syakaev V.V., Shurpik D.N. (2022). Diastereoselective Intramolecular Cyclization/Povarov Reaction Cascade for the One-Pot Synthesis of Polycyclic Quinolines. Org. Biomol. Chem..

[53] Vicente-García E., Catti F., Ramón R., Lavilla R. (2010). Unsaturated Lactams: New Inputs for Povarov-Type Multicomponent Reactions. Org. Lett..

[54] Thakur D., Nagar M.R., Tomar A., Dubey D.K., Kumar S., Swayamprabha S.S., Banik S., Jou J.-H., Ghosh S. (2021). Through Positional Isomerism: Impact of Molecular Composition on Enhanced Triplet Harvest for Solution-Processed OLED Efficiency Improvement. ACS Appl. Electron. Mater..

[55] Anupriya, Thomas K.R.J., Nagar M.R., Shahnawaz, Jou J.-H. (2021). Imidazo[1,2-a]Pyridine Based Deep-Blue Emitter: Effect of Donor on the Optoelectronic Properties. J. Mater. Sci. Mater. Electron..

[56] Leung C.W.T., Hong Y., Chen S., Zhao E., Lam J.W.Y., Tang B.Z. (2013). A Photostable AIE Luminogen for Specific Mitochondrial Imaging and Tracking. J. Am. Chem. Soc..

[57] Hong Y., Lam J.W.Y., Tang B.Z. (2011). Aggregation-Induced Emission. Chem. Soc. Rev..

[58] Suman G.R., Pandey M., Chakravarthy A.S.J. (2021). Review on New Horizons of Aggregation Induced Emission: From Design to Development. Mater. Chem. Front..

[59] Kwon M.S., Gierschner J., Yoon S.-J., Park S.Y. (2012). Unique Piezochromic Fluorescence Behavior of Dicyanodistyrylbenzene Based Donor-Acceptor-Donor Triad: Mechanically Controlled Photo-Induced Electron Transfer (eT) in Molecular Assemblies. Adv. Mater..

[60] Le Bahers T., Adamo C., Ciofini I. (2011). A Qualitative Index of Spatial Extent in Charge-Transfer Excitations. J. Chem. Theory Comput..

[61] Lu T., Chen F. (2012). Multiwfn: A Multifunctional Wavefunction Analyzer. J. Comput. Chem..

[62] Peach M.J.G., Benfield P., Helgaker T., Tozer D.J. (2008). Excitation Energies in Density Functional Theory: An Evaluation and a Diagnostic Test. J. Chem. Phys..

[63] Nawrocka W., Sztuba B., Kowalska M.W., Liszkiewicz H., Wietrzyk J., Nasulewicz A., Pełczyńska M., Opolski A. (2004). Synthesis and Antiproliferative Activity in Vitro of 2-Aminobenzimidazole Derivatives. Il Farmaco.

[64] Rene L., Poncet J., Auzou G. (1986). A One Pot Synthesis of β-Cyanoenamines. Synthesis.

[65] Dolomanov O.V., Bourhis L.J., Gildea R.J., Howard J.A.K., Puschmann H. (2009). OLEX2: A Complete Structure Solution, Refinement and Analysis Program. J. Appl. Crystallogr..

[66] Sheldrick G.M. (2015). SHELXT–Integrated Space-Group and Crystal-Structure Determination. Acta Crystallogr. A Found Adv..

[67] Marenich A.V., Cramer C.J., Truhlar D.G. (2009). Universal Solvation Model Based on Solute Electron Density and on a Continuum Model of the Solvent Defined by the Bulk Dielectric Constant and Atomic Surface Tensions. J. Phys. Chem. B.

