# Supplementary material for: 4-(Aryl)-Benzo[4,5]imidazo[1,2-a]pyrimidine-3-Carbonitrile-Based Fluorophores: Povarov Reaction-Based Synthesis, Photophysical Studies, and DFT Calculations
Source: Molecules. 2022 Nov 19;27(22):8029. doi: 10.3390/molecules27228029 (PMC9698514; doi:10.3390/molecules27228029)
Supplement: Supplementary file 1 [file molecules-27-08029-s001.zip › molecules-2033879-supplementary.pdf]

molecules

4-(Aryl)-Benzo[4,5]imidazo[1,2-*a*]pyrimidine-3-Carbonitrile-based Fluorophores: Povarov Reaction-based Synthesis, Photophysical Studies, and DFT Calculations

Victor V. Fedotov [1,*], Maria I. Valieva [1], Olga S. Taniya [1,*], Semen V. Aminov [1], Mikhail A. Kharitonov [1], Alexander S. Novikov [2], Dmitry S. Kopchuk [1], Pavel A. Slepukhin [1], Grigory V. Zyryanov [1], Evgeny N. Ulomsky [1], Vladimir L. Rusinov [1], Valery N. Charushin [1]

1 Chemical Engineering Institute, Ural Federal University, 19 Mira St., Yekaterinburg 620002, Russia
2 Institute of Chemistry, Saint Petersburg State University, 7/9 Universitetskaya Nab., Saint Petersburg 199034, Russia
* Correspondence: viktor.fedotov@urfu.ru

ELECTRONIC SUPPLEMENTARY INFORMATION

(ESI)

Table of contents

1. Fluorescence lifetime of probes **6a-f**......3
2. Absorption/fluorescence studies in solution and solvent effect 3
3. Preparation of PVA films......5
4. Aggregation study......5
5. Mechanochromic properties......6
6. Spectra of new compounds......7
7. Crystallography......33

1. Fluorescence lifetime of probes 6a-f

Table S1 Fluorescence lifetime of probes **6a-f** (C = 2×10^{-6} M) in THF

Entry	Compound	τ_1, ns[a]	α_1[b]	τ_2, ns[a]	α_2[b]	τ, ns[a]	χ^2[d]
**1**	**6a**	5.920708	10.29	2.024593	89.71	**2.43**	1.200343
**2**	**6b**	3.496655	10.04	5.310113	89.96	**5.12**	0.9998662
**3**	**6c**	9.415322	90.64	2.668804	9.36	**8.78**	1.140033
**4**	**6d**	1.233372	65.27	2.271551	34.73	**1.59**	1.227632
**5**	**6e**	7.656714	32.16	5.572208	67.84	**6.24**	1.102092
**6**	**6f**	2.025282	73.12	3.202319	26.88	**2.34**	1.130712

[a] Decay time, [b] Fractional contribution, [c] Weighted average decay time $\tau_{av} = \Sigma (\tau_i \times \alpha_i)$, [d] Quality of fitting

2. Absorption/fluorescence studies in solution and solvent effect

Table S2 Orientation polarizability for solvents (Δf), absorption and fluorescence emission maxima (λ_{abs}, λ_{em}, nm) and Stokes shift (nm, cm^{-1}) of **6a** in different solvents

Solvent	Δf	λ_{abs}, nm	λ_{em}, nm	Stokes shift, nm	Stokes shift, cm^{-1}
Cyclohexane	0.001	402	559	157	6986
Toluene	0.013	416	544	128	5656
THF	0.209	413	554	141	6162
DCM	0.218	424	547	123	5303
DMSO	0.276	416	-	-	-
MeCN	0.304	418	-	-	-

Table S3 Orientation polarizability for solvents (Δf), absorption and fluorescence emission maxima (λ_{abs}, λ_{em}, nm) and Stokes shift (nm, cm^{-1}) of **6b** in different solvents

Solvent	Δf	λ_{abs}, nm	λ_{em}, nm	Stokes shift, nm	Stokes shift, cm^{-1}
Cyclohexane	0.001	378	532	154	7658
Toluene	0.013	382	541	159	7693
THF	0.209	387	540	153	7321
DCM	0.218	392	538	148	6991
DMSO	0.276	375	537	162	8044
MeCN	0.304	376	534	158	7869
MeOH	0.308	373	536	163	8152

Table S4 Orientation polarizability for solvents (Δf), absorption and fluorescence emission maxima (λ_{abs}, λ_{em}, nm) and Stokes shift (nm, cm^{-1}) of **6c** in different solvents

Solvent	Δf	λ_{abs}, nm	λ_{em}, nm	Stokes shift, nm	Stokes shift, cm^{-1}
Cyclohexane	0.001	389	537	148	7085
Toluene	0.013	393	544	151	7062
THF	0.209	391	550	159	7393
DCM	0.218	392	546	154	7195
DMSO	0.276	394	550	156	7198
MeCN	0.304	390	550	160	7459
MeOH	0.308	389	557	168	7753

Table S5 Orientation polarizability for solvents (Δf), absorption and fluorescence emission maxima (λ_{abs}, λ_{em}, nm) and Stokes shift (nm, cm^{-1}) of **6d** in different solvents

Solvent	Δf	λ_{abs}, nm	λ_{em}, nm	Stokes shift, nm	Stokes shift, cm^{-1}
Cyclohexane	0.001	405	543	138	6275
Toluene	0.013	424	537	113	4963
THF	0.209	421	567	146	6116
DCM	0.218	432	556	124	5162
DMSO	0.276	427	-	-	-
MeCN	0.304	420	-	-	-

Table S6 Orientation polarizability for solvents (Δf), absorption and fluorescence emission maxima (λ_{abs}, λ_{em}, nm) and Stokes shift (nm, cm^{-1}) of **6e** in different solvents

Solvent	Δf	λ_{abs}, nm	λ_{em}, nm	Stokes shift, nm	Stokes shift, cm^{-1}
Cyclohexane	0.001	360	511	151	8208
Toluene	0.013	365	516	151	8017
THF	0.209	377	520	143	7294
DCM	0.218	367	517	150	7905
DMSO	0.276	377	466	89	5066
MeCN	0.304	375	517	142	7324
MeOH	0.308	370	518	148	7722

Table S7 Orientation polarizability for solvents (Δf), absorption and fluorescence emission maxima (λ_{abs}, λ_{em}, nm) and Stokes shift (nm, cm^{-1}) of **6f** in different solvents

Solvent	Δf	λ_{abs}, nm	λ_{em}, nm	Stokes shift, nm	Stokes shift, cm^{-1}
Cyclohexane	0.001	391	524	133	6491
Toluene	0.013	393	527	134	6469
THF	0.209	392	524	132	6426
DCM	0.218	392	526	134	6498
DMSO	0.276	394	548	154	7132
MeCN	0.304	391	534	143	6849
MeOH	0.308	389	570	181	8163

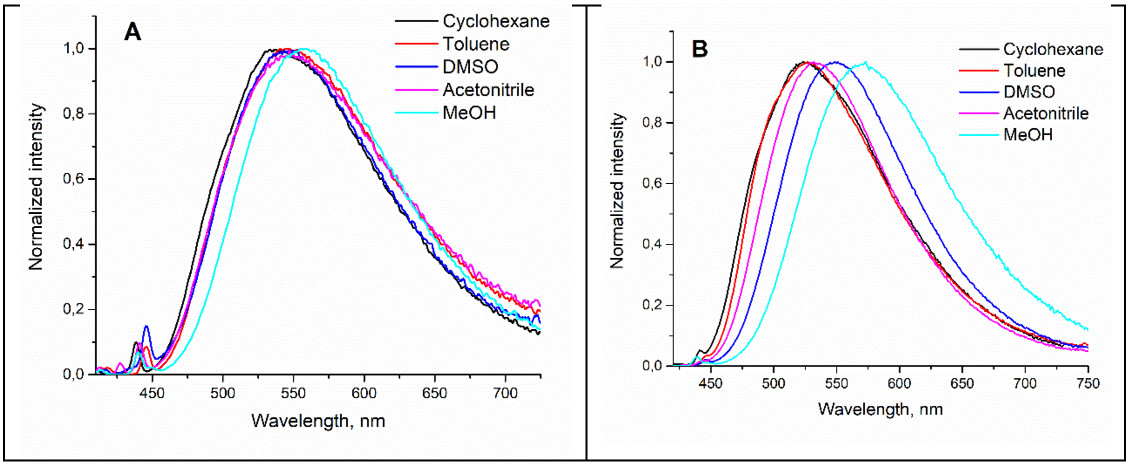

Figure S1. Solvent effect of **6c** and **6f**

3. Preparation of PVA films

Polyvinyl alcohol (PVA) (0.10 g) was dissolved in hot water (5000 μL). The solution of fluorophore (C = 10^{-3}M, 100 μL) in THF was added dropwise to the water mixture of oligomer under stirring, and then glycerol (30 μL) was added. The mixture was stirred for 5 more minutes, then the oligomer was spread on a substrate and left for a 24 h at room temperature. The finely concentration of chromophore was 10^{-5} M.

4. Aggregation study

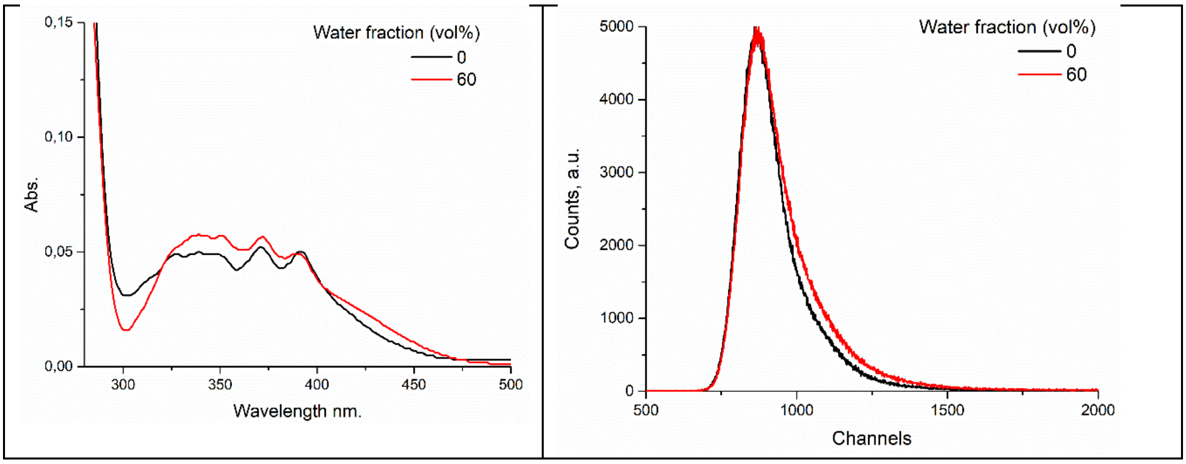

Figure S2. UV-*vis* absorption spectra of **6c** in THF/water mixtures with water fractions 0/60% (**A**). Time-resolved emission decay curves of **6c** in THF/water mixtures with water fractions 0/60% (**B**).

Table S8 Fluorescence lifetime of probe **6c** (C = 2×10^{-6} M) in THF/water mixtures with water fractions 0/60 (vol%)

Water fraction (vol%)	τ_1, ns[a]	α_1[b]	τ_2, ns[a]	α_2[b]	τ, ns[a]	χ^2[d]
**0**	9.415322	*90.64*	2.668804	*9.36*	8.78	1.140033
**60**	7.790384	81.20	3.116154	18.80	6.91	1.240673

[a] Decay time, [b] Fractional contribution, [c] Weighted average decay time $\tau_{av} = \Sigma\ (\tau_i \times \alpha_i)$, [d] Quality of fittin

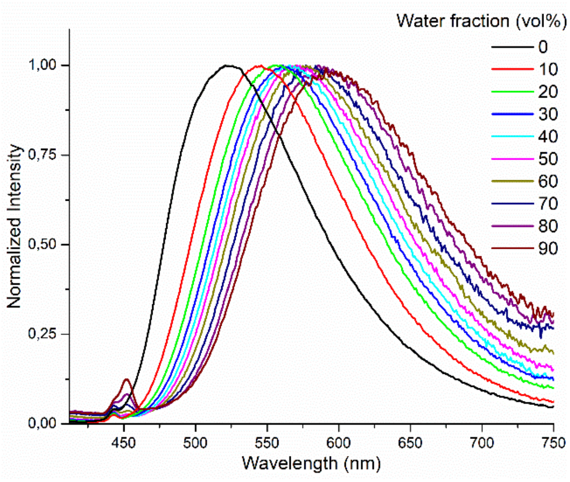

Figure S3. Solvent effect for **6f** in THF/water

5. Mechanochromic properties

Table S9. Mechanochromic properties of probes **6a-f**

#	Normal condition		After grinding		After fuming	
	λ_{em}^{max}, nm	Φ_f, (%)[a]	λ_{em}^{max}, nm	Φ_f, (%)[a]	λ_{em}^{max}, nm	Φ_f, (%)[a]
**6a**	572	20.5	567	23.2	562	20.8
**6b**	517	17.8	518	15.6	518	20.8
**6c**	511	3.9	542	4.9	522	5.8
**6d**	626	8.3	620	6.9	611	10.9
**6e**	509	19.3	512	10.5	510	19.4
**6f**	525	3.4	535	4.6	524	7.31

[a] Absolute quantum yields were measured using the Integrating Sphere of the Horiba -Fluoromax-4 at r.t. in powder form.

6. Spectra of new compounds

N-(4-Dimethylaminobenzylidene)-1H-benzo[d]imidazol-2-amine (3a)

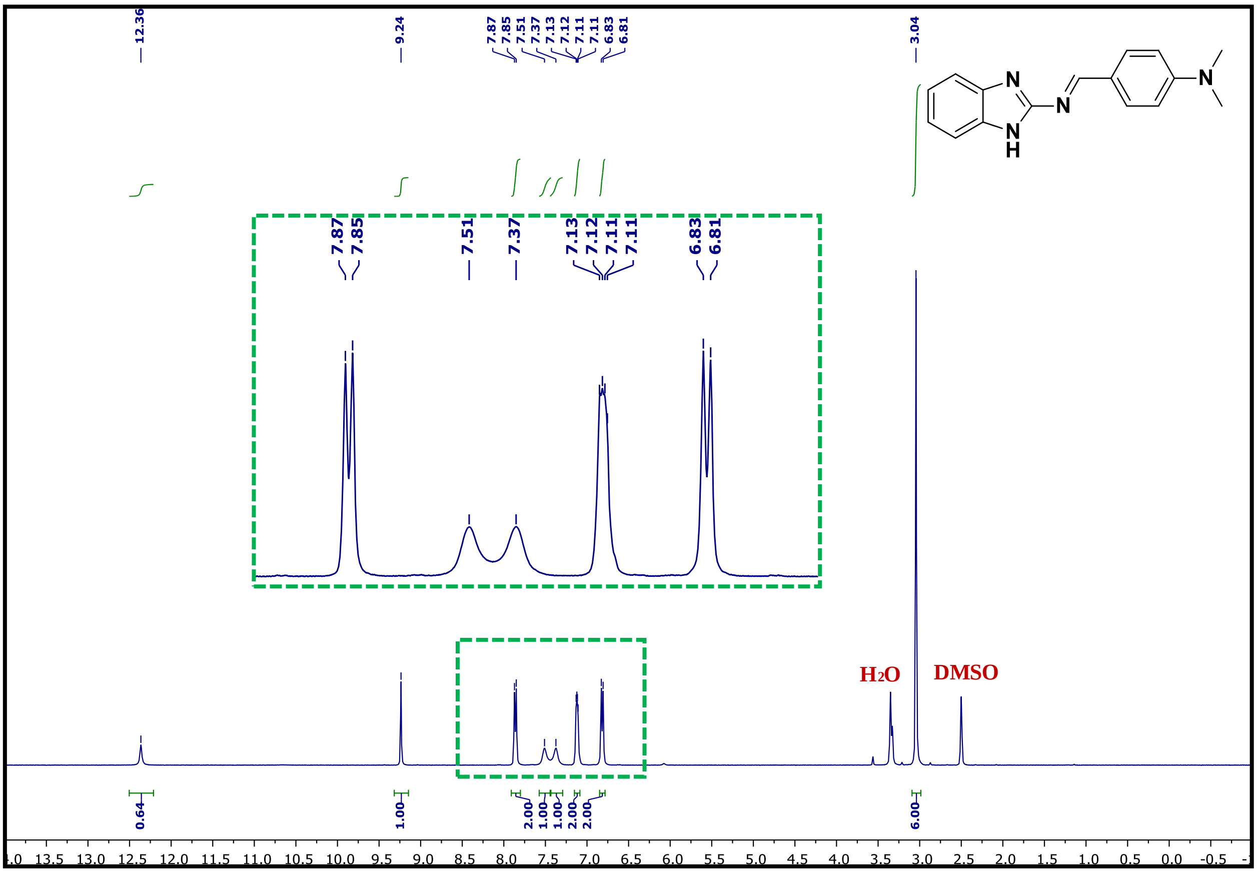

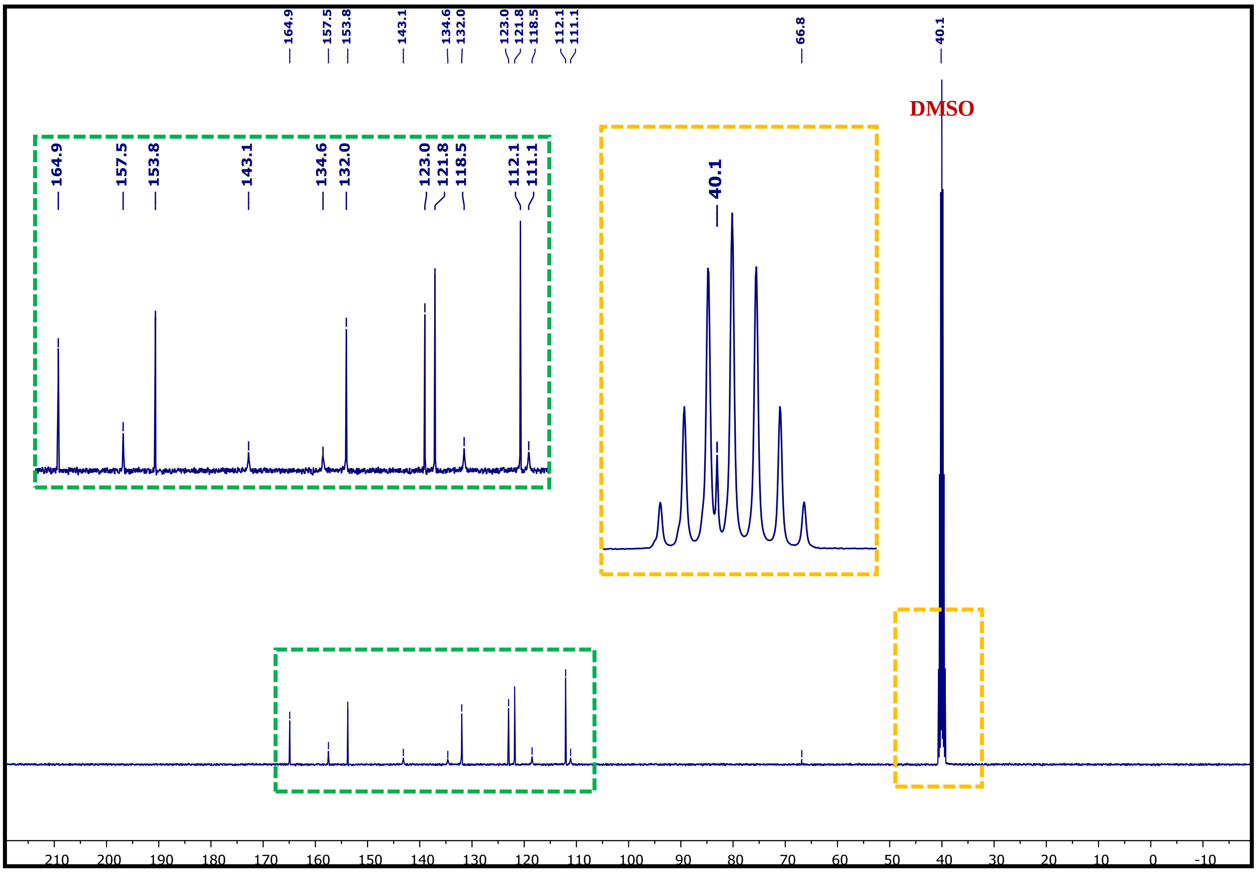

Figure S4. ^{1}H NMR (400 MHz, DMSO-d_6) and ^{13}C NMR (100 MHz, DMSO-d_6) spectra of **3a**

N-(anthracen-9-ylidene)-1H-benzo[d]imidazol-2-amine (3c)

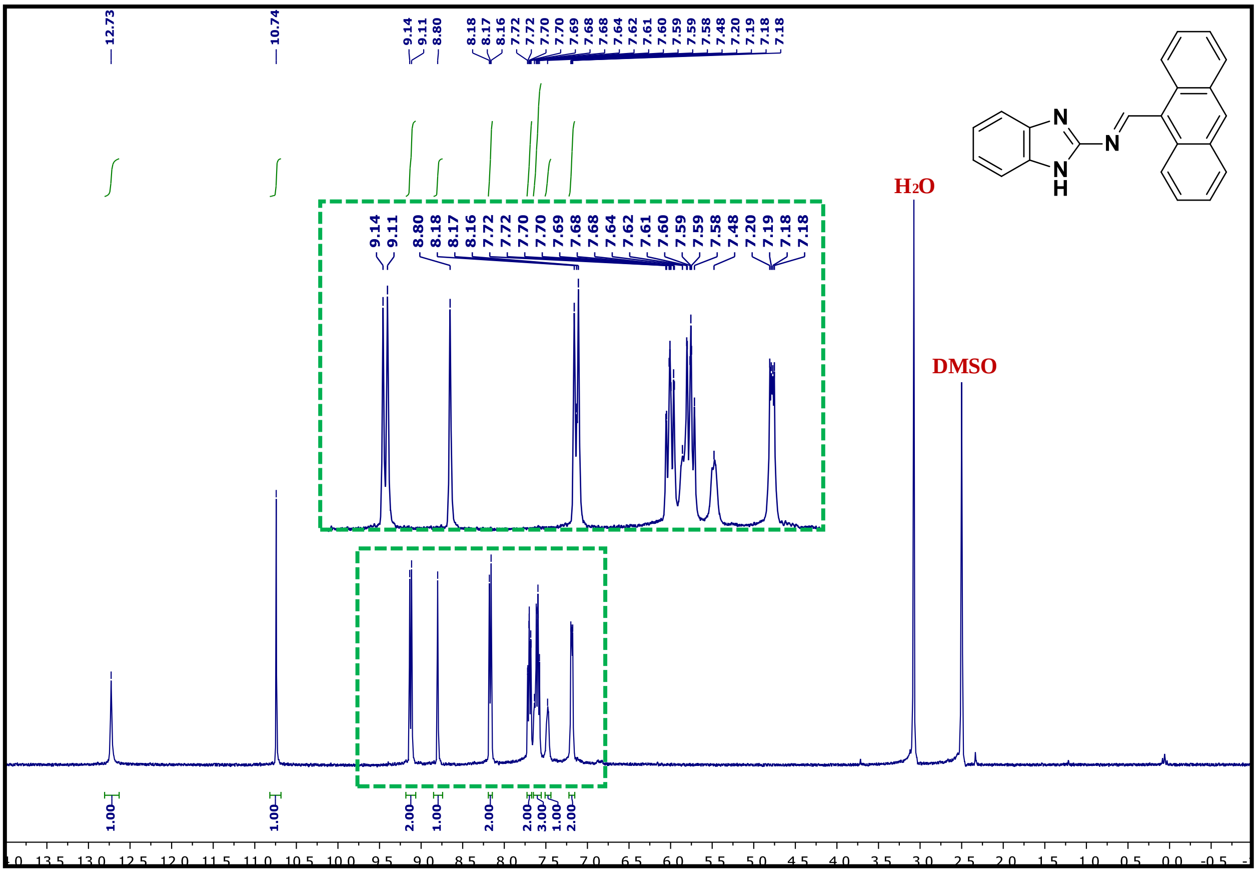

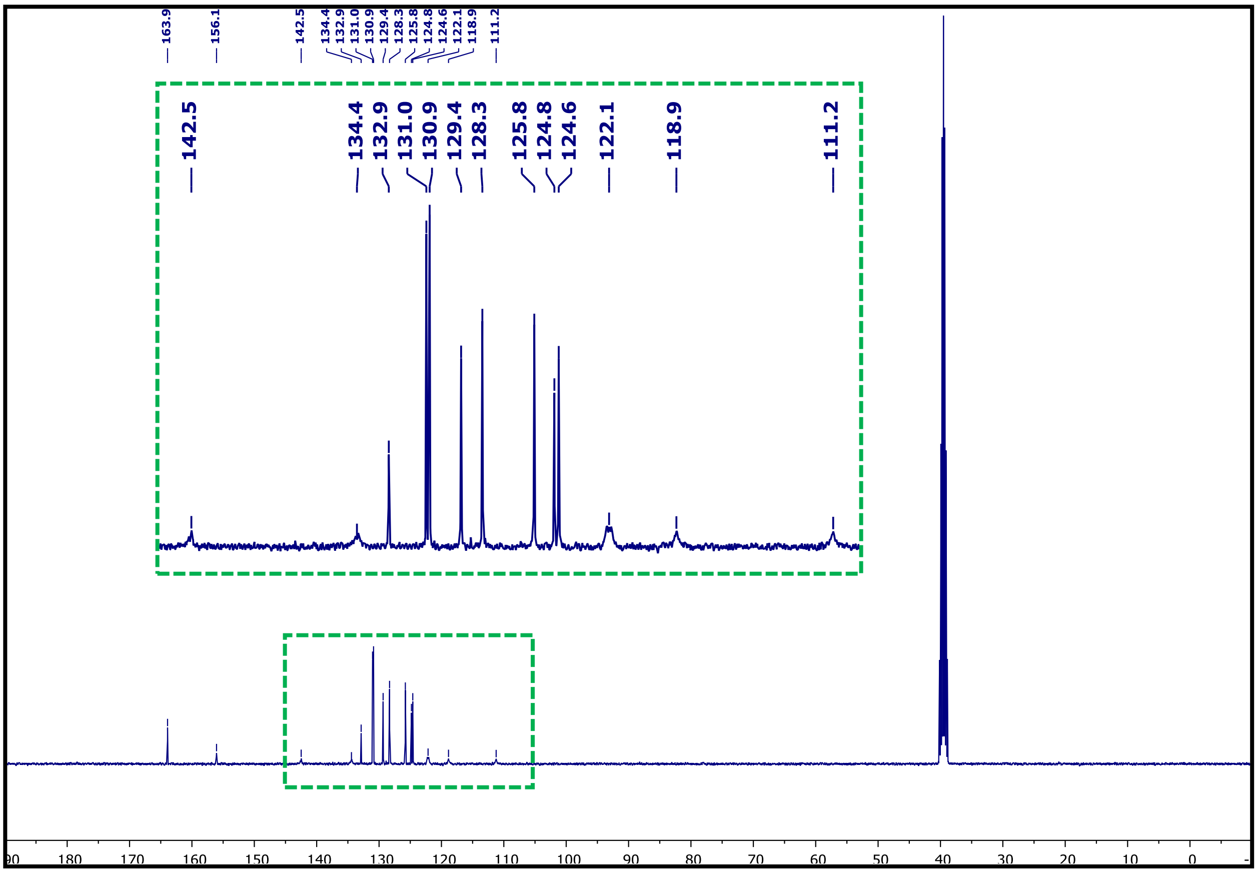

Figure S5. ^{1}H NMR (400 MHz, DMSO-d_6) and ^{13}C NMR (100 MHz, DMSO-d_6) spectra of **3c**

5,6-Difluoro-N-(4-dimethylaminobenzylidene)-1H-benzo[d]imidazol-2-amine (3d)

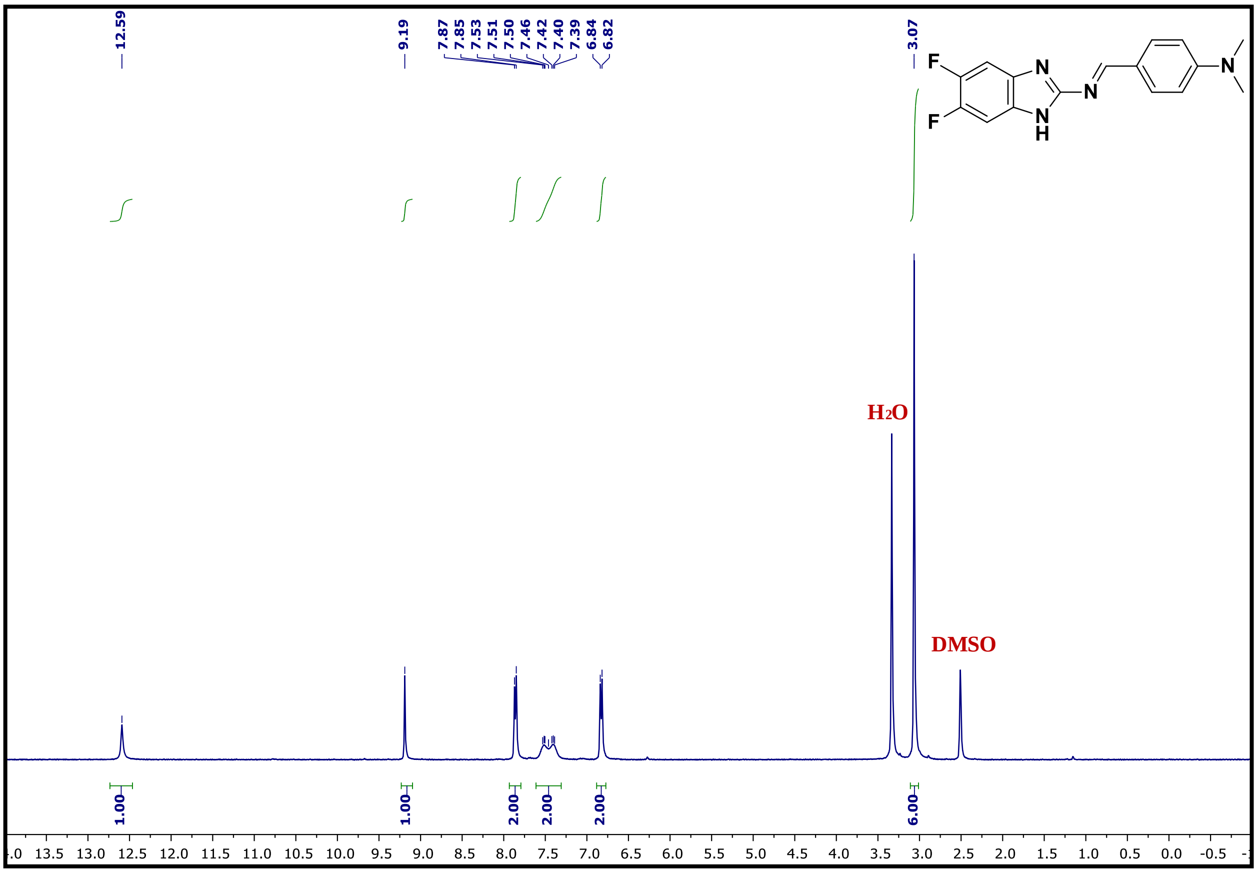

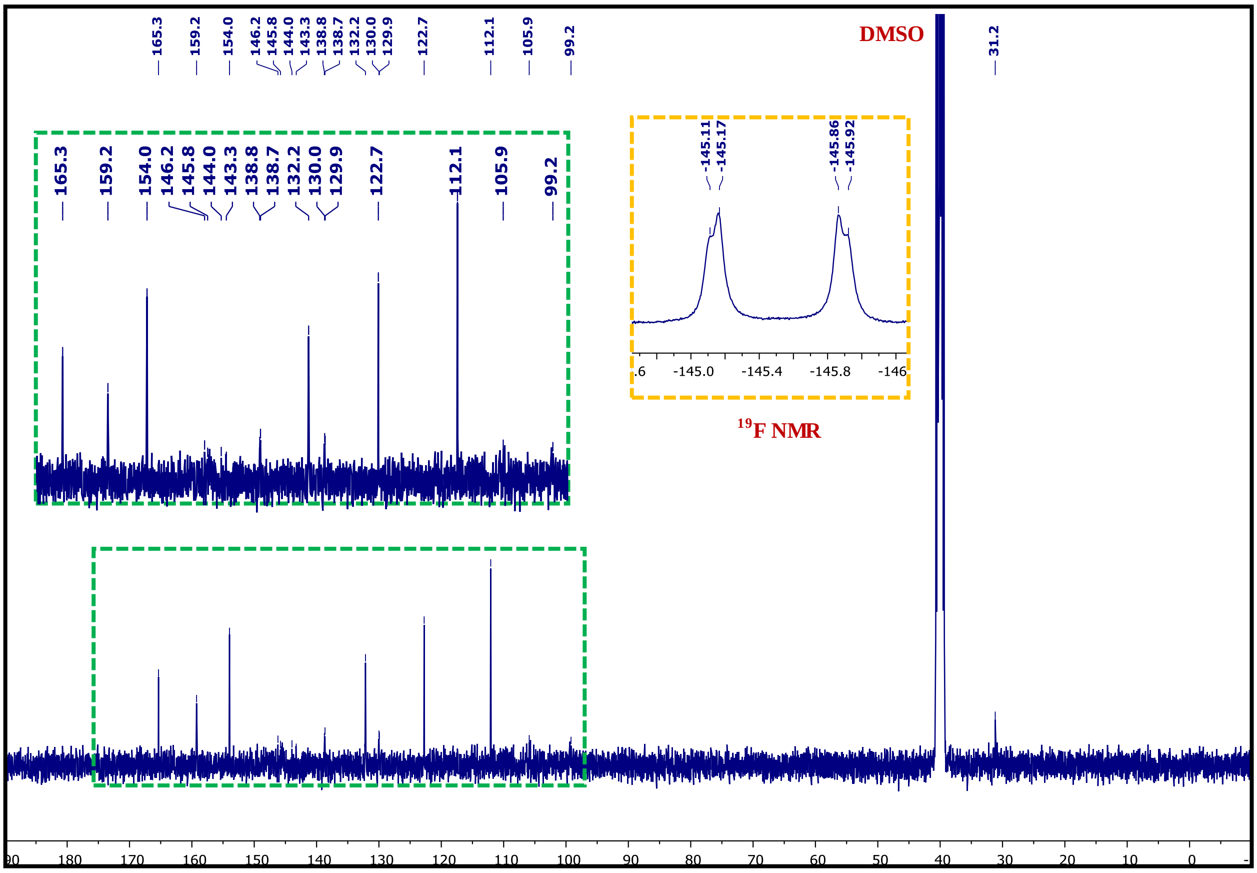

Figure S6. ^{1}H NMR (400 MHz, DMSO-d_6), ^{13}C NMR (100 MHz, DMSO-d_6), and ^{19}F NMR (376 MHz, DMSO-d_6) spectra of **3d**

5,6-Difluoro-N-(4-methoxybenzylidene)-1H-benzo[d]imidazol-2-amine (3e)

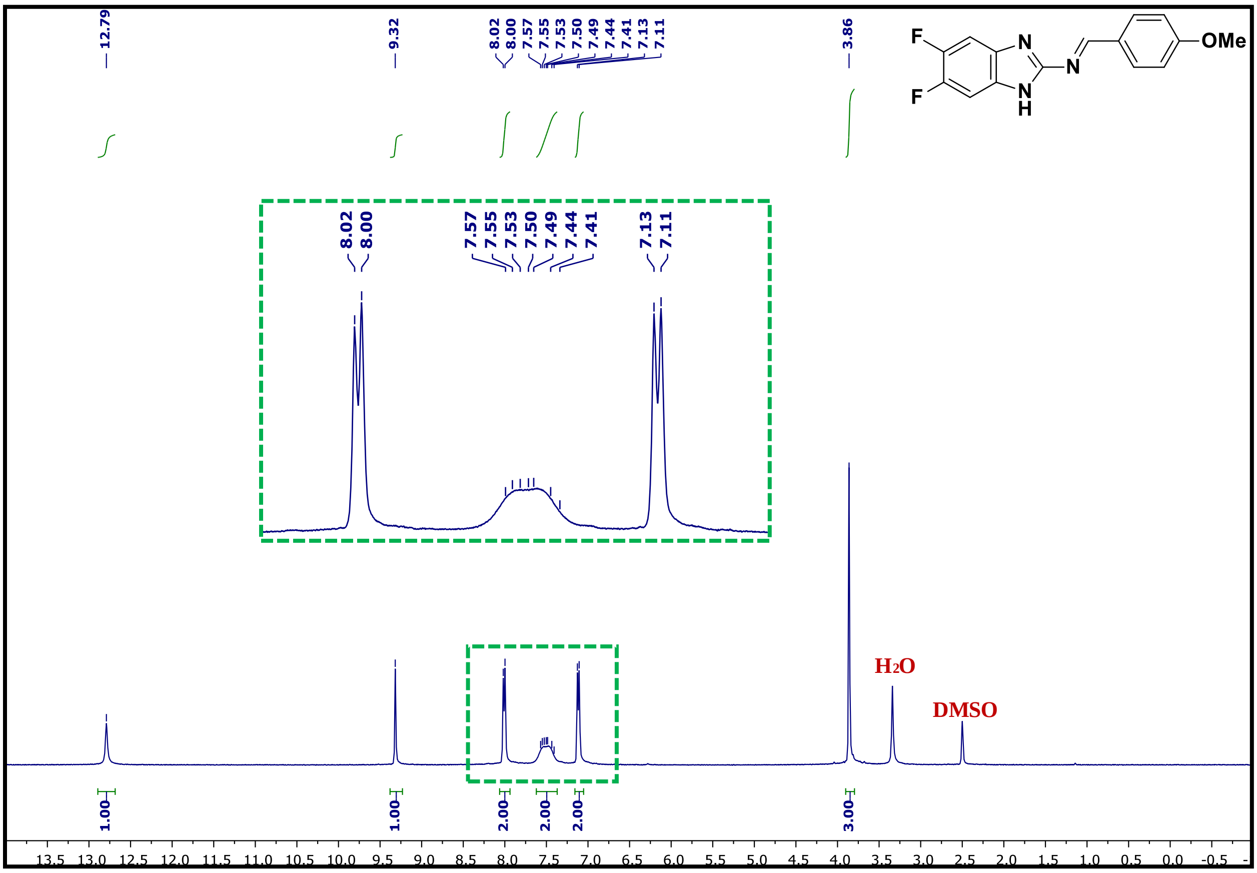

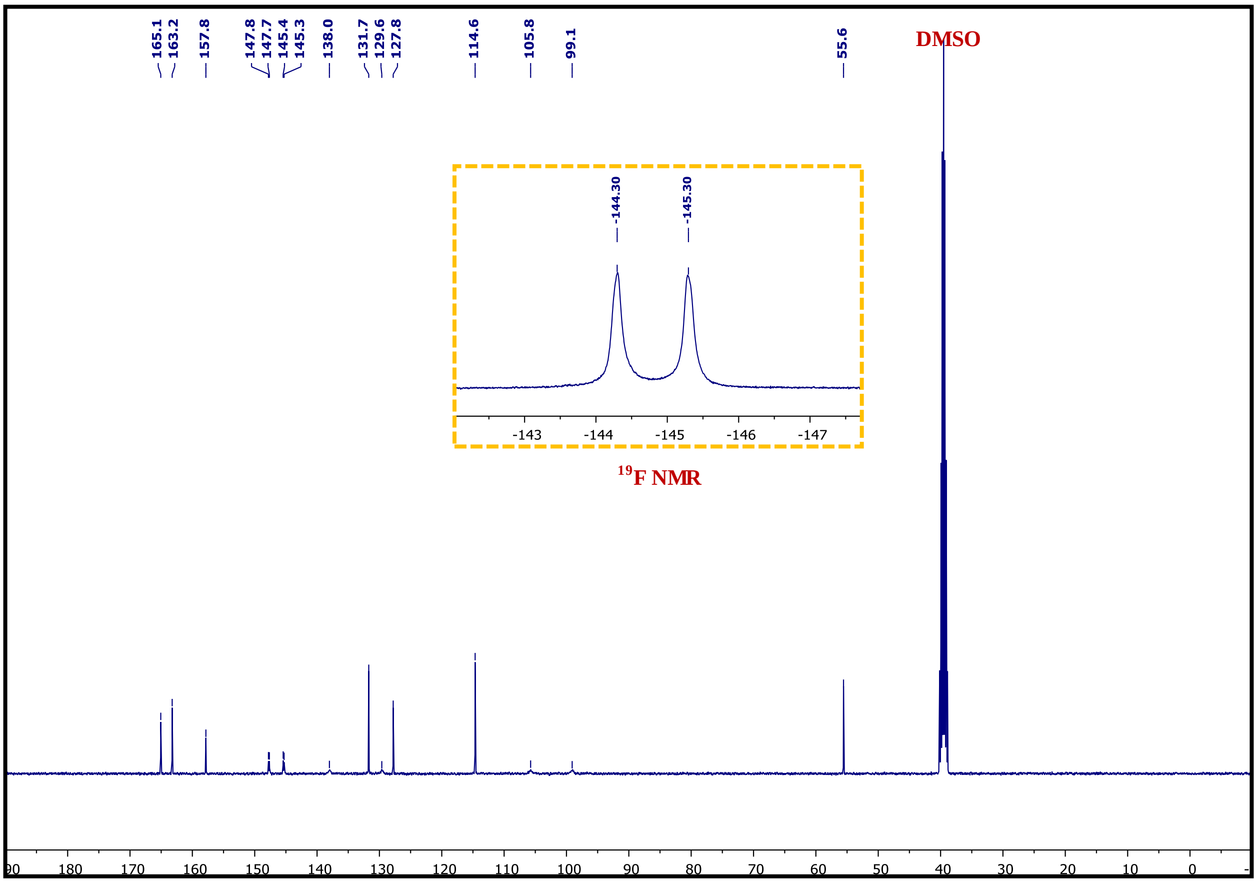

Figure S7. ^{1}H NMR (400 MHz, DMSO-*d*6), ^{13}C NMR (100 MHz, DMSO-*d*6), and ^{19}F NMR (376 MHz, DMSO-*d*6) spectra of **3e**

5,6-Difluoro-N-(anthracen-9-ylidene)-1H-benzo[d]imidazol-2-amine (3f)

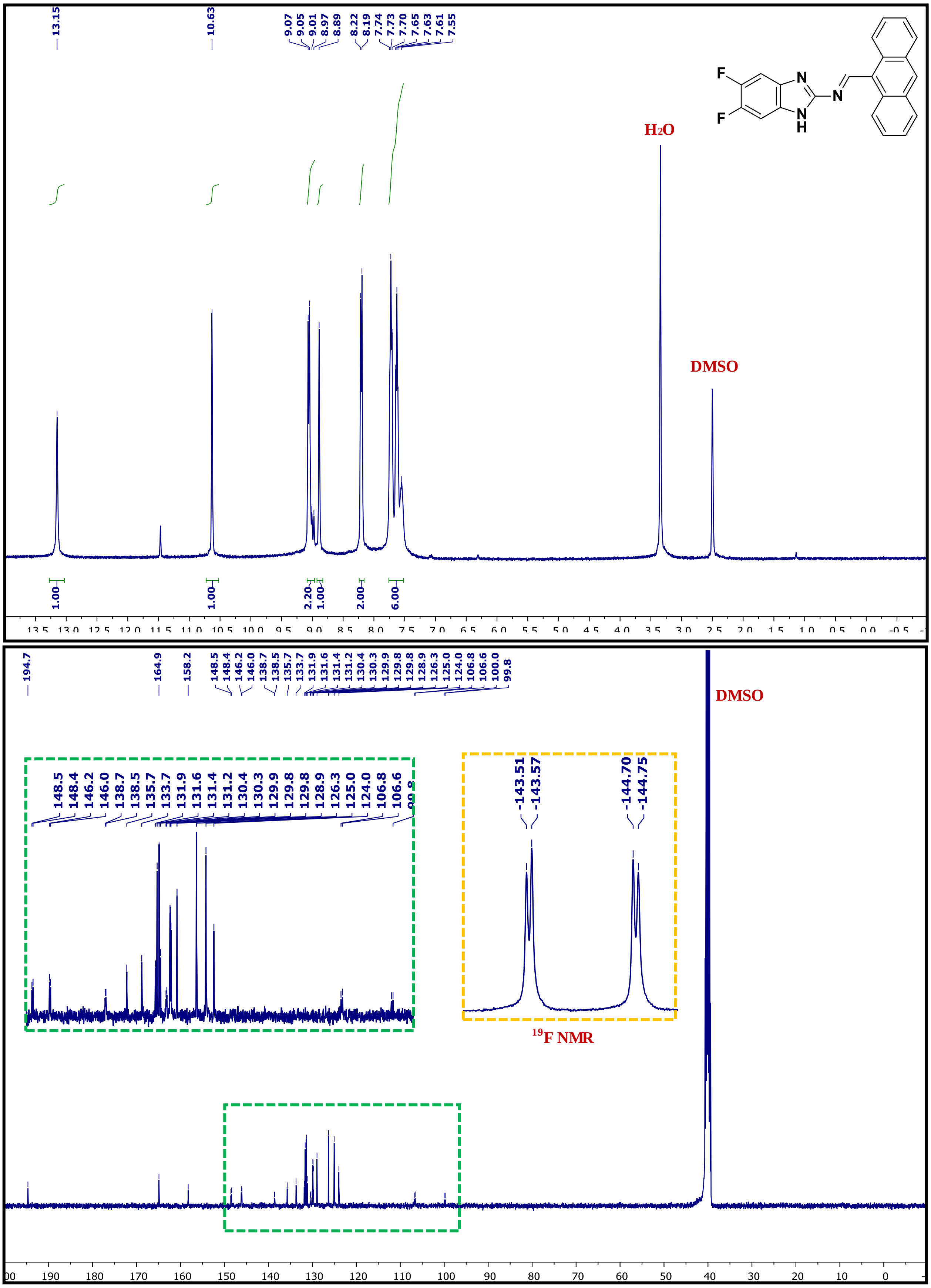

Figure S8. ^{1}H NMR (400 MHz, DMSO-d_6), ^{13}C NMR (100 MHz, DMSO-d_6), and ^{19}F NMR (376 MHz, DMSO-d_6) spectra of **3f**

4-(4-(Dimethylamino)phenyl)-1,2-dihydrobenzo[4,5]imidazo[1,2-a]pyrimidine-3-carbonitrile (5a)

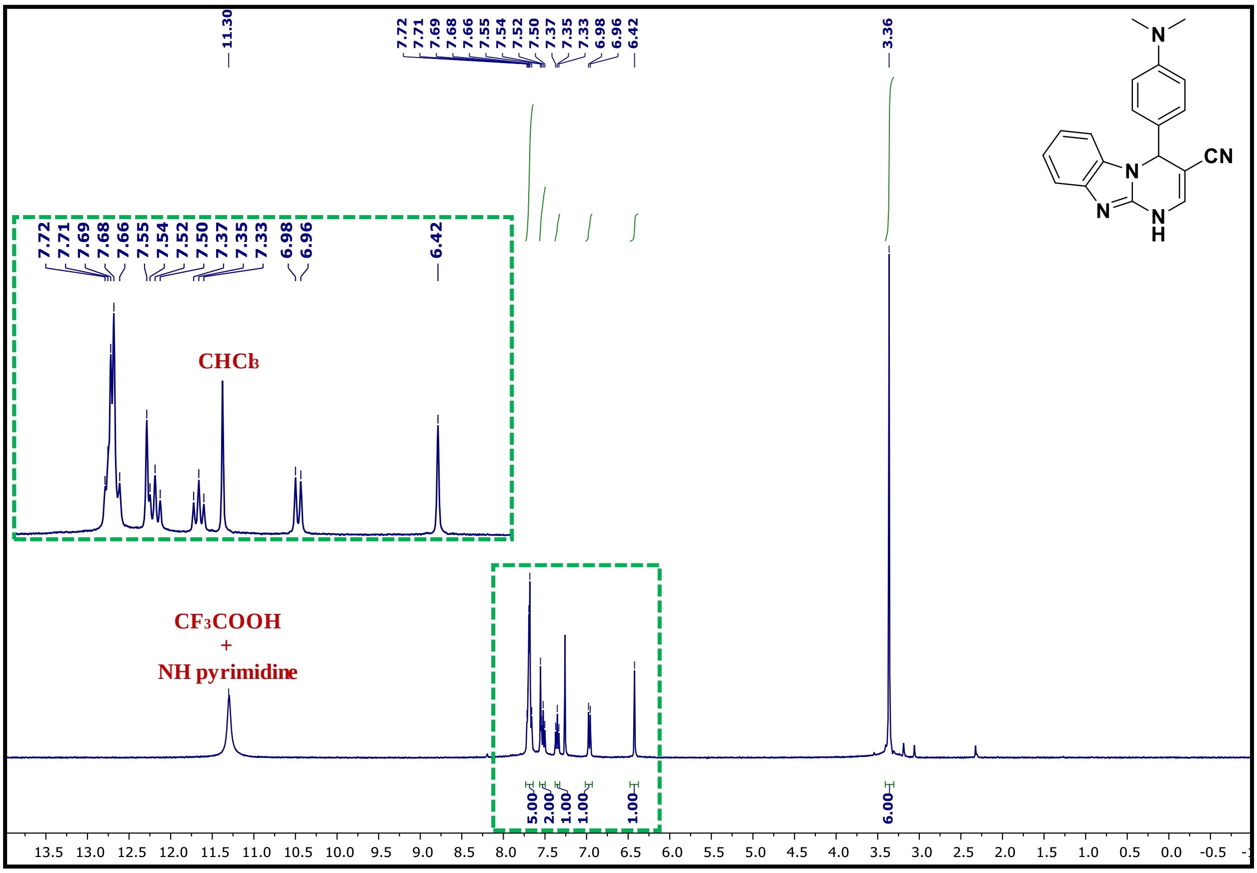

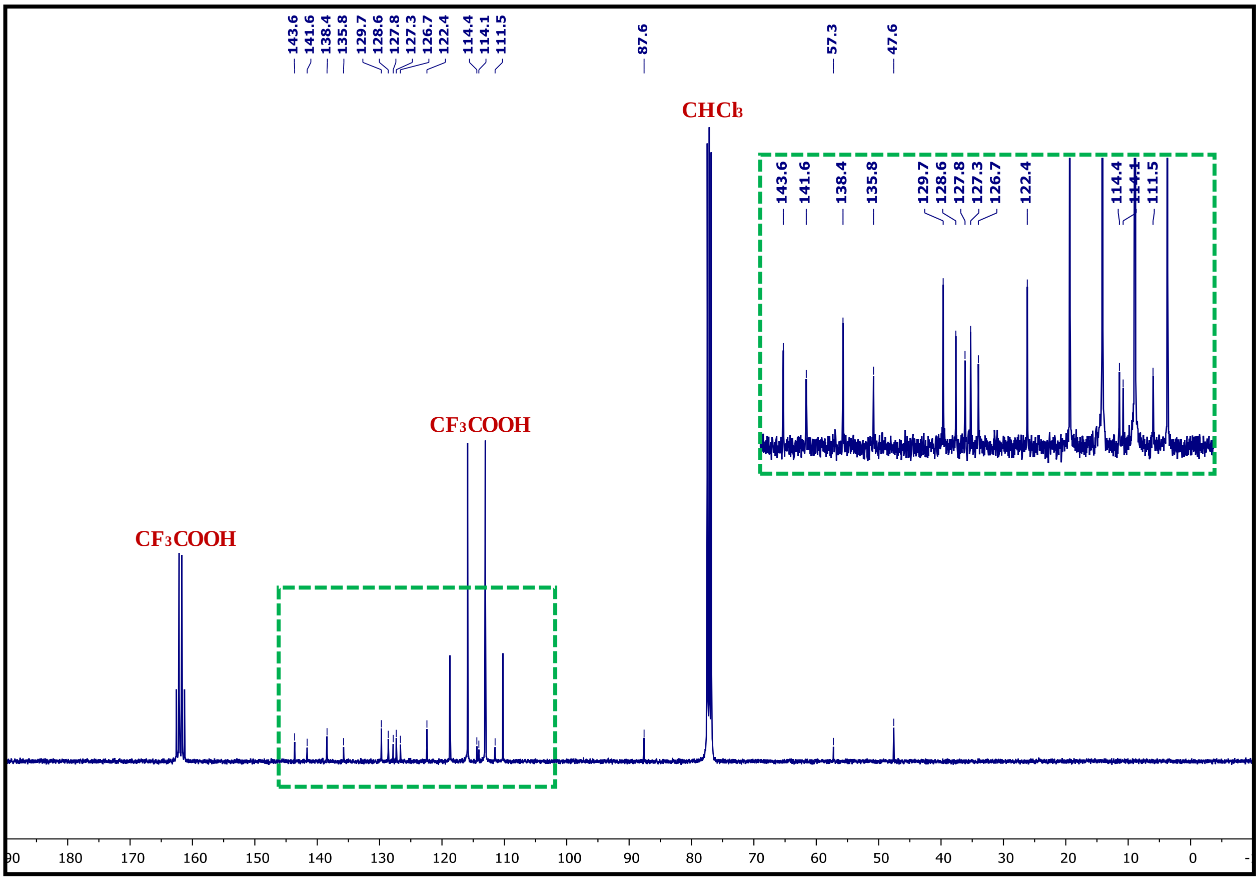

Figure S9. ^{1}H NMR ($CDCl_3$ + 0.1 ml CF_3COOD) and ^{13}C NMR (100 MHz, $CDCl_3$ + 0.1 ml CF_3COOD) spectra of **5a**

4-(4-Methoxyphenyl)-1,2-dihydrobenzo[4,5]imidazo[1,2-a]pyrimidine-3-carbonitrile (5b)

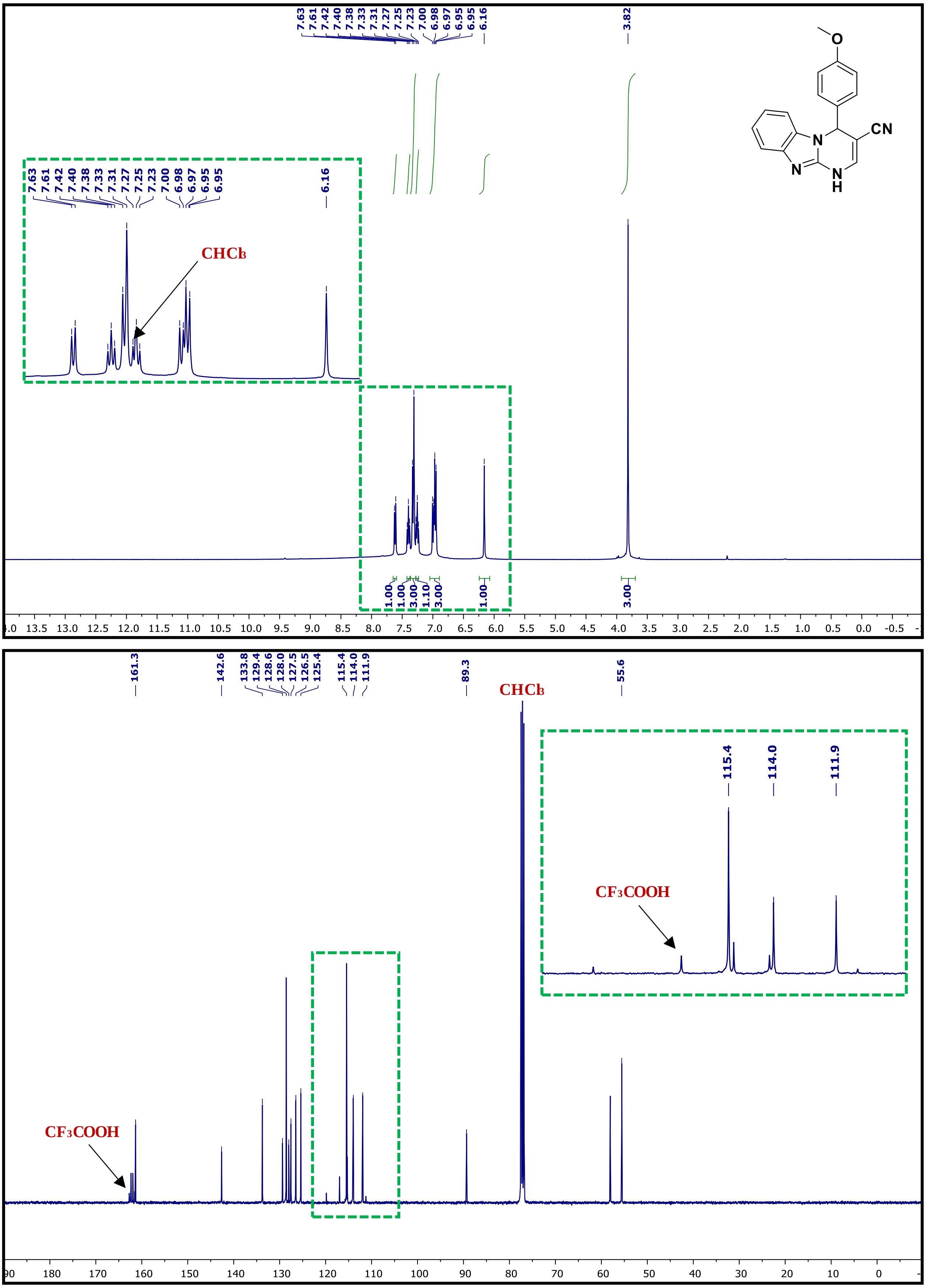

Figure S10. ^{1}H NMR ($CDCl_3$ + 0.1 ml CF_3COOD) and ^{13}C NMR (100 MHz, $CDCl_3$ + 0.1 ml CF_3COOD) spectra of **5b**

4-(Anthracen-9-yl)-1,2-dihydrobenzo[4,5]imidazo[1,2-a]pyrimidine-3-carbonitrile (5c)

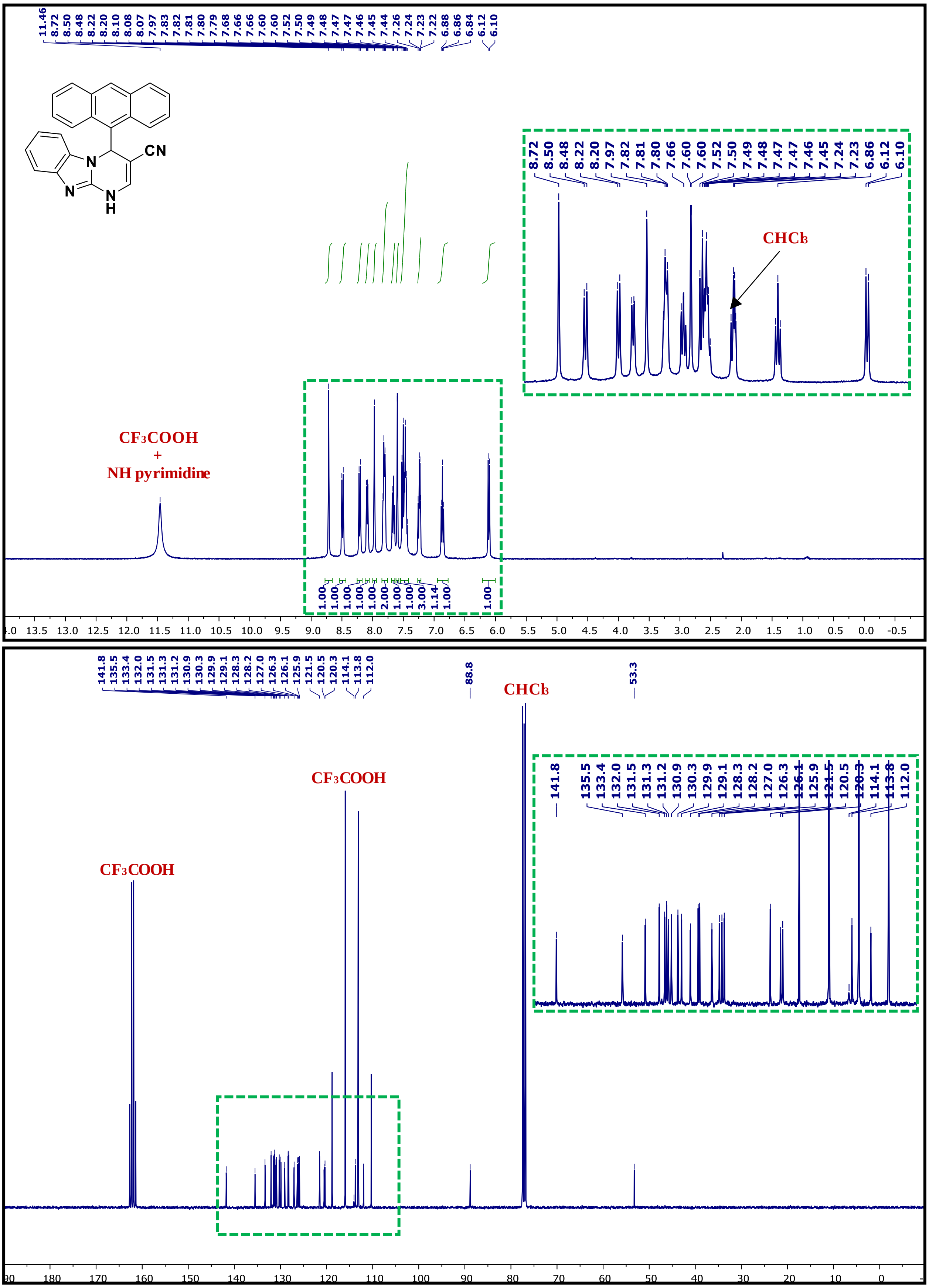

Figure S11. ^{1}H NMR ($CDCl_3$ + 0.1 ml CF_3COOD) and ^{13}C NMR (100 MHz, $CDCl_3$ + 0.1 ml CF_3COOD) spectra of **5c**

4-(4-(Dimethylamino)phenyl)-7,8-difluoro-1,2-dihydrobenzo[4,5]imidazo[1,2-a]pyrimidine-3-carbonitrile (5d).

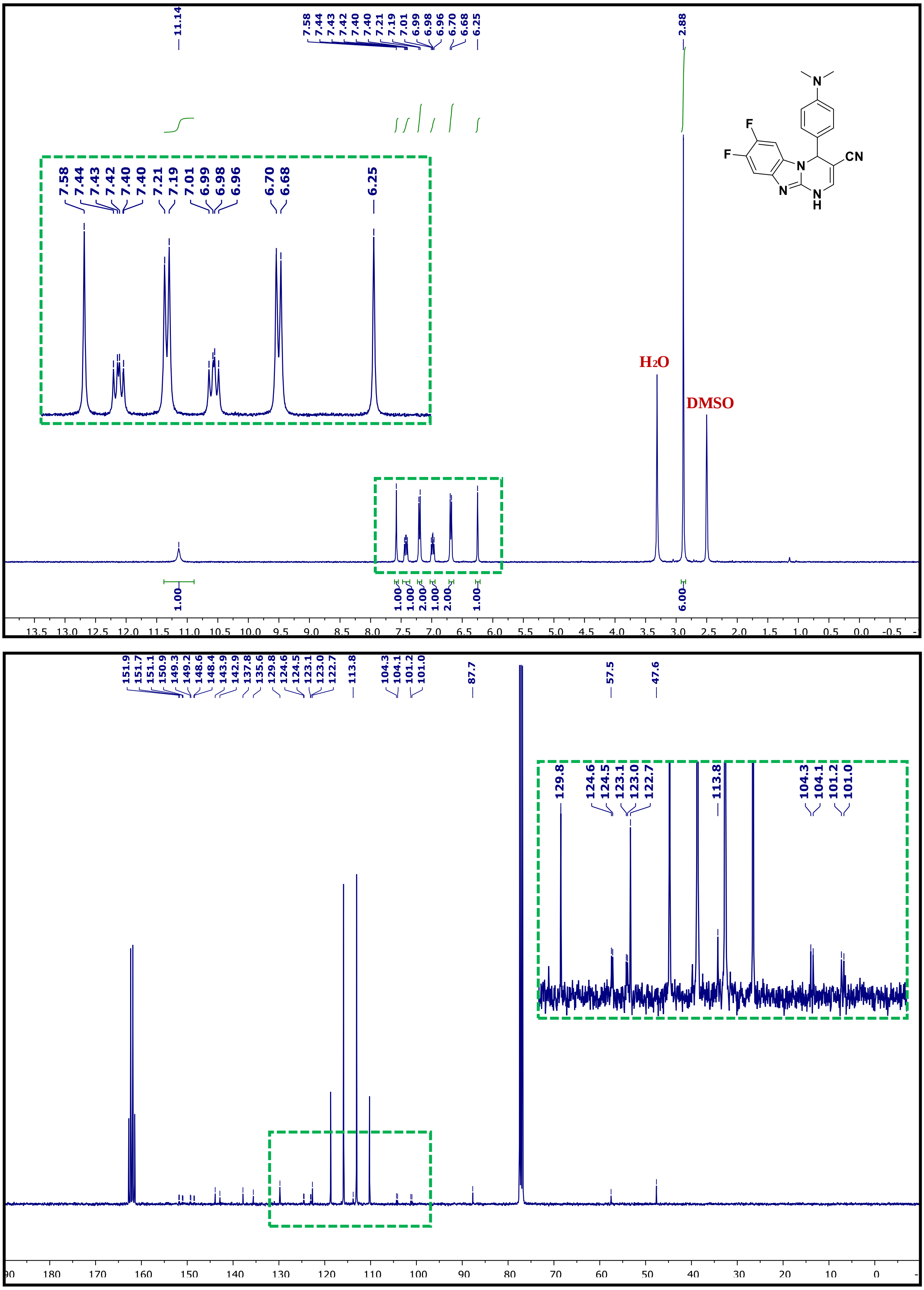

Figure S12. ^{1}H NMR (400 MHz, DMSO-d_6) and ^{13}C NMR (100 MHz, $CDCl_3$ + 0.1 ml CF_3COOD) spectra of **5d**

7,8-Difluoro-4-(4-methoxyphenyl)-1,2-dihydrobenzo[4,5]imidazo[1,2-a]pyrimidine-3-carbonitrile (5e)

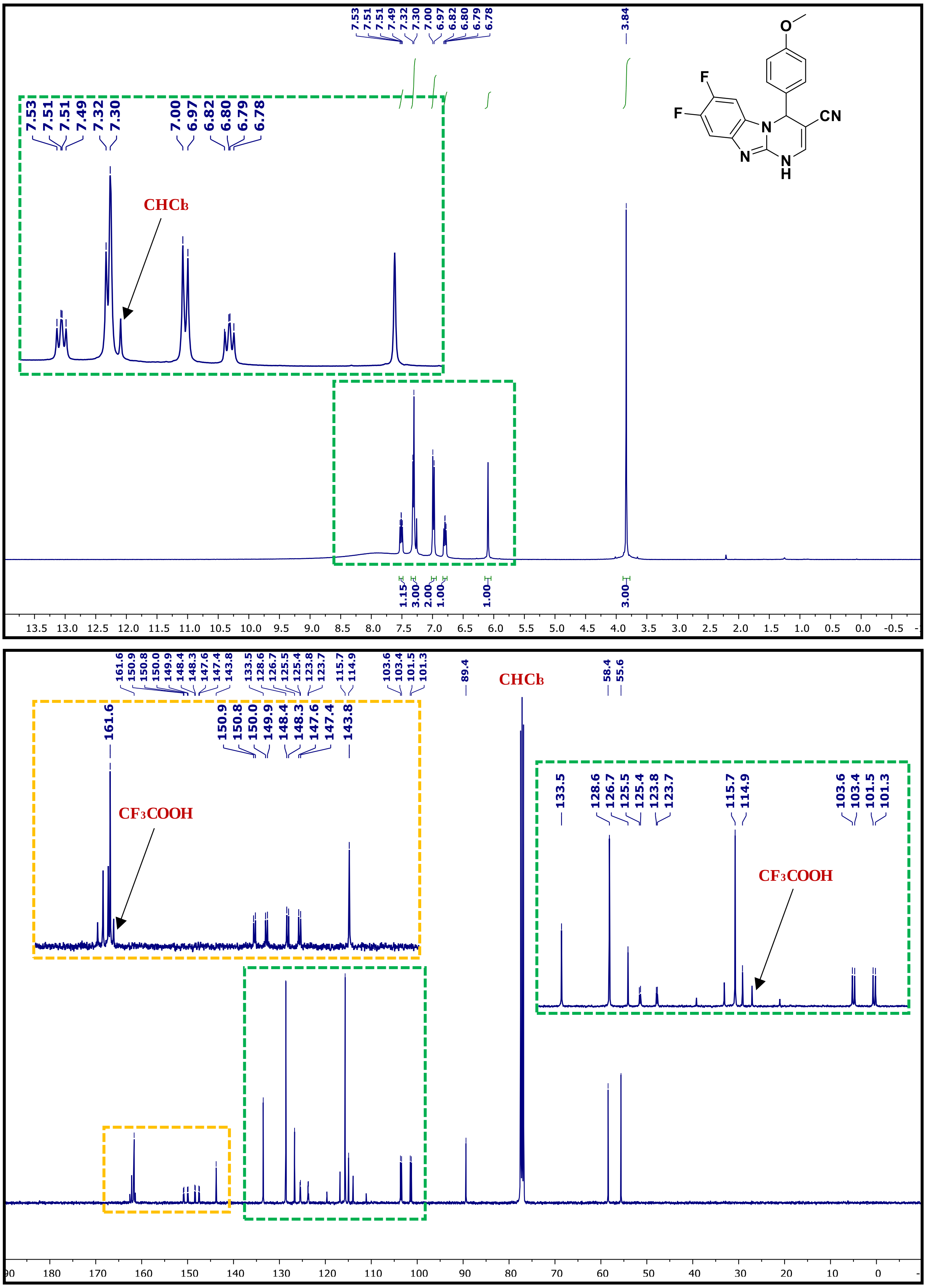

Figure S13. ^{1}H NMR ($CDCl_3$ + 0.1 ml CF_3COOD) and ^{13}C NMR (100 MHz, $CDCl_3$ + 0.1 ml CF_3COOD) spectra of **5e**

4-(Anthracen-9-yl)-7,8-difluoro-1,2-dihydrobenzo[4,5]imidazo[1,2-a]pyrimidine-3-carbonitrile (5f).

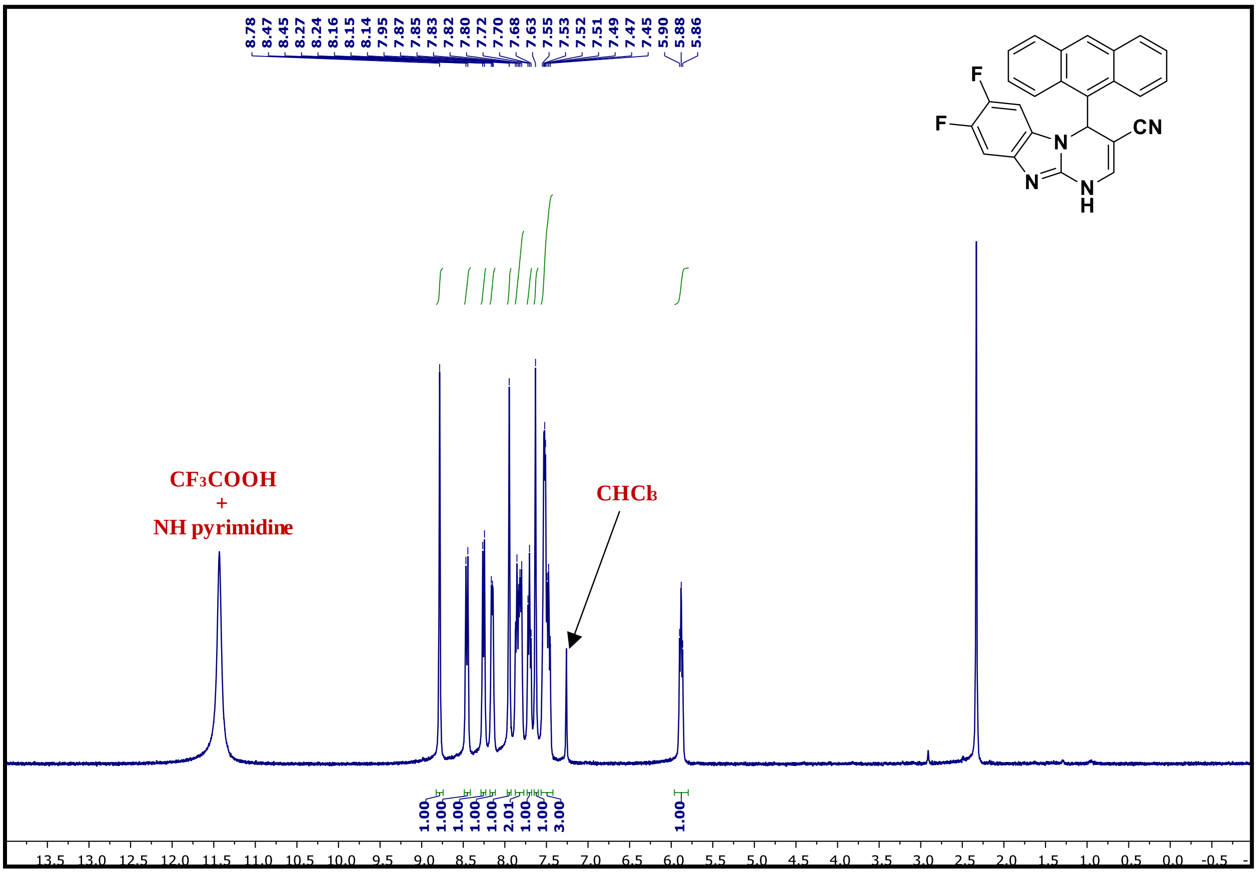

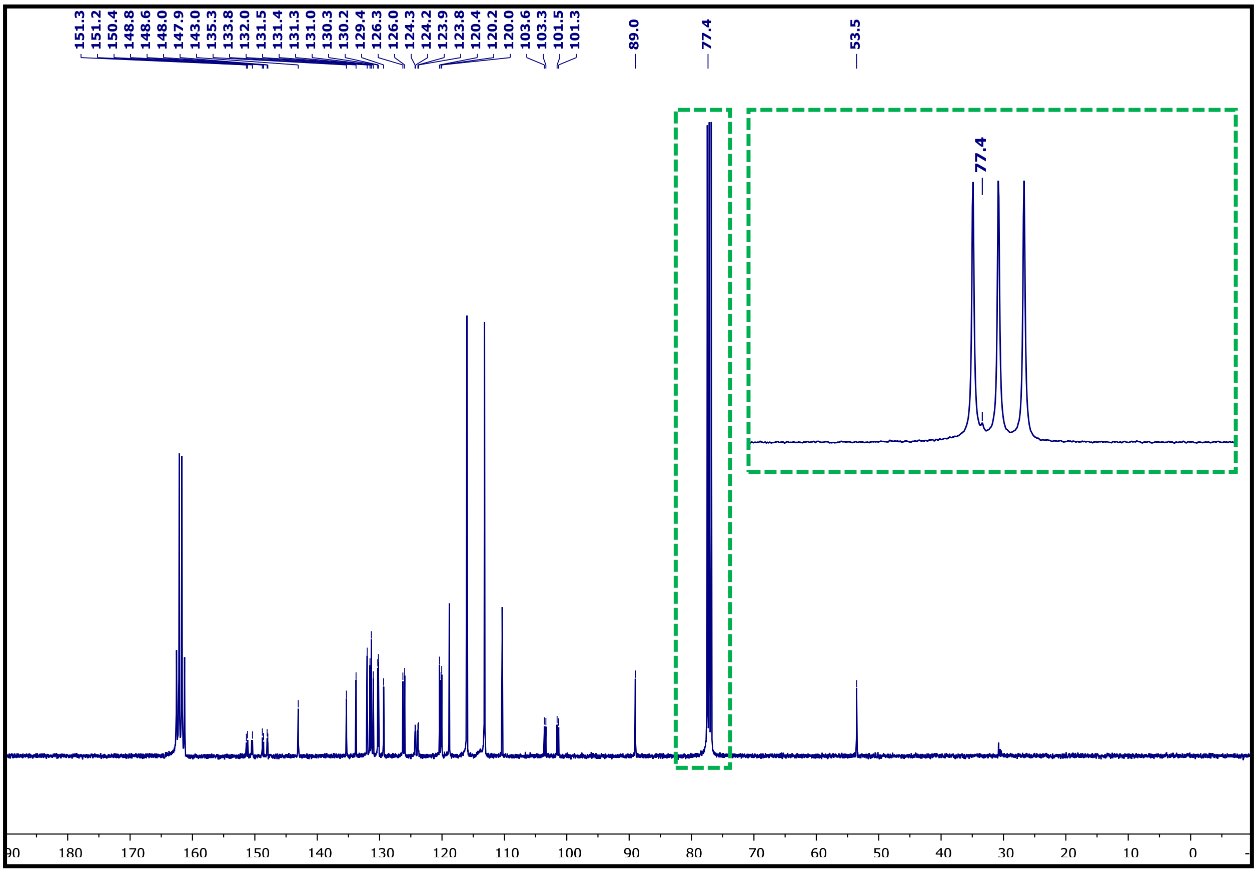

Figure S14. ^{1}H NMR ($CDCl_3$ + 0.1 ml CF_3COOD) and ^{13}C NMR (100 MHz, $CDCl_3$ + 0.1 ml CF_3COOD) spectra of **5f**

4-(4-(Dimethylamino)phenyl)benzo[4,5]imidazo[1,2-a]pyrimidine-3-carbonitrile (6a)

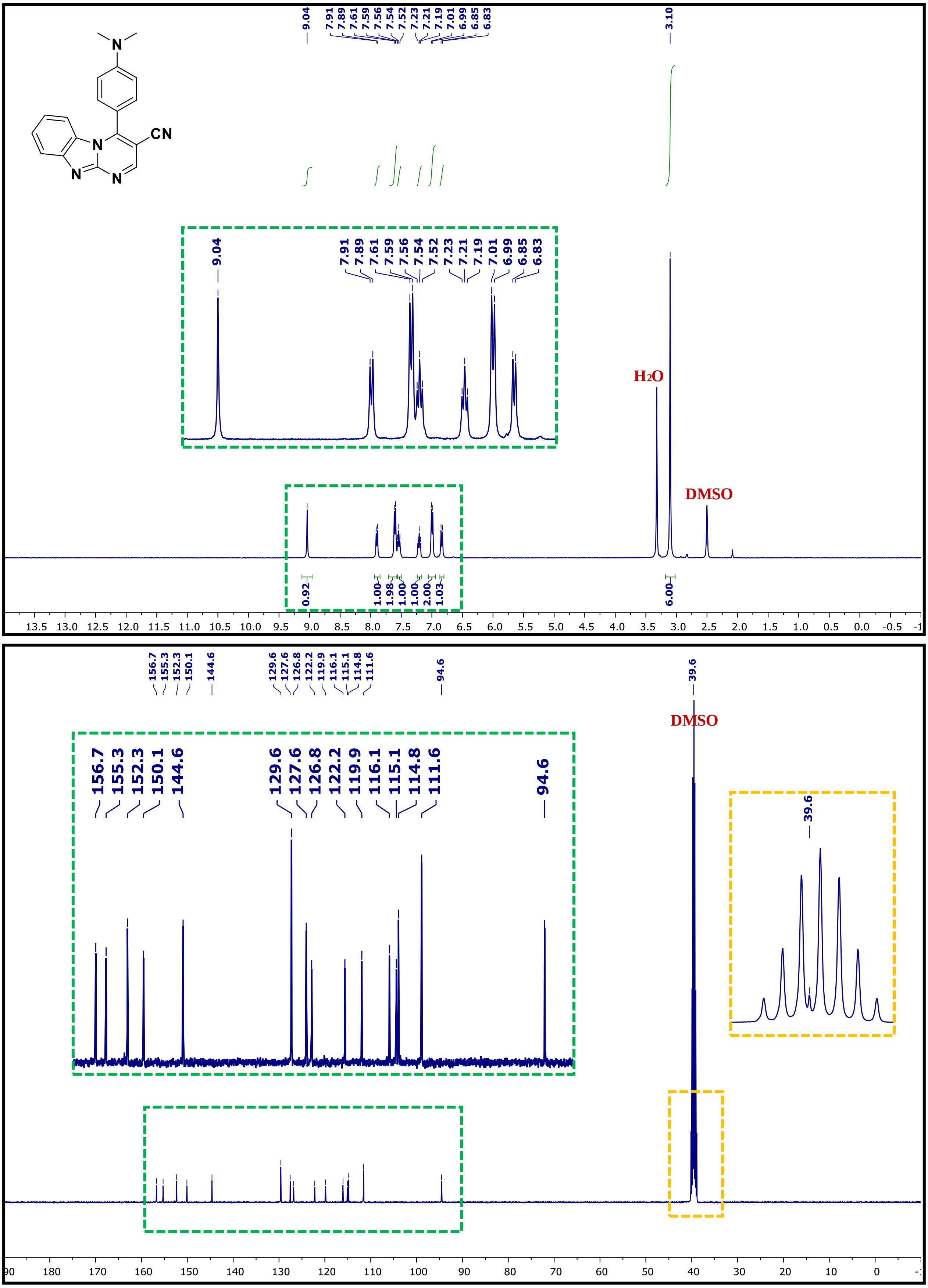

Figure S15. ^{1}H NMR (400 MHz, DMSO-d_6) and ^{13}C NMR (100 MHz, DMSO-d_6) spectra of **6a**

4-(4-Methoxyphenyl)benzo[4,5]imidazo[1,2-a]pyrimidine-3-carbonitrile (6b)

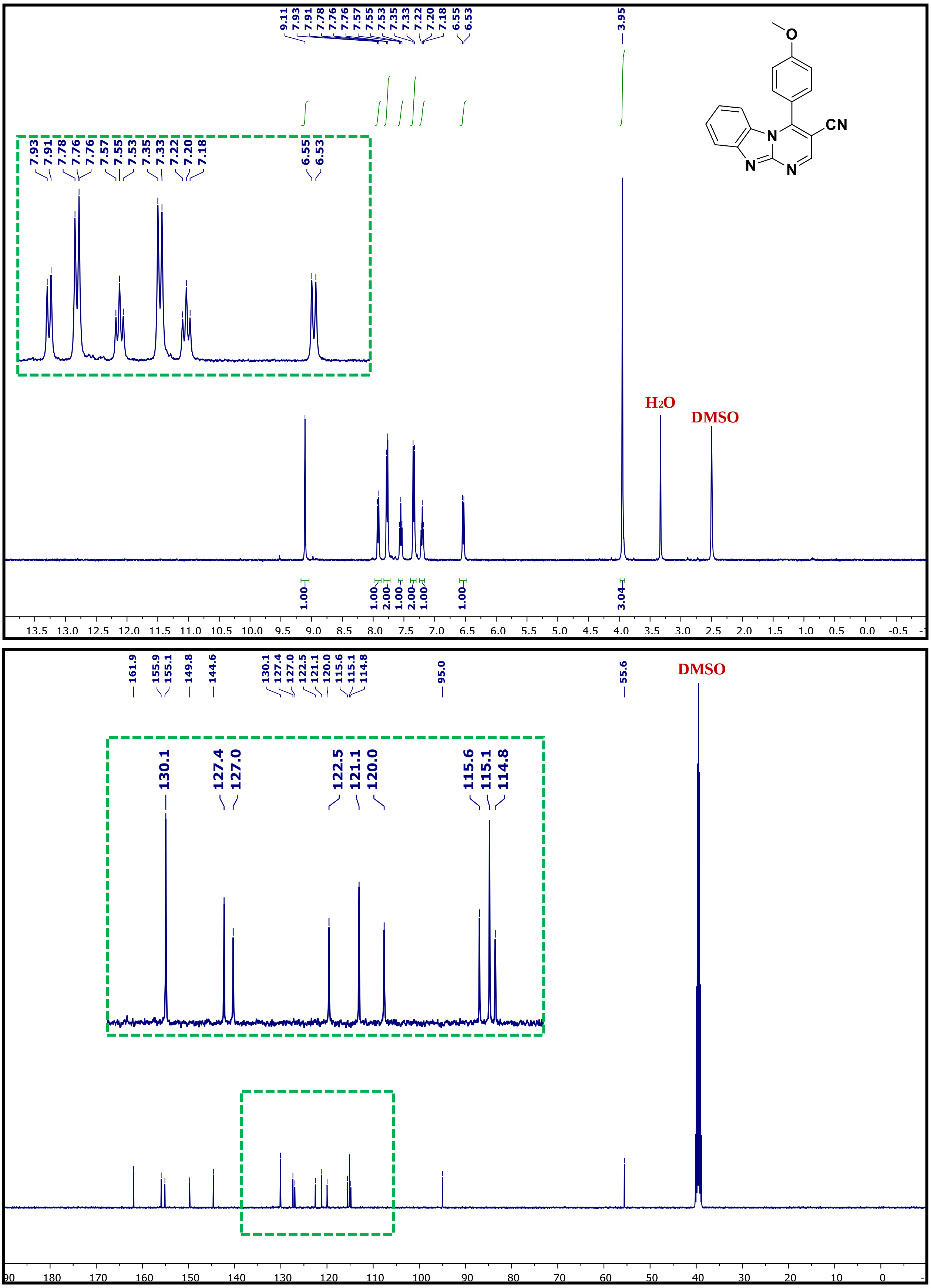

Figure S16. ^{1}H NMR (400 MHz, DMSO-*d*$_6$) and ^{13}C NMR (100 MHz, DMSO-*d*$_6$) spectra of **6b**

4-(Anthracen-9-yl)benzo[4,5]imidazo[1,2-a]pyrimidine-3-carbonitrile (6c)

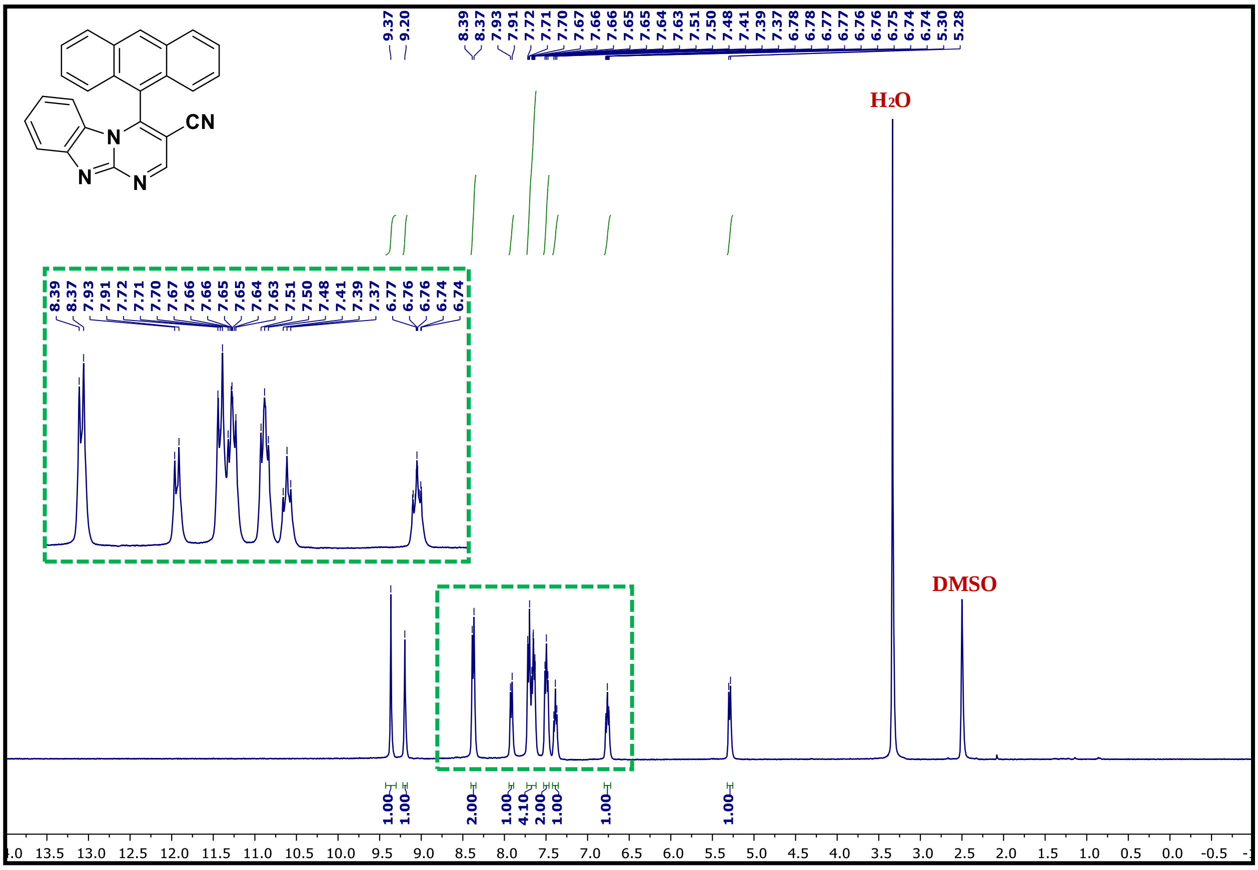

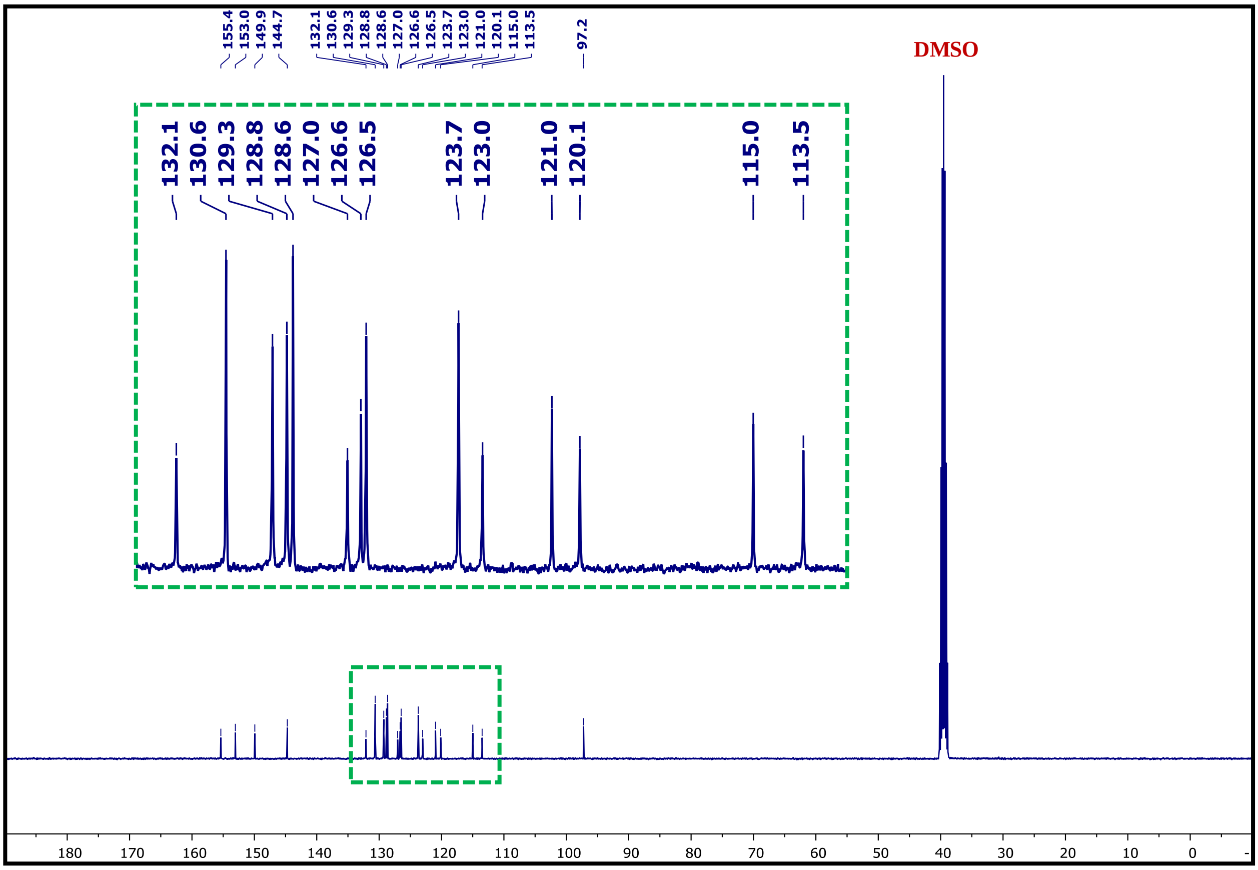

Figure S17. ^{1}H NMR (400 MHz, DMSO-d_6) and ^{13}C NMR (100 MHz, DMSO-d_6) spectra of **6c**

4-(4-(Dimethylamino)phenyl)-7,8-difluorobenzo[4,5]imidazo[1,2-a]pyrimidine-3-carbonitrile (6d)

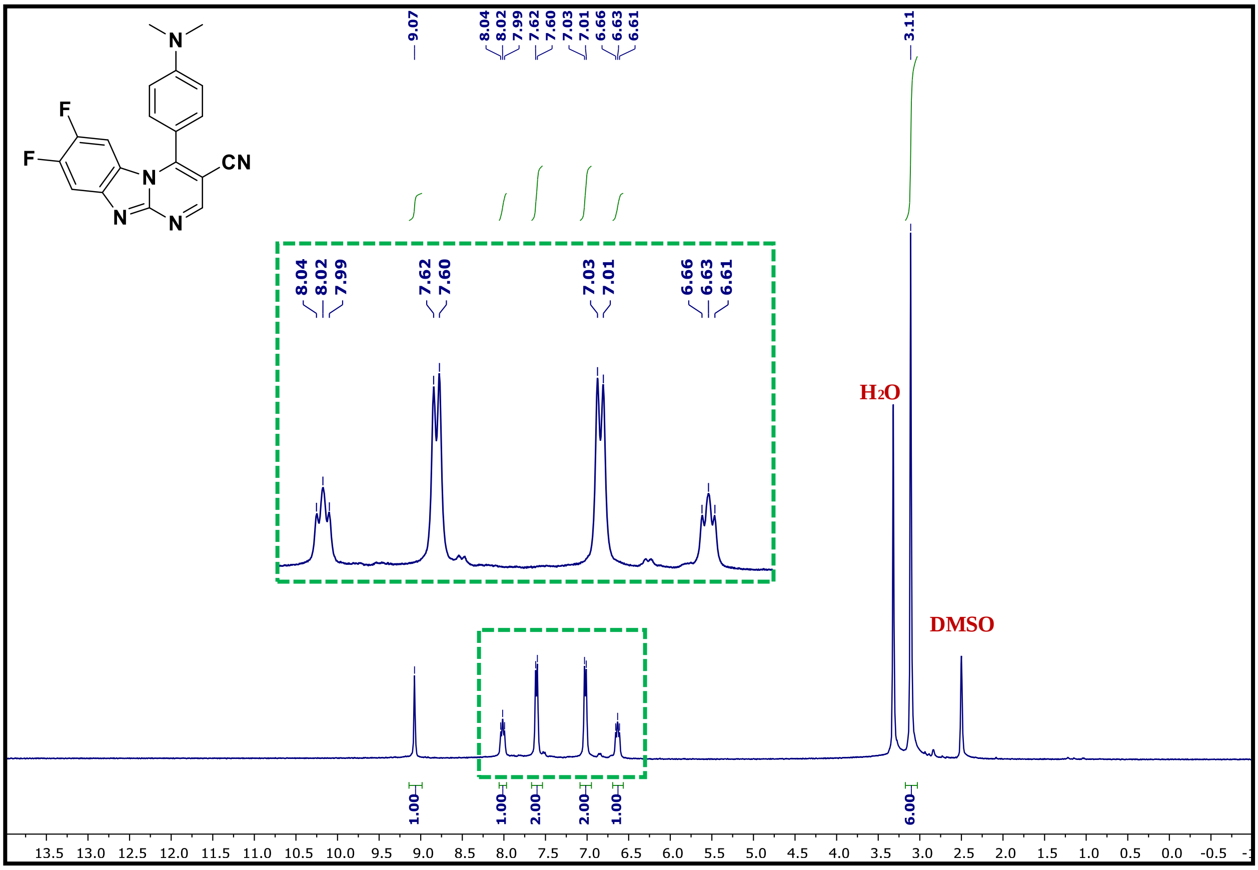

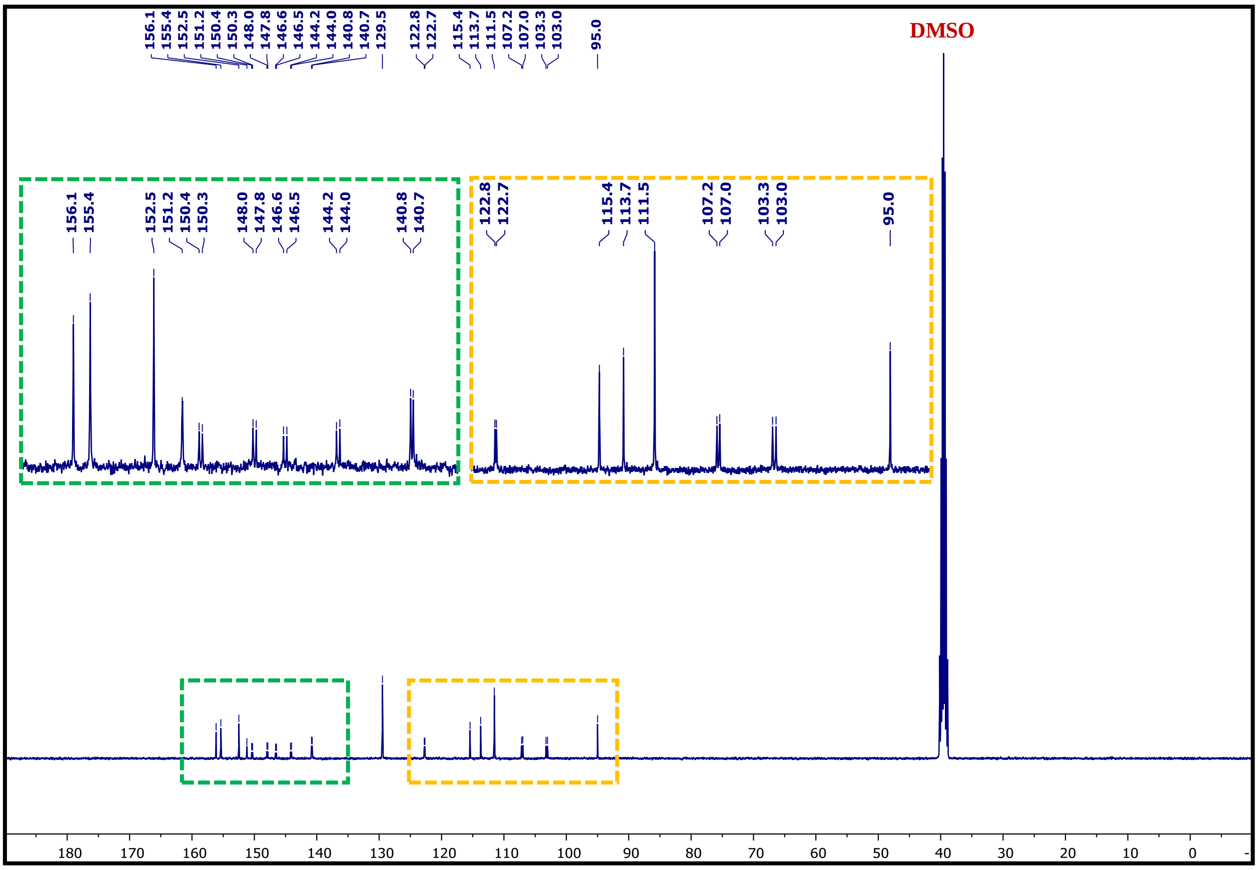

Figure S18. ^{1}H NMR (400 MHz, DMSO-d_6) and ^{13}C NMR (100 MHz, DMSO-d_6) spectra of **6d**

7,8-Difluoro-4-(4-methoxyphenyl)benzo[4,5]imidazo[1,2-a]pyrimidine-3-carbonitrile (6e)

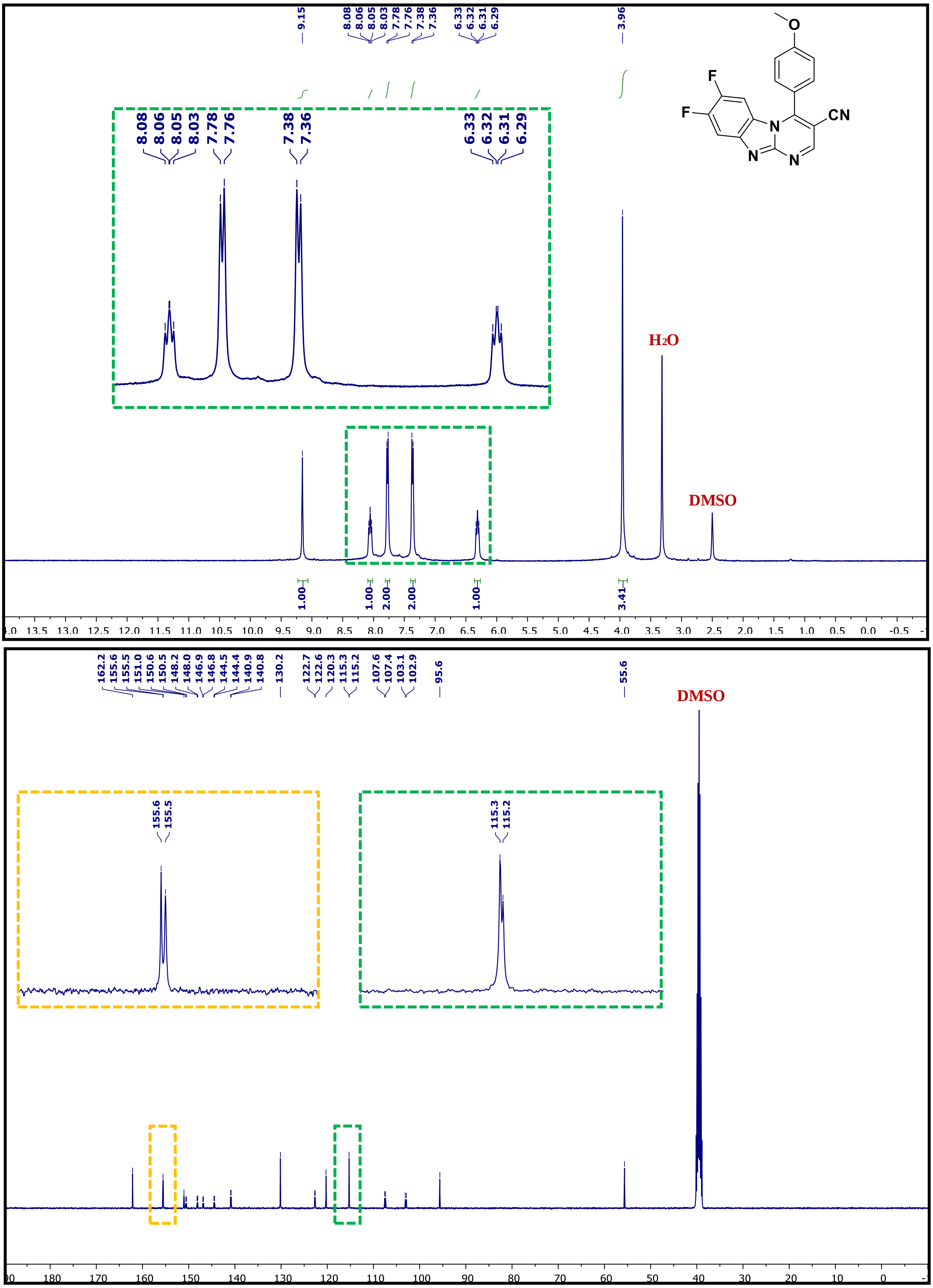

Figure S19. ^{1}H NMR (400 MHz, DMSO-d_6) and ^{13}C NMR (100 MHz, DMSO-d_6) spectra of **6e**

4-(Anthracen-9-yl)-7,8-difluorobenzo[4,5]imidazo[1,2-a]pyrimidine-3-carbonitrile (6f)

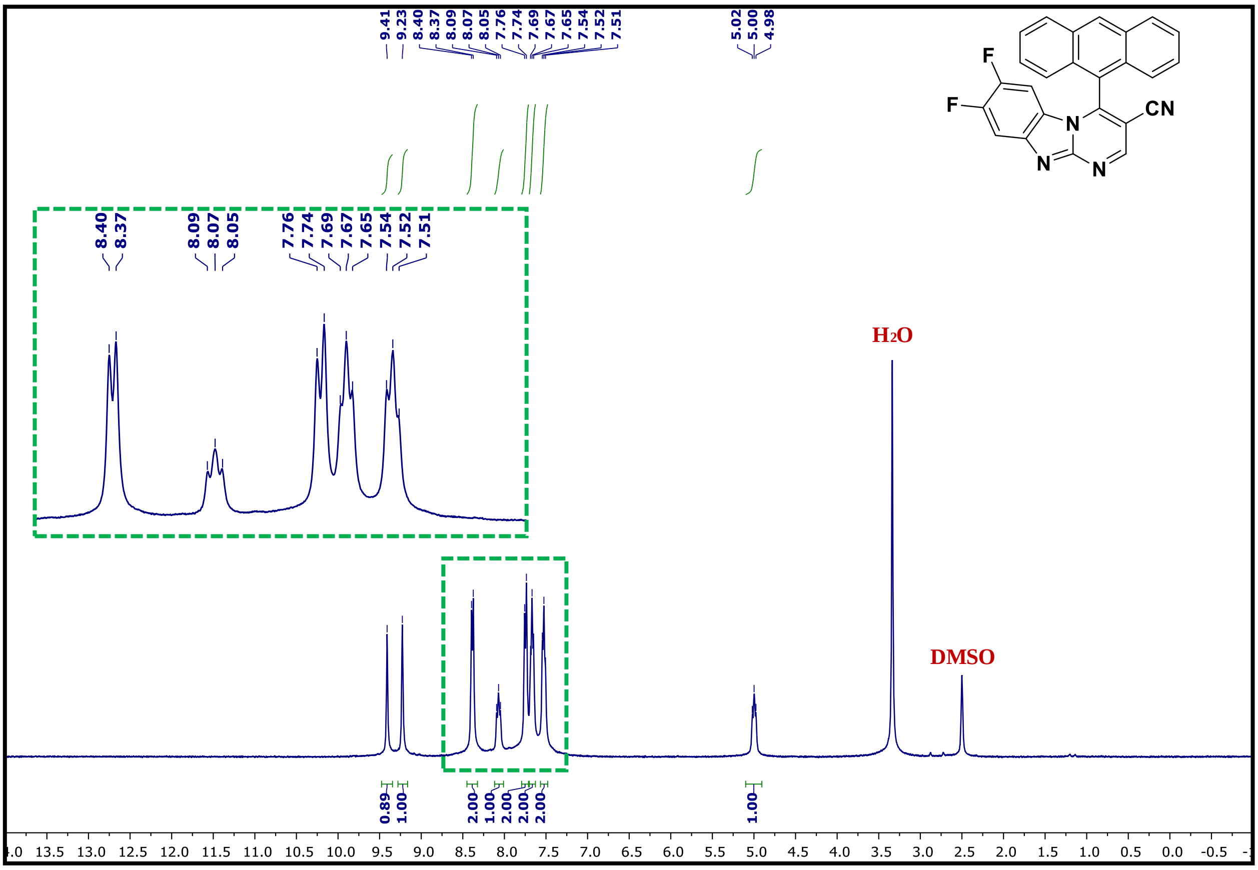

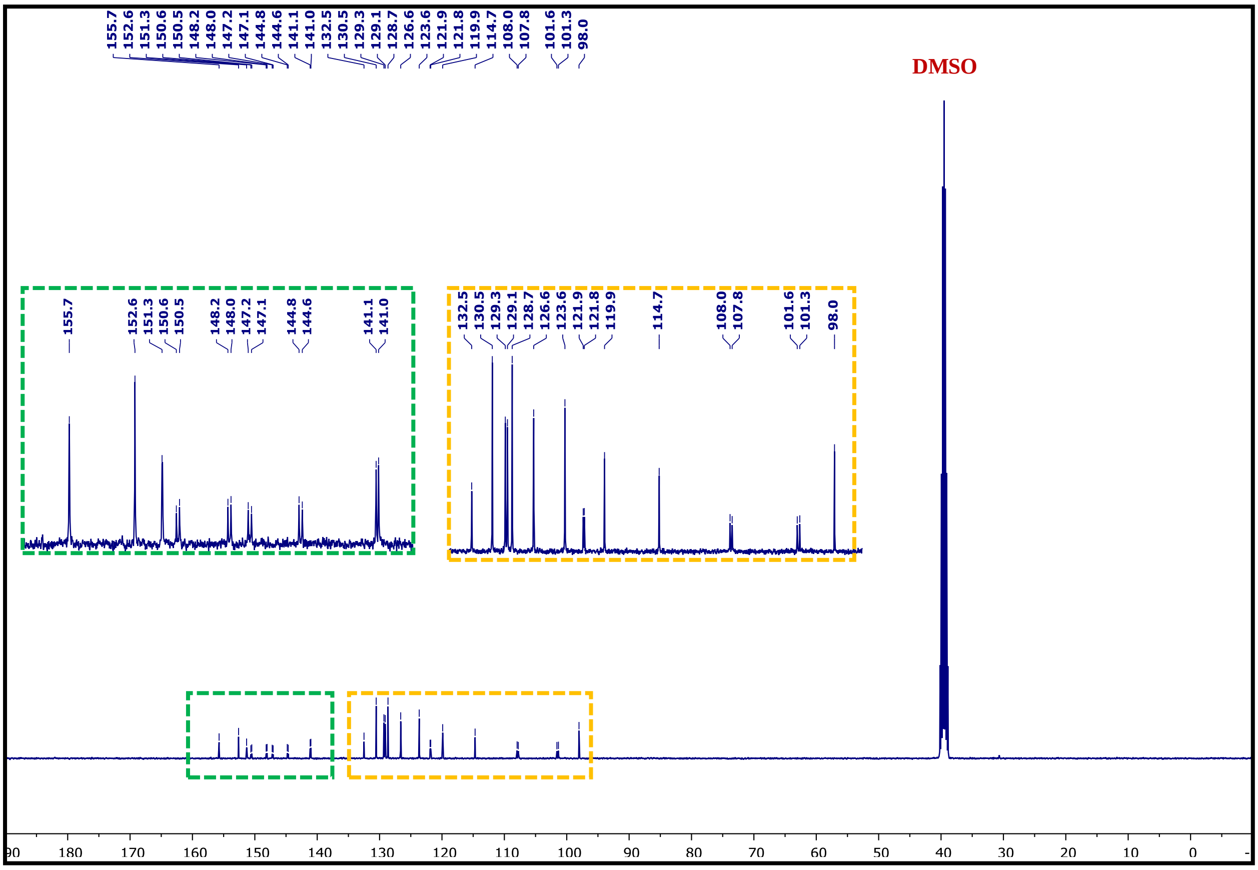

Figure S20. ^{1}H NMR (400 MHz, DMSO-d_6) and ^{13}C NMR (100 MHz, DMSO-d_6) spectra of **6f**

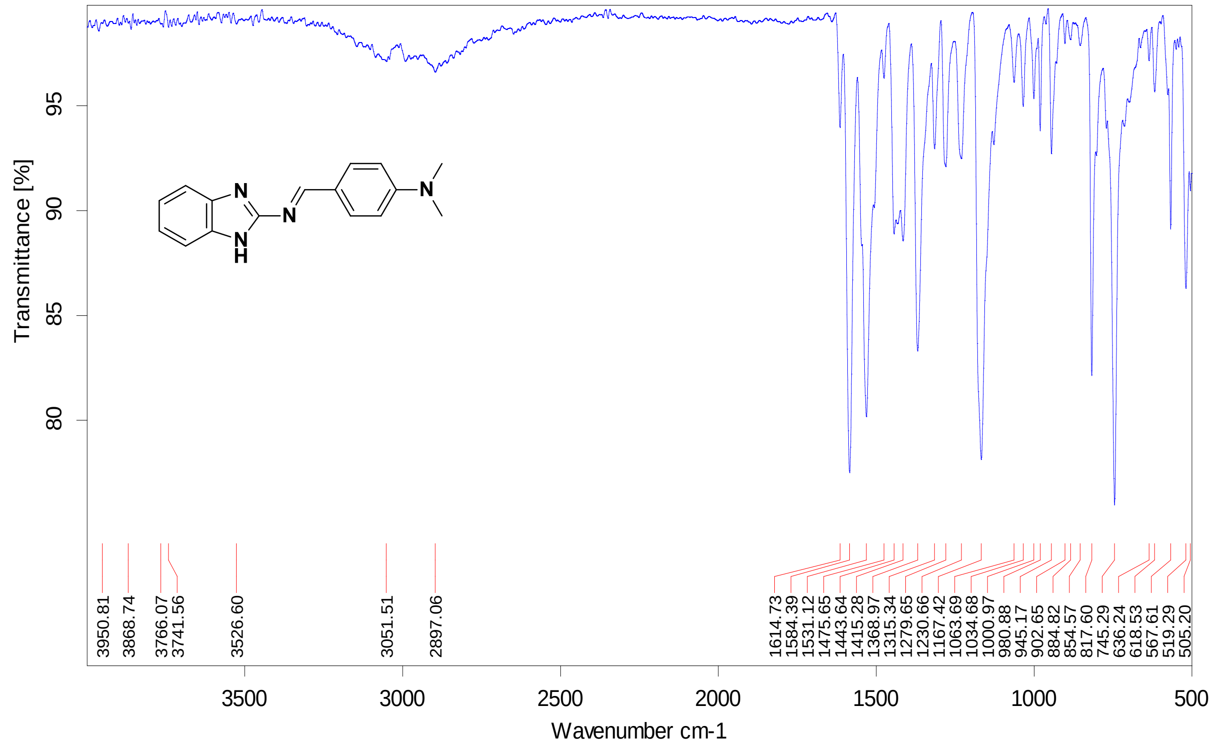

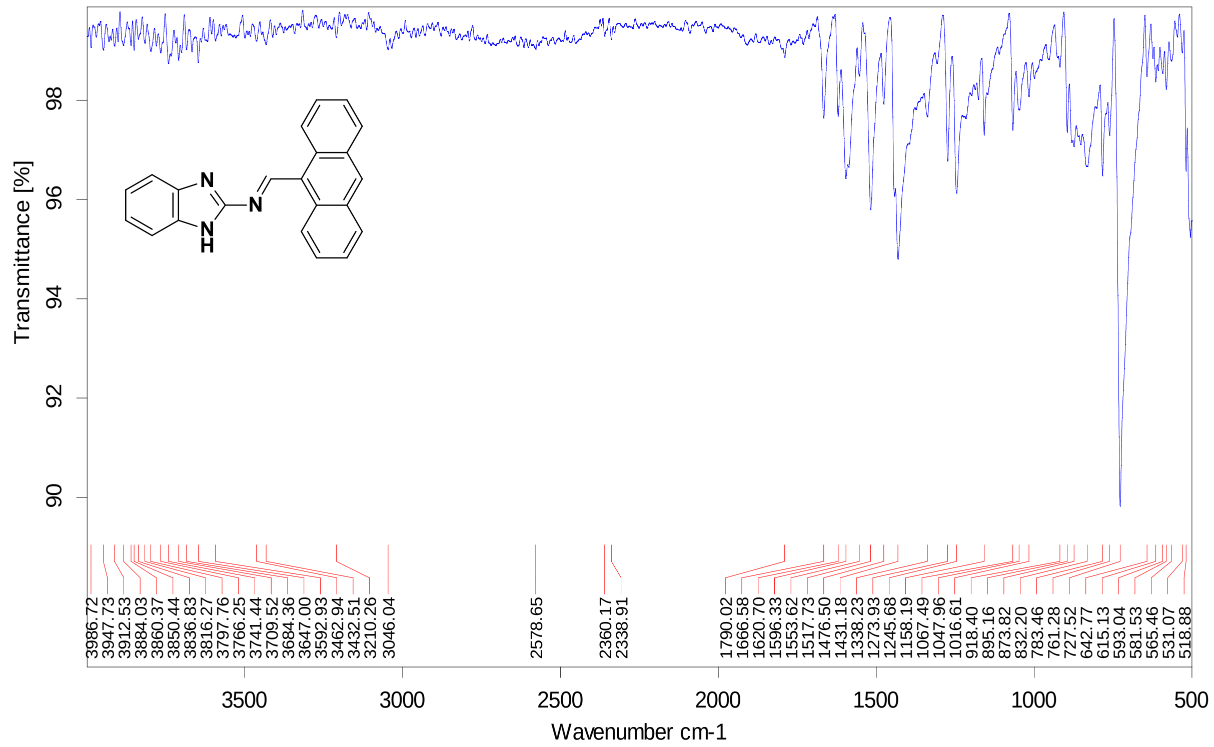

Figure S21. IR spectra of **3a** and **3c**

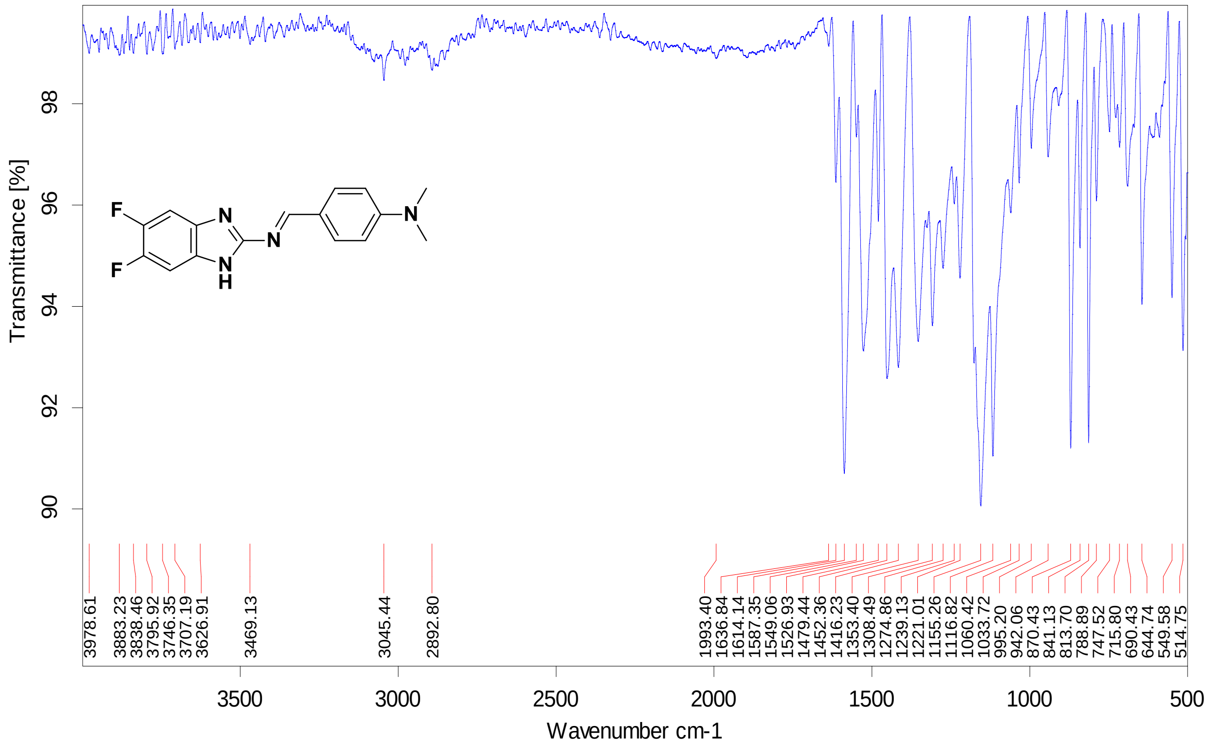

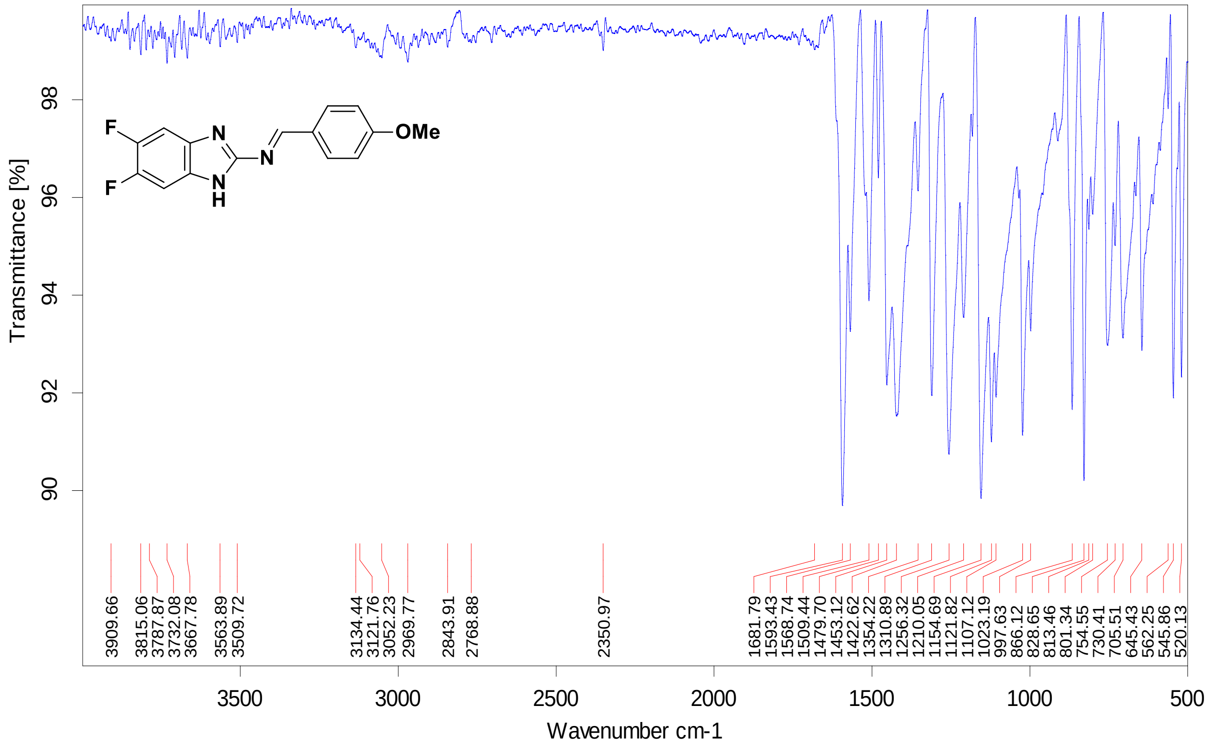

Figure S22. IR spectra of **3c** and **3d**

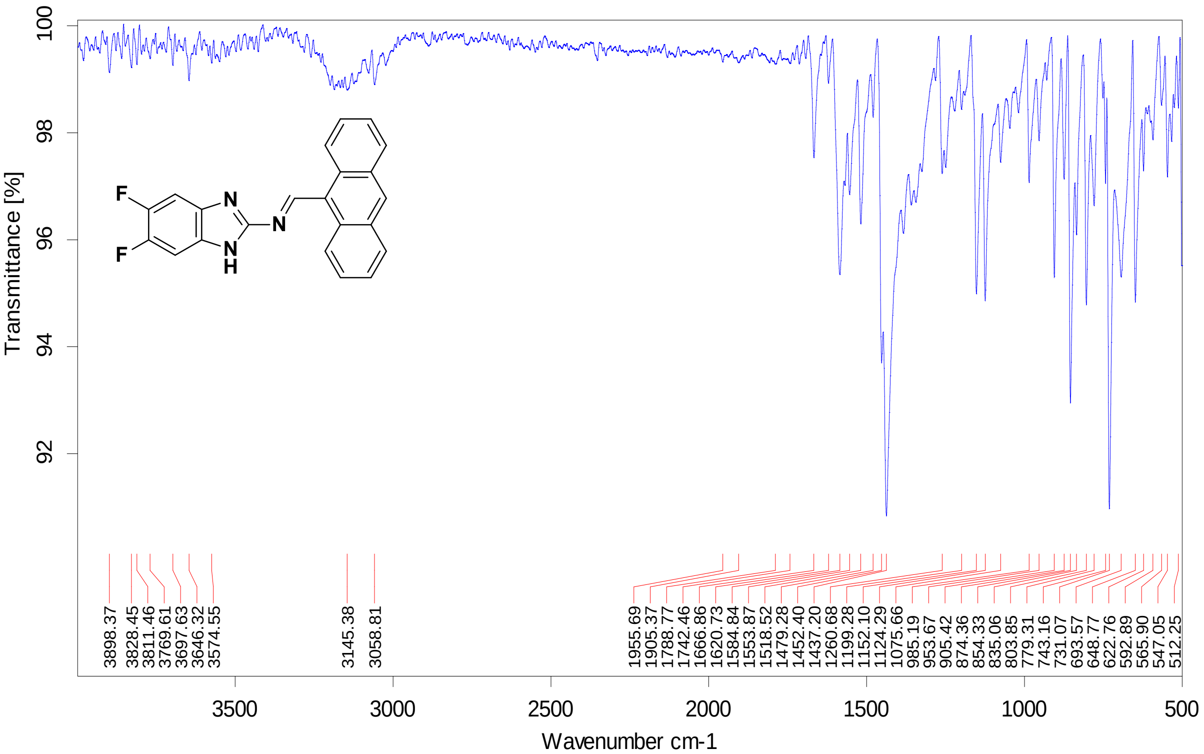

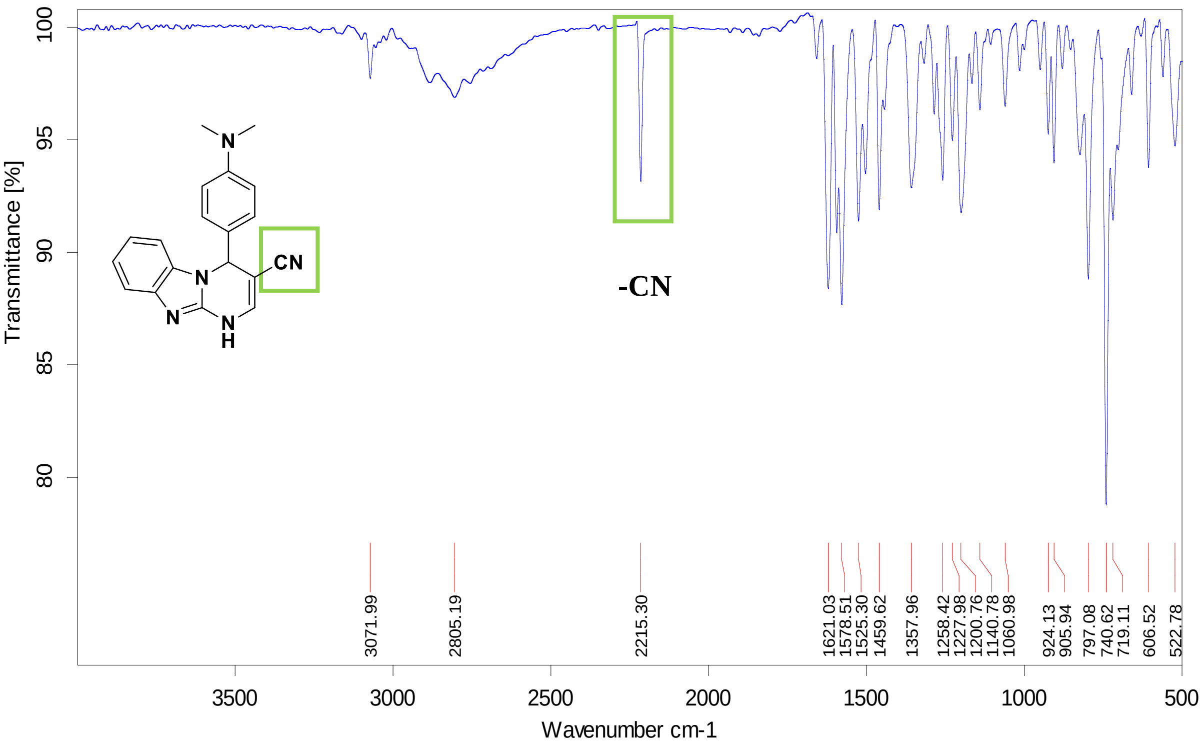

Figure S23. IR spectra of **3e** and **5a**

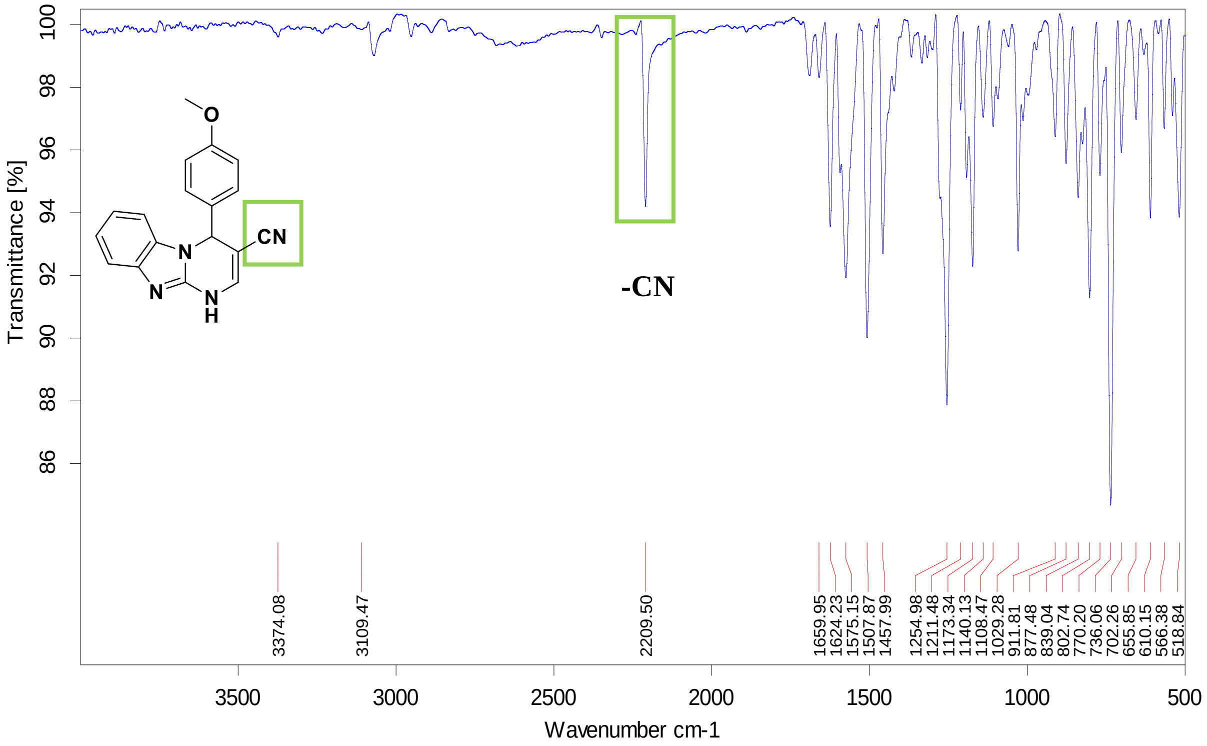

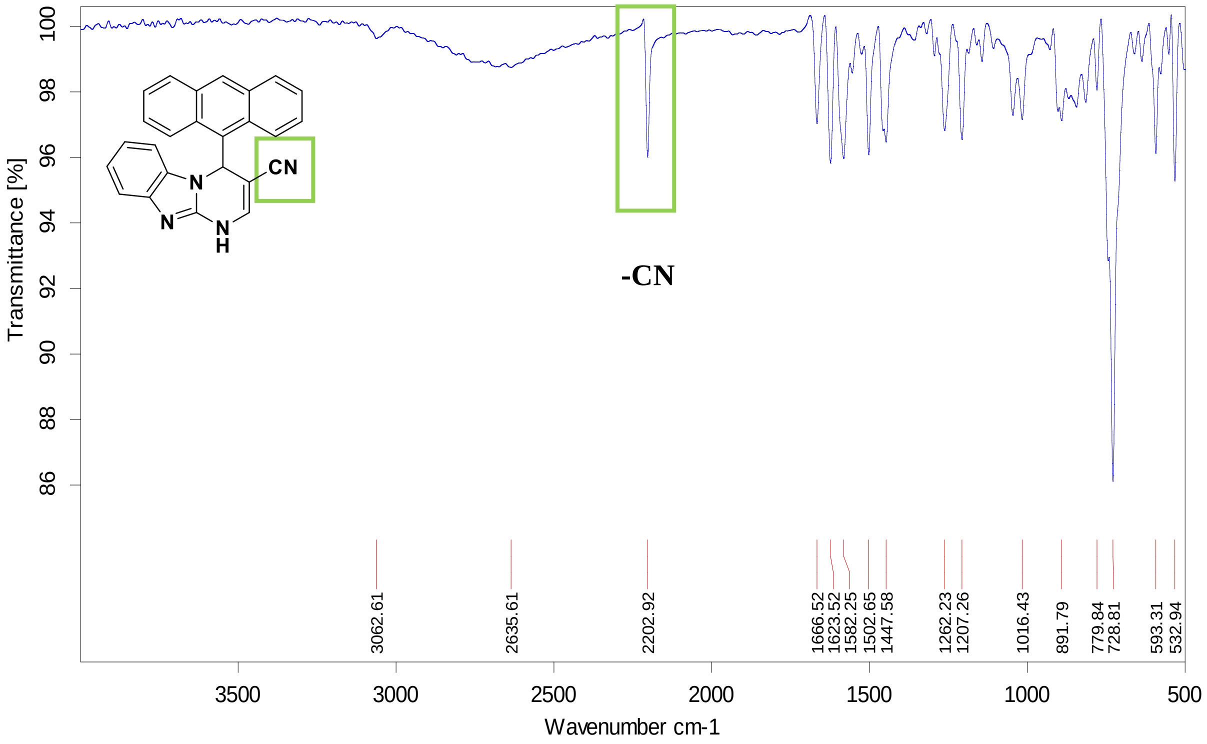

Figure S24. IR spectra of **5b** and **5c**

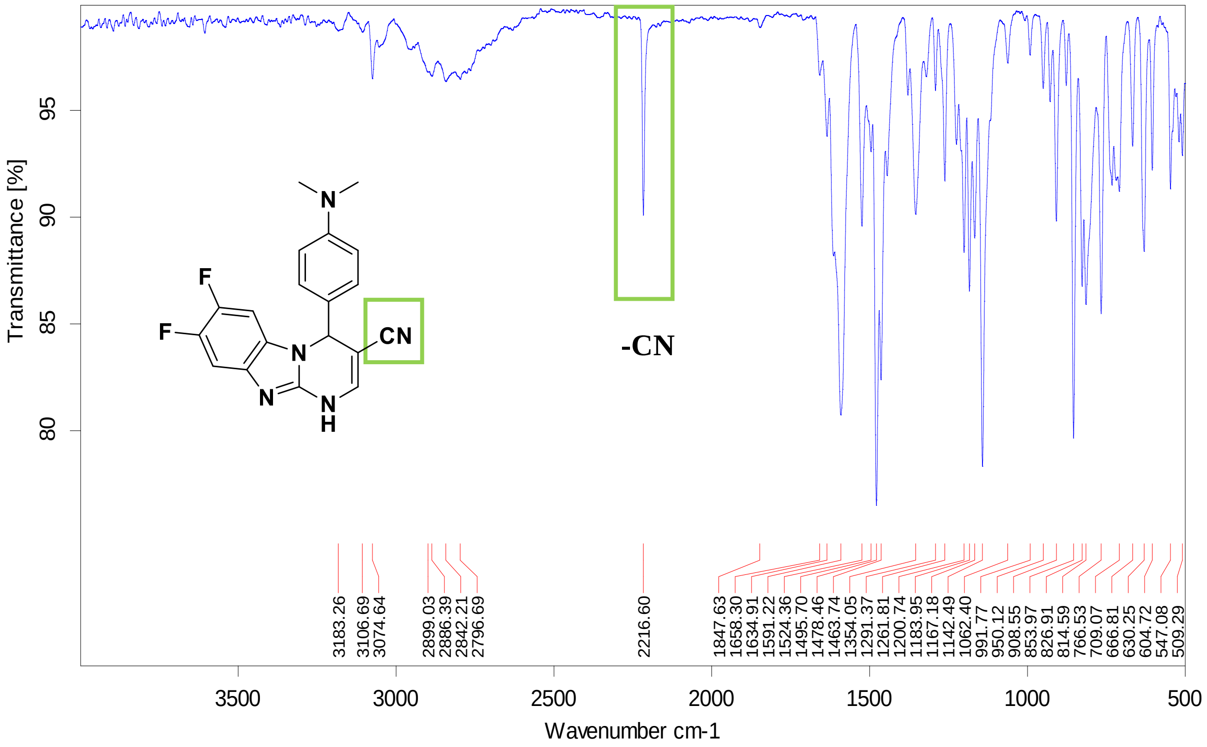

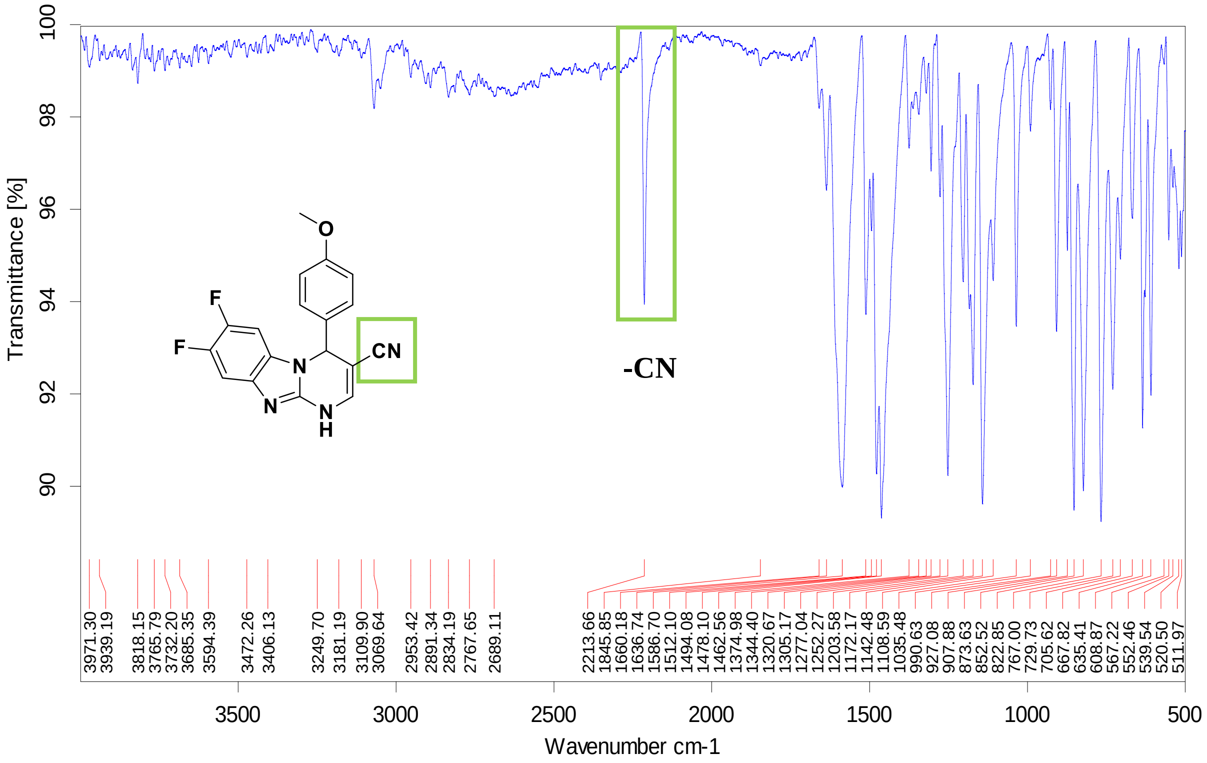

Figure S25. IR spectra of **5d** and **5e**

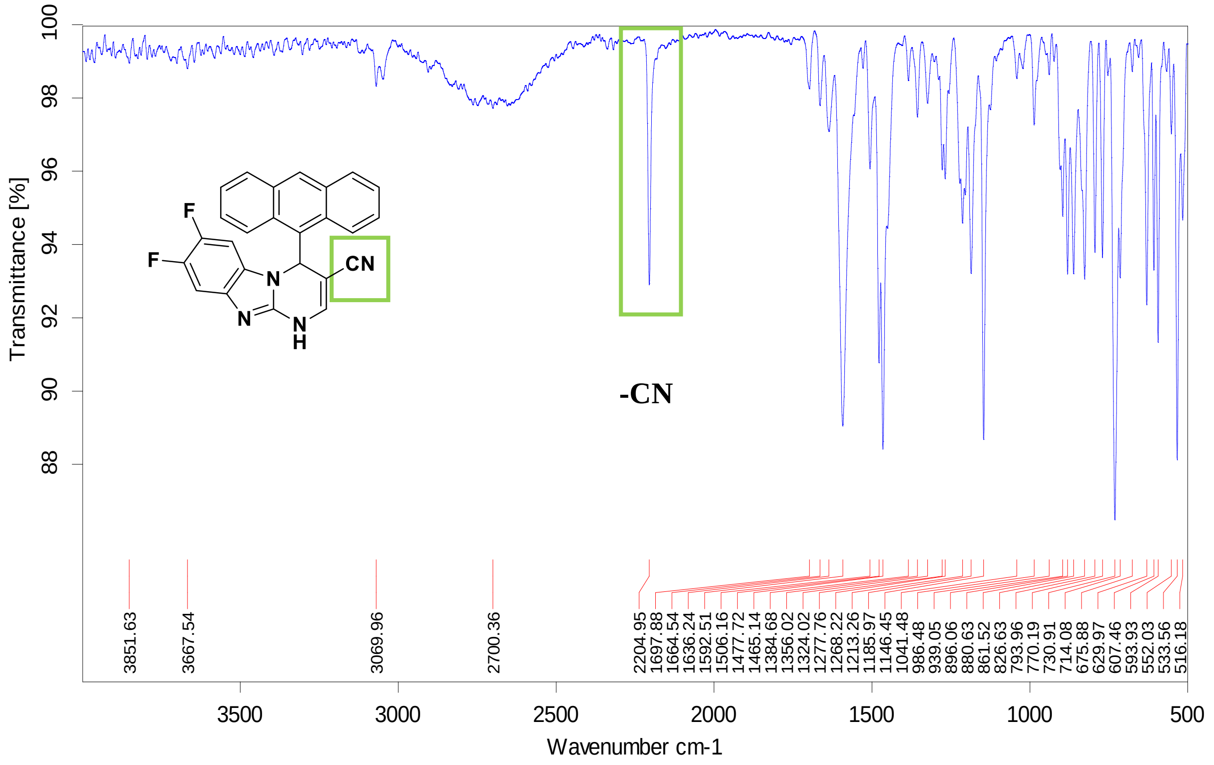

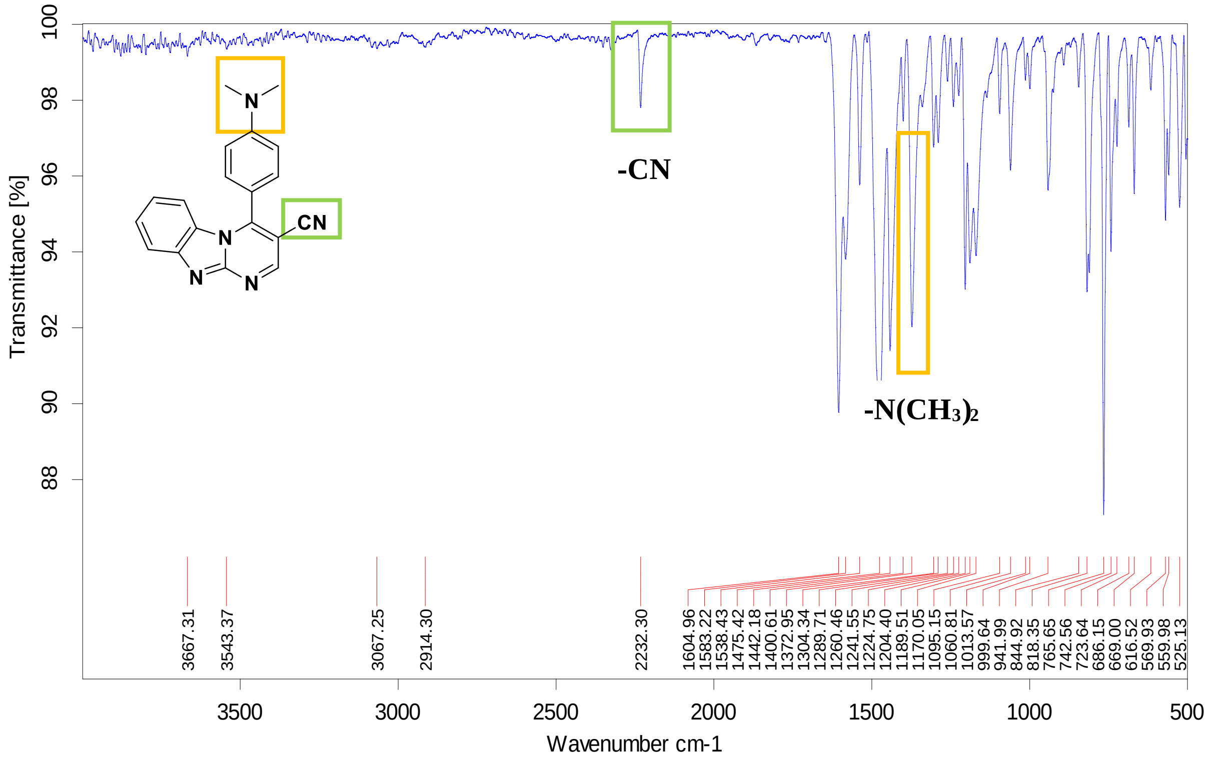

Figure S26. IR spectra of **5f** and **6a**

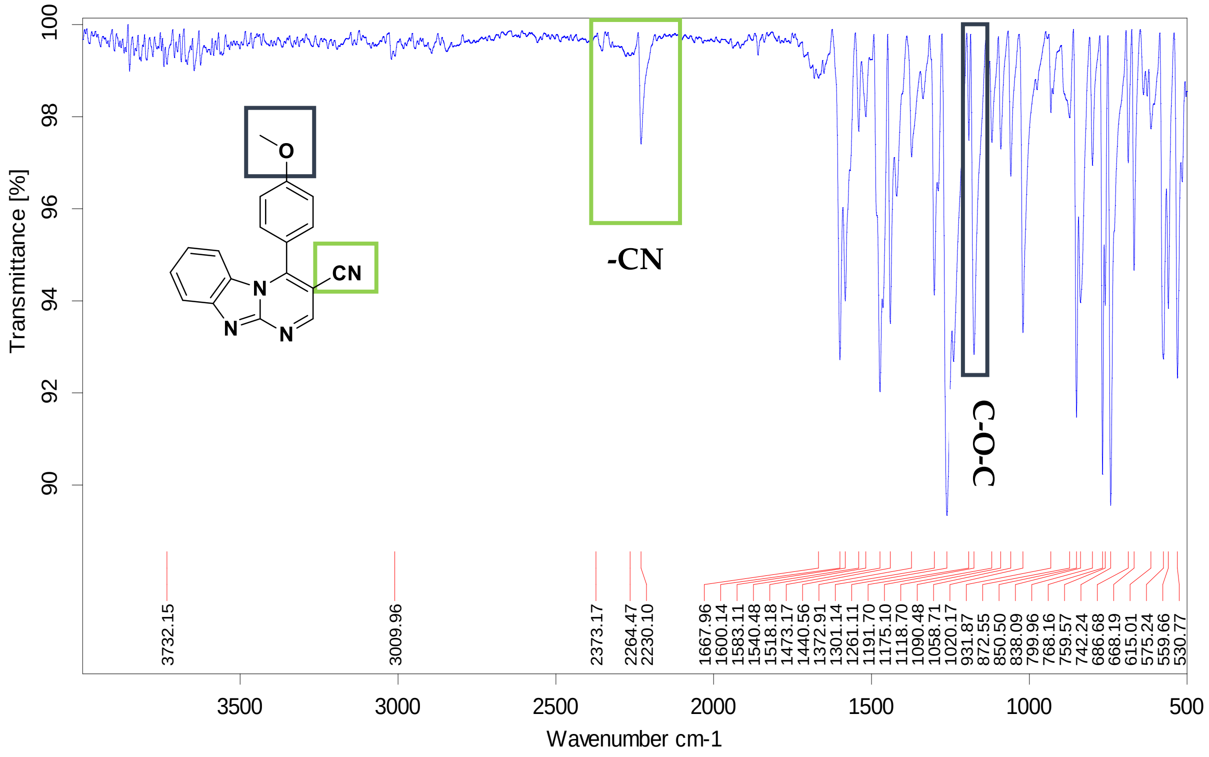

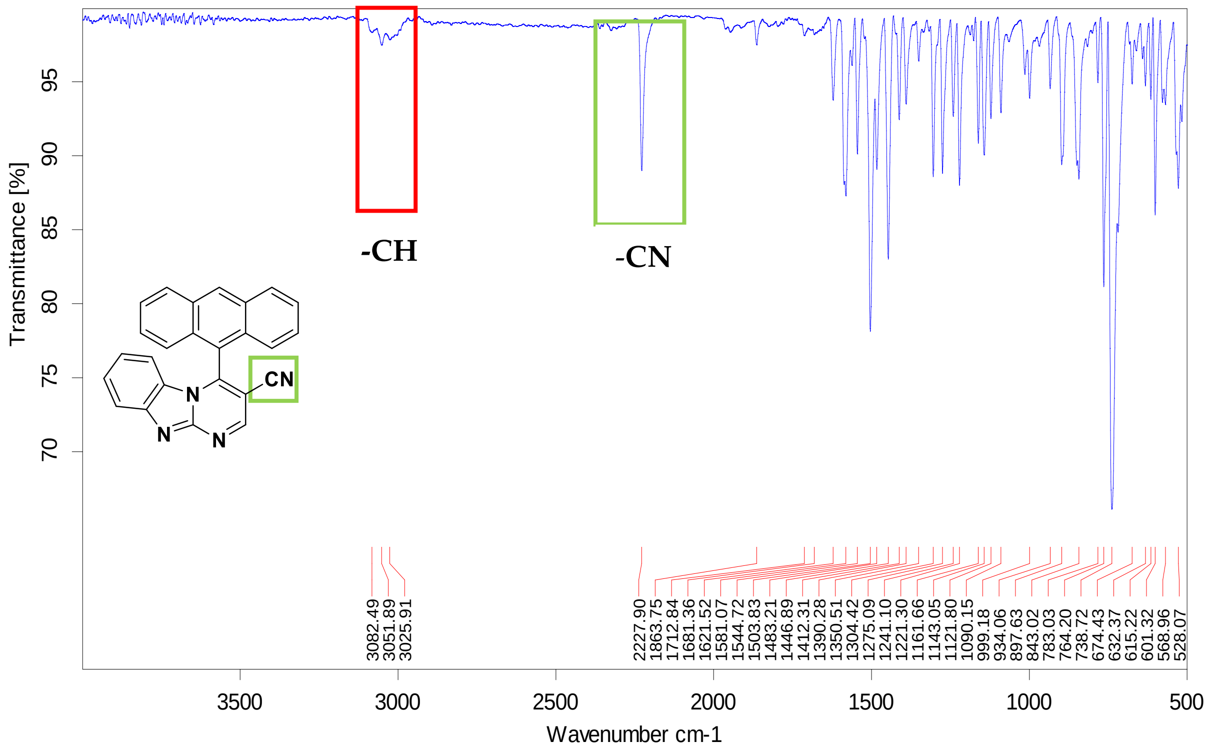

Figure S27. IR spectra of **6b** and **6c**

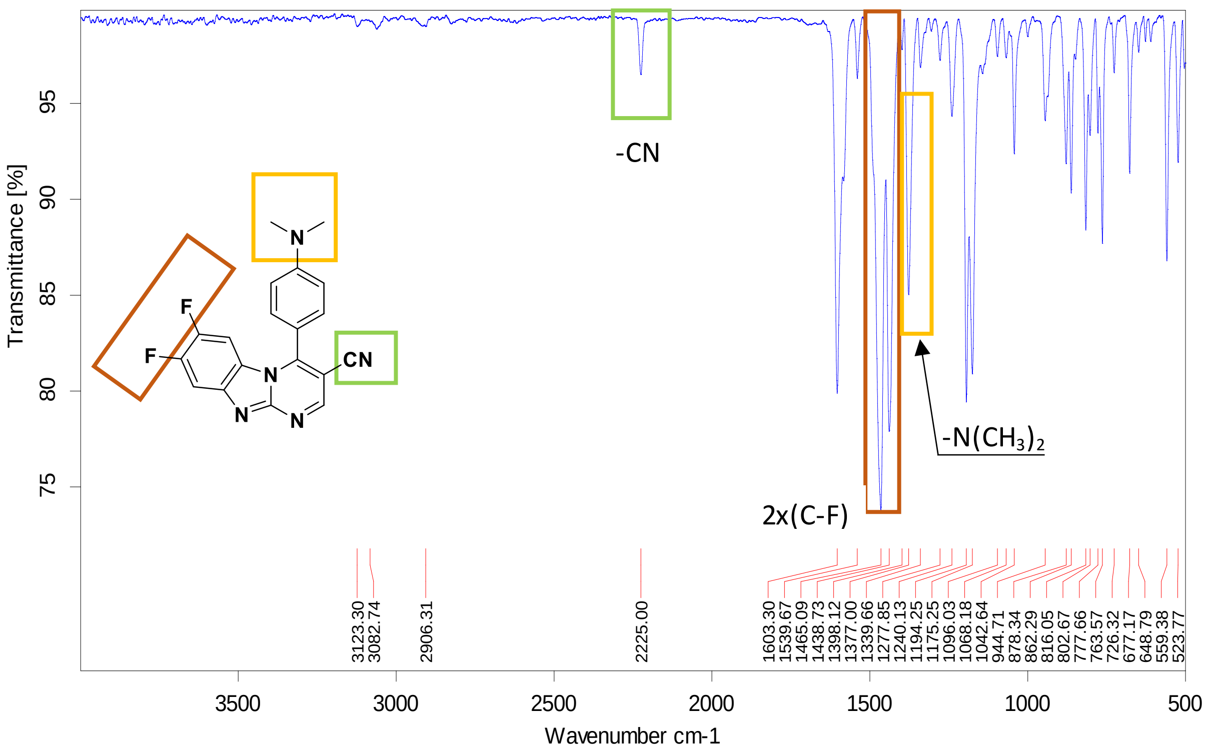

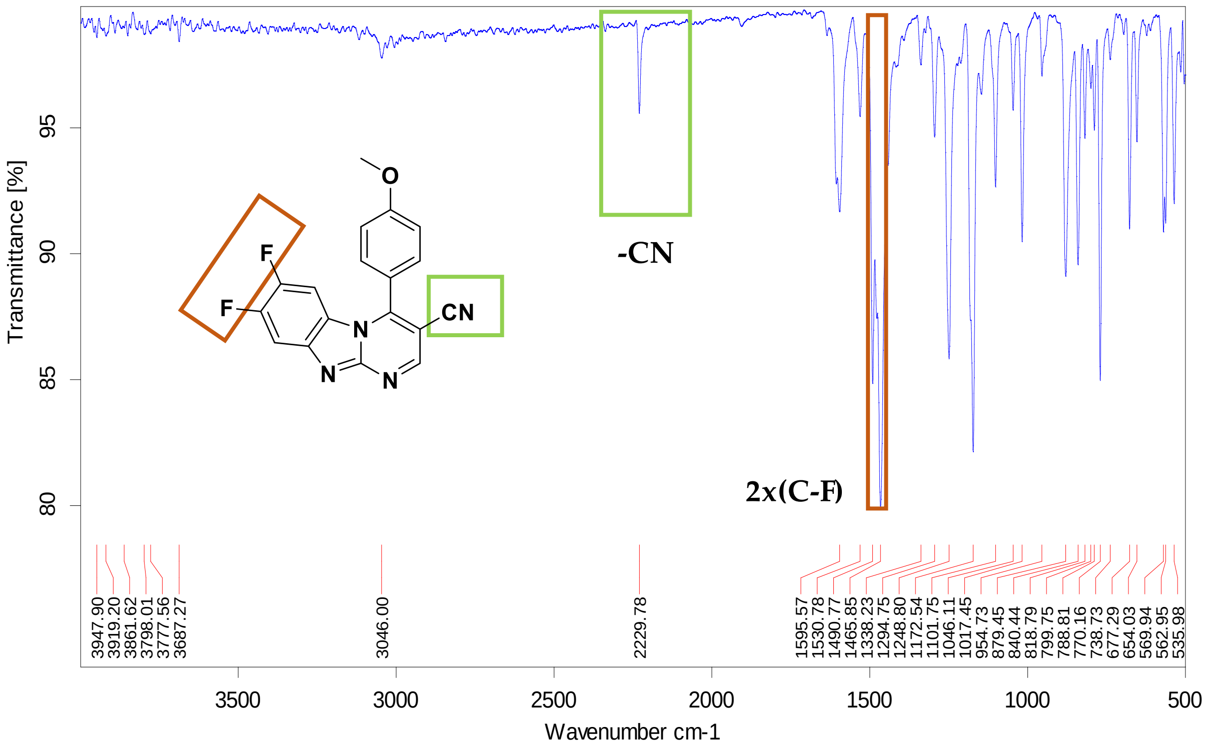

Figure S28. IR spectra of **6d** and **6e**

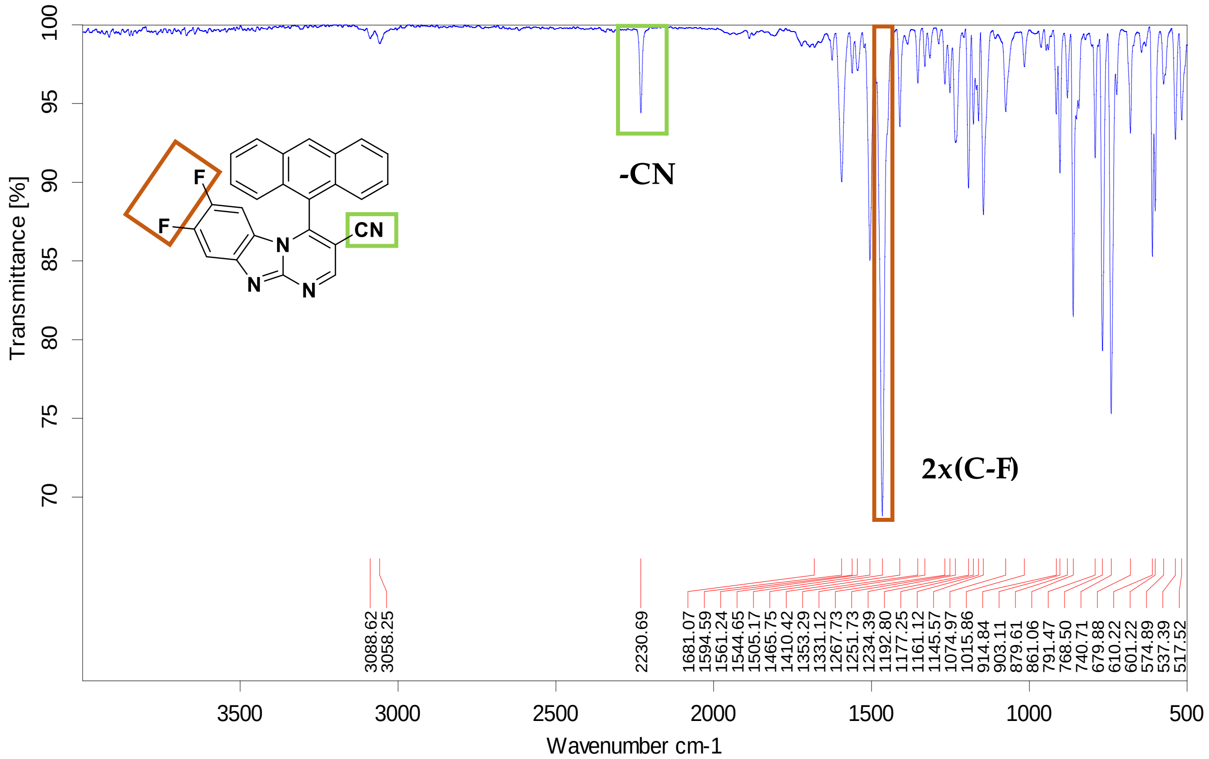

Figure S29. IR spectra of **6f**

7. Crystallography

Table S10. Crystal data and structure refinement for **6c**.

Identification code	exp_192
Empirical formula	$C_{51}H_{30}Cl_2N_8$
Formula weight	825.73
Temperature/K	295(2)
Crystal system	triclinic
Space group	P-1
a/Å	8.5135(4)
b/Å	10.4646(5)
c/Å	22.9053(12)
α/°	88.784(4)
β/°	85.741(4)
γ/°	82.301(4)
Volume/Å^3	2016.53(17)
Z	2
ρ_{calc}g/cm^3	1.360
μ/mm^{-1}	0.210
F(000)	852.0
Crystal size/mm^3	0.48 × 0.32 × 0.19
Radiation	MoKα (λ = 0.71073)
2Θ range for data collection/°	7.384 to 60.982
Index ranges	-10 ≤ h ≤ 11, -14 ≤ k ≤ 13, -31 ≤ l ≤ 29
Reflections collected	20634
Independent reflections	10876 [R_{int} = 0.0577, R_{sigma} = 0.0845]
Data/restraints/parameters	10876/14/571
Goodness-of-fit on F^2	1.003
Final R indexes [I>=2σ (I)]	R_1 = 0.0767, wR_2 = 0.1916
Final R indexes [all data]	R_1 = 0.1463, wR_2 = 0.2616
Largest diff. peak/hole / e Å^{-3}	0.34/-0.35

Table S11. Fractional Atomic Coordinates (×10^4) and Equivalent Isotropic Displacement Parameters (Å^2×10^3) for **6c**

U_{eq} is defined as 1/3 of of the trace of the orthogonalised U_{IJ} tensor.

Atom	*x*	*y*	*z*	U(eq)
Cl1	-68.3(17)	7778.6(15)	1999.3(6)	140.6(5)
Cl	2318(3)	8769(3)	1246.8(12)	205.3(14)
N11A	2874(2)	1028.5(18)	3662.2(9)	47.8(5)

Atom	*x*	*y*	*z*	U(eq)
N11	8740(2)	5344(2)	816.1(9)	51.7(5)
N1A	1501(3)	-758(2)	3423.8(11)	63.6(6)
N9A	4110(3)	-512(2)	3043.8(10)	62.9(6)
N1	11586(3)	5442(3)	761.5(11)	66.8(7)
C4A	1658(3)	1537(2)	4046.1(10)	44.1(5)
C25A	1194(3)	3926(2)	4069.4(11)	45.1(5)
N9	9771(3)	6858(2)	233.6(11)	67.3(7)
C26A	1795(3)	2763(2)	4338.7(11)	44.3(5)
C27A	2603(3)	2725(2)	4852.1(11)	47.9(6)
N2A	-1936(4)	1566(3)	4875.5(14)	84.9(8)
C24A	381(3)	3985(2)	3550.2(12)	52.3(6)
C1A	-906(3)	1288(2)	4532.5(13)	57.0(7)
C3A	381(3)	871(2)	4117.3(12)	50.7(6)
C25	6368(3)	4604(3)	1875.3(11)	49.6(6)
C26	7398(3)	3853(2)	1477.2(11)	48.1(6)
C12A	4357(3)	1383(2)	3501.7(11)	51.2(6)
C4	8839(3)	4363(3)	1219.3(11)	51.9(6)
C10A	2813(3)	-141(2)	3363.7(11)	53.5(6)
C2A	349(4)	-266(3)	3790.7(14)	61.2(7)
C20A	1466(3)	5112(2)	4323.0(12)	52.2(6)
C3	10341(3)	3936(3)	1390.0(12)	54.9(6)
C18	5823(3)	2078(3)	1634.5(11)	52.5(6)
C27	7159(3)	2583(2)	1345.8(11)	49.9(6)
N2	10819(3)	2185(3)	2188.0(14)	84.2(8)
C20	5025(3)	4093(3)	2149.8(11)	53.7(6)
C12	7461(3)	6050(3)	548.5(12)	54.9(6)
C18A	2859(3)	3921(3)	5103.5(12)	55.3(6)
C19A	2289(3)	5069(3)	4829.9(12)	58.4(7)
C14A	3224(3)	1561(3)	5129.7(12)	57.1(7)
C19	4802(3)	2841(3)	2019.6(12)	57.9(7)
C13A	5108(3)	406(3)	3127.5(12)	58.2(7)
C1	10576(3)	2958(3)	1827.3(14)	64.8(8)
C13	8137(4)	6961(3)	200.3(13)	61.3(7)
C8	5846(3)	5936(3)	563.4(13)	63.5(7)
C23A	-170(3)	5133(3)	3305.4(13)	62.7(7)
C8A	5122(3)	2431(3)	3642.3(13)	59.9(7)
C10	10120(3)	5895(3)	596.6(12)	58.1(7)
C21A	891(4)	6279(2)	4048.5(14)	66.8(8)
C2	11671(4)	4503(3)	1141.8(14)	65.3(8)

Atom	*x*	*y*	*z*	U(eq)
C14	8164(3)	1775(3)	942.8(13)	63.0(7)
C24	6591(3)	5885(3)	2030.1(13)	63.5(7)
C17A	3710(4)	3871(3)	5613.7(14)	70.6(8)
C5A	6646(4)	441(4)	2899.9(14)	74.1(9)
C22A	116(4)	6300(3)	3560.3(15)	70.7(8)
C21	3967(3)	4876(4)	2544.6(14)	72.9(9)
C7	4922(4)	6826(3)	232.3(14)	71.3(8)
C17	5606(4)	768(3)	1523.5(14)	68.6(8)
C5	7190(4)	7848(3)	-130.6(14)	74.0(8)
C6	5595(4)	7753(3)	-106.0(14)	76.6(9)
C15A	4025(4)	1560(3)	5612.5(15)	74.2(9)
C7A	6636(3)	2456(3)	3396.8(14)	72.7(9)
C6A	7400(4)	1458(4)	3037.6(14)	78.6(10)
C16	6628(4)	28(3)	1156.1(15)	76.8(9)
C15	7906(4)	546(3)	857.2(14)	73.6(9)
C23	5552(4)	6584(3)	2409.7(14)	76.4(9)
C16A	4273(4)	2743(4)	5863.8(15)	81.8(10)
C22	4222(4)	6095(4)	2667.6(15)	81.4(10)
C9	1343(8)	8823(7)	1907(3)	140(2)
Cl2	1823(19)	9395(15)	1352(9)	144(7)
C11	1820(30)	7950(30)	1720(20)	140(2)

Table S12. Anisotropic Displacement Parameters ($Å^2 \times 10^3$) for **6c**.

The Anisotropic displacement factor exponent takes the form: -$2\pi^2[h^2a^{*2}U_{11}+2hka^*b^*U_{12}+\ldots]$.

Atom	U_{11}	U_{22}	U_{33}	U_{23}	U_{13}	U_{12}
Cl1	137.1(10)	159.0(13)	136.1(11)	-9.9(9)	8.9(8)	-67.2(9)
Cl	180(2)	264(3)	189(2)	-97(2)	71.6(17)	-122(2)
N11A	57.6(12)	37.7(10)	47.8(13)	-1.2(8)	-2.3(9)	-5.9(9)
N11	51.4(12)	56.3(13)	49.7(13)	-2.4(10)	3.3(9)	-19.5(9)
N1A	88.3(17)	39.1(12)	64.7(16)	-7.4(10)	-3.3(12)	-13.3(11)
N9A	82.9(16)	47.5(13)	54.2(15)	-4.1(10)	0.0(12)	4.3(12)
N1	55.3(14)	75.5(17)	71.6(17)	-7.0(13)	9.9(11)	-23.2(12)
C4A	54.5(13)	32.1(11)	46.4(14)	5.4(9)	-7.6(10)	-7.1(10)
C25A	49.4(13)	34.8(11)	51.3(15)	-0.7(9)	0.5(10)	-9.4(9)
N9	72.2(16)	68.1(16)	64.6(16)	-1.1(12)	6.9(12)	-26.8(12)
C26A	49.8(13)	34.6(11)	49.6(14)	-1.2(9)	-2.6(10)	-9.8(9)
C27A	52.1(13)	42.7(13)	49.5(15)	-0.2(10)	-0.3(10)	-11.0(10)

Atom	U_{11}	U_{22}	U_{33}	U_{23}	U_{13}	U_{12}
N2A	89(2)	67.2(18)	101(2)	-18.9(15)	18.1(16)	-30.1(14)
C24A	60.7(15)	40.6(13)	56.7(17)	0.7(11)	-5.3(12)	-10.3(11)
C1A	61.9(16)	44.0(14)	67.8(19)	-4.8(12)	0.0(13)	-19.4(12)
C3A	59.2(15)	35.4(12)	58.8(16)	-1.6(10)	-5.8(11)	-10.3(10)
C25	48.2(13)	58.9(15)	41.8(14)	-1.8(11)	-1.9(10)	-7.5(11)
C26	43.1(12)	53.5(15)	49.1(15)	-5.8(11)	1.0(10)	-13.2(10)
C12A	56.4(14)	50.0(14)	45.2(15)	7.4(11)	-0.8(10)	-2.6(11)
C4	54.7(14)	53.6(15)	48.4(15)	-8.5(11)	1.9(11)	-12.7(11)
C10A	72.8(17)	36.1(12)	51.3(16)	-1.8(10)	-6.7(12)	-4.8(12)
C2A	74.3(19)	42.4(14)	71(2)	-3.7(12)	-12.7(15)	-20.2(13)
C20A	56.2(14)	39.7(13)	61.2(17)	-2.0(11)	1.1(11)	-11.5(10)
C3	49.6(14)	59.8(16)	55.9(17)	-5.2(12)	2.6(11)	-12.7(12)
C18	51.0(14)	63.4(16)	47.5(15)	8.3(12)	-11.1(11)	-20.6(12)
C27	48.6(13)	53.4(15)	49.7(15)	1.4(11)	-4.4(10)	-13.6(11)
N2	69.0(17)	96(2)	90(2)	22.4(17)	-10.9(14)	-20.5(15)
C20	43.2(13)	71.3(18)	44.7(15)	5.5(12)	-0.1(10)	-3.5(12)
C12	62.8(16)	56.9(15)	47.5(16)	-6.9(12)	-0.9(11)	-17.7(12)
C18A	60.1(15)	49.5(15)	58.2(17)	-7.0(12)	-2.8(12)	-13.7(12)
C19A	66.1(16)	46.5(14)	64.9(18)	-14.7(12)	0.5(13)	-16.1(12)
C14A	72.1(17)	46.2(14)	54.0(17)	4.8(11)	-10.4(13)	-9.2(12)
C19	47.2(14)	74.6(19)	53.4(17)	15.4(13)	-3.9(11)	-15.4(13)
C13A	67.8(17)	58.0(16)	43.9(16)	4.8(12)	-2.9(12)	7.8(13)
C1	48.7(15)	74(2)	74(2)	-2.0(16)	-1.4(13)	-16.3(13)
C13	70.7(18)	62.4(17)	53.3(17)	-4.4(13)	-0.1(13)	-20.0(14)
C8	60.7(16)	75(2)	57.8(18)	0.1(14)	-2.9(13)	-19.7(14)
C23A	71.6(18)	54.3(16)	63.1(19)	9.8(13)	-12.9(13)	-8.3(13)
C8A	59.7(16)	59.5(17)	61.8(18)	0.3(13)	-4.8(12)	-12.8(13)
C10	56.6(15)	68.2(18)	51.9(17)	-9.4(13)	11.2(11)	-23.8(13)
C21A	92(2)	34.3(13)	76(2)	0.2(13)	-5.3(16)	-13.4(13)
C2	52.0(16)	76(2)	70(2)	-11.5(15)	3.3(13)	-20.2(14)
C14	66.3(17)	63.3(18)	62.0(18)	-10.0(14)	2.5(13)	-20.2(14)
C24	66.2(17)	65.6(18)	59.0(18)	-11.1(13)	4.5(13)	-12.9(14)
C17A	78(2)	75(2)	62(2)	-18.3(16)	-12.0(15)	-16.3(16)
C5A	65.3(19)	87(2)	63(2)	5.2(16)	4.4(14)	10.6(17)
C22A	91(2)	39.0(14)	80(2)	8.1(13)	-4.9(17)	-4.5(14)
C21	56.1(16)	93(3)	65(2)	9.6(17)	6.8(13)	-2.1(16)
C7	67.1(18)	82(2)	67(2)	2.8(16)	-10.3(14)	-15.8(16)
C17	83(2)	70(2)	61.5(19)	8.9(15)	-9.9(15)	-37.4(16)
C5	91(2)	72(2)	62(2)	4.0(15)	-1.1(16)	-26.3(17)

Atom	U_{11}	U_{22}	U_{33}	U_{23}	U_{13}	U_{12}
C6	92(2)	77(2)	63(2)	4.7(16)	-12.1(16)	-14.6(18)
C15A	89(2)	67(2)	67(2)	7.7(15)	-19.0(16)	-5.2(16)
C7A	59.7(17)	90(2)	71(2)	4.2(17)	-3.5(15)	-20.1(16)
C6A	56.0(17)	109(3)	67(2)	15.8(19)	2.5(14)	-2.4(18)
C16	100(2)	57.9(18)	78(2)	0.6(16)	-16.3(19)	-25.7(17)
C15	80(2)	69(2)	74(2)	-17.4(16)	-3.8(16)	-13.5(16)
C23	83(2)	76(2)	67(2)	-18.8(16)	7.1(16)	-2.8(17)
C16A	93(2)	87(3)	67(2)	-4.7(18)	-26.7(17)	-5.9(19)
C22	77(2)	95(3)	67(2)	-14.3(18)	10.8(16)	2.4(19)
C9	160(5)	159(5)	111(4)	-29(4)	-8(3)	-56(4)
Cl2	115(9)	122(10)	202(15)	123(11)	-61(10)	-29(7)
C11	160(5)	159(5)	111(4)	-29(4)	-8(3)	-56(4)

Table S13. Bond Lengths for **6c**.

Atom	Atom	Length/Å	Atom	Atom	Length/Å
Cl1	C9	1.728(6)	C4	C3	1.379(4)
Cl1	C11	1.71(2)	C20A	C19A	1.397(4)
Cl	C9	1.668(6)	C20A	C21A	1.407(4)
N11A	C4A	1.365(3)	C3	C1	1.422(4)
N11A	C12A	1.385(3)	C3	C2	1.424(4)
N11A	C10A	1.423(3)	C18	C27	1.430(3)
N11	C4	1.364(3)	C18	C19	1.378(4)
N11	C12	1.404(3)	C18	C17	1.438(4)
N11	C10	1.431(3)	C27	C14	1.421(4)
N1A	C10A	1.359(3)	N2	C1	1.154(4)
N1A	C2A	1.302(4)	C20	C19	1.391(4)
N9A	C10A	1.298(3)	C20	C21	1.418(4)
N9A	C13A	1.390(4)	C12	C13	1.387(4)
N1	C10	1.352(4)	C12	C8	1.393(4)
N1	C2	1.298(4)	C18A	C19A	1.389(4)
C4A	C26A	1.482(3)	C18A	C17A	1.416(4)
C4A	C3A	1.366(3)	C14A	C15A	1.342(4)
C25A	C26A	1.404(3)	C13A	C5A	1.377(4)
C25A	C24A	1.416(3)	C13	C5	1.395(4)
C25A	C20A	1.435(3)	C8	C7	1.387(4)
N9	C13	1.389(4)	C23A	C22A	1.422(4)
N9	C10	1.310(4)	C8A	C7A	1.371(4)
C26A	C27A	1.404(3)	C21A	C22A	1.338(4)
C27A	C18A	1.438(3)	C14	C15	1.354(4)

Atom	Atom	Length/Å	Atom	Atom	Length/Å
C27A	C14A	1.418(3)	C24	C23	1.346(4)
N2A	C1A	1.144(3)	C17A	C16A	1.346(4)
C24A	C23A	1.355(3)	C5A	C6A	1.369(5)
C1A	C3A	1.425(4)	C21	C22	1.361(5)
C3A	C2A	1.423(4)	C7	C6	1.387(4)
C25	C26	1.394(3)	C17	C16	1.343(5)
C25	C20	1.425(3)	C5	C6	1.372(5)
C25	C24	1.434(4)	C15A	C16A	1.423(5)
C26	C4	1.480(3)	C7A	C6A	1.402(5)
C26	C27	1.414(3)	C16	C15	1.407(5)
C12A	C13A	1.403(4)	C23	C22	1.391(5)
C12A	C8A	1.403(4)	Cl2	C11	1.72(2)

Table S14 Bond Angles for **6c**.

Atom	Atom	Atom	Angle/˚	Atom	Atom	Atom	Angle/˚
C4A	N11A	C12A	132.6(2)	C19	C18	C17	121.8(2)
C4A	N11A	C10A	121.4(2)	C26	C27	C18	118.1(2)
C12A	N11A	C10A	105.8(2)	C26	C27	C14	124.0(2)
C4	N11	C12	133.1(2)	C14	C27	C18	117.9(2)
C4	N11	C10	121.2(2)	C19	C20	C25	118.5(2)
C12	N11	C10	105.6(2)	C19	C20	C21	122.4(3)
C2A	N1A	C10A	116.7(2)	C21	C20	C25	119.1(3)
C10A	N9A	C13A	105.3(2)	C13	C12	N11	104.8(2)
C2	N1	C10	116.5(2)	C13	C12	C8	122.3(3)
N11A	C4A	C26A	118.6(2)	C8	C12	N11	132.8(3)
N11A	C4A	C3A	116.2(2)	C19A	C18A	C27A	118.7(2)
C3A	C4A	C26A	125.2(2)	C19A	C18A	C17A	123.0(3)
C26A	C25A	C24A	123.3(2)	C17A	C18A	C27A	118.3(2)
C26A	C25A	C20A	118.2(2)	C18A	C19A	C20A	122.8(2)
C24A	C25A	C20A	118.5(2)	C15A	C14A	C27A	121.7(3)
C10	N9	C13	105.2(2)	C18	C19	C20	122.6(2)
C25A	C26A	C4A	118.4(2)	N9A	C13A	C12A	111.2(2)
C27A	C26A	C4A	119.0(2)	C5A	C13A	N9A	128.6(3)
C27A	C26A	C25A	122.4(2)	C5A	C13A	C12A	120.2(3)
C26A	C27A	C18A	118.7(2)	N2	C1	C3	177.3(3)
C26A	C27A	C14A	123.3(2)	N9	C13	C5	127.3(3)
C14A	C27A	C18A	117.9(2)	C12	C13	N9	112.3(3)
C23A	C24A	C25A	121.0(2)	C12	C13	C5	120.4(3)
N2A	C1A	C3A	176.9(3)	C7	C8	C12	116.6(3)

Atom	Atom	Atom	Angle/°	Atom	Atom	Atom	Angle/°
C4A	C3A	C1A	120.2(2)	C24A	C23A	C22A	119.8(3)
C4A	C3A	C2A	120.2(3)	C7A	C8A	C12A	116.9(3)
C2A	C3A	C1A	119.5(2)	N1	C10	N11	121.8(3)
C26	C25	C20	119.6(2)	N9	C10	N11	112.0(2)
C26	C25	C24	123.2(2)	N9	C10	N1	126.1(3)
C20	C25	C24	117.3(2)	C22A	C21A	C20A	121.6(3)
C25	C26	C4	119.2(2)	N1	C2	C3	124.5(3)
C25	C26	C27	121.5(2)	C15	C14	C27	120.9(3)
C27	C26	C4	119.1(2)	C23	C24	C25	121.2(3)
N11A	C12A	C13A	105.1(2)	C16A	C17A	C18A	121.7(3)
N11A	C12A	C8A	133.4(2)	C6A	C5A	C13A	118.5(3)
C8A	C12A	C13A	121.5(3)	C21A	C22A	C23A	120.7(3)
N11	C4	C26	121.3(2)	C22	C21	C20	121.1(3)
N11	C4	C3	115.8(2)	C6	C7	C8	121.0(3)
C3	C4	C26	122.8(2)	C16	C17	C18	121.1(3)
N1A	C10A	N11A	121.2(2)	C6	C5	C13	117.3(3)
N9A	C10A	N11A	112.7(2)	C5	C6	C7	122.4(3)
N9A	C10A	N1A	126.1(2)	C14A	C15A	C16A	120.5(3)
N1A	C2A	C3A	124.0(3)	C8A	C7A	C6A	121.5(3)
C19A	C20A	C25A	119.1(2)	C5A	C6A	C7A	121.4(3)
C19A	C20A	C21A	122.4(2)	C17	C16	C15	119.9(3)
C21A	C20A	C25A	118.4(2)	C14	C15	C16	121.4(3)
C4	C3	C1	120.7(2)	C24	C23	C22	121.4(3)
C4	C3	C2	120.1(3)	C17A	C16A	C15A	119.9(3)
C1	C3	C2	119.2(3)	C21	C22	C23	119.9(3)
C27	C18	C17	118.6(3)	Cl	C9	Cl1	114.3(3)
C19	C18	C27	119.6(2)	Cl1	C11	Cl2	110.1(15)

Table S15. Torsion Angles for **6c**.

A	B	C	D	Angle/°	A	B	C	D	Angle/°
N11A	C4A	C26A	C25A	90.8(3)	C10A	N11A	C12A	C8A	179.5(3)
N11A	C4A	C26A	C27A	-85.2(3)	C10A	N1A	C2A	C3A	1.1(4)
N11A	C4A	C3A	C1A	176.5(2)	C10A	N9A	C13A	C12A	-1.5(3)
N11A	C4A	C3A	C2A	-1.5(3)	C10A	N9A	C13A	C5A	177.9(3)
N11A	C12A	C13A	N9A	1.6(3)	C2A	N1A	C10A	N11A	-3.7(4)
N11A	C12A	C13A	C5A	-177.9(2)	C2A	N1A	C10A	N9A	176.4(3)
N11A	C12A	C8A	C7A	179.5(3)	C20A	C25A	C26A	C4A	-174.0(2)
N11	C4	C3	C1	177.1(2)	C20A	C25A	C26A	C27A	1.8(4)
N11	C4	C3	C2	-0.8(4)	C20A	C25A	C24A	C23A	-1.7(4)

A	B	C	D	Angle/°	A	B	C	D	Angle/°
N11	C12	C13	N9	0.3(3)	C20A	C21A	C22A	C23A	0.8(5)
N11	C12	C13	C5	-179.5(2)	C18	C27	C14	C15	-3.2(4)
N11	C12	C8	C7	-179.6(3)	C18	C17	C16	C15	-2.2(5)
N9A	C13A	C5A	C6A	179.2(3)	C27	C26	C4	N11	112.9(3)
C4A	N11A	C12A	C13A	174.7(2)	C27	C26	C4	C3	-70.7(3)
C4A	N11A	C12A	C8A	-4.8(5)	C27	C18	C19	C20	-1.0(4)
C4A	N11A	C10A	N1A	3.8(4)	C27	C18	C17	C16	-0.2(4)
C4A	N11A	C10A	N9A	-176.2(2)	C27	C14	C15	C16	0.9(5)
C4A	C26A	C27A	C18A	174.3(2)	C20	C25	C26	C4	-176.6(2)
C4A	C26A	C27A	C14A	-3.9(4)	C20	C25	C26	C27	-1.2(4)
C4A	C3A	C2A	N1A	1.6(4)	C20	C25	C24	C23	-1.4(4)
C25A	C26A	C27A	C18A	-1.5(4)	C20	C21	C22	C23	-0.9(5)
C25A	C26A	C27A	C14A	-179.7(2)	C12	N11	C4	C26	-1.6(4)
C25A	C24A	C23A	C22A	2.1(4)	C12	N11	C4	C3	-178.2(3)
C25A	C20A	C19A	C18A	-0.2(4)	C12	N11	C10	N1	-179.7(2)
C25A	C20A	C21A	C22A	-0.4(4)	C12	N11	C10	N9	0.7(3)
N9	C13	C5	C6	178.6(3)	C12	C13	C5	C6	-1.6(4)
C26A	C4A	C3A	C1A	-5.1(4)	C12	C8	C7	C6	1.6(5)
C26A	C4A	C3A	C2A	176.9(2)	C18A	C27A	C14A	C15A	0.1(4)
C26A	C25A	C24A	C23A	-179.7(2)	C18A	C17A	C16A	C15A	0.4(5)
C26A	C25A	C20A	C19A	-1.0(4)	C19A	C20A	C21A	C22A	179.4(3)
C26A	C25A	C20A	C21A	178.9(2)	C19A	C18A	C17A	C16A	-178.8(3)
C26A	C27A	C18A	C19A	0.3(4)	C14A	C27A	C18A	C19A	178.6(2)
C26A	C27A	C18A	C17A	-178.5(2)	C14A	C27A	C18A	C17A	-0.2(4)
C26A	C27A	C14A	C15A	178.3(3)	C14A	C15A	C16A	C17A	-0.5(6)
C27A	C18A	C19A	C20A	0.5(4)	C19	C18	C27	C26	1.1(4)
C27A	C18A	C17A	C16A	-0.1(5)	C19	C18	C27	C14	-178.7(2)
C27A	C14A	C15A	C16A	0.2(5)	C19	C18	C17	C16	-178.6(3)
C24A	C25A	C26A	C4A	3.9(4)	C19	C20	C21	C22	179.4(3)
C24A	C25A	C26A	C27A	179.8(2)	C13A	N9A	C10A	N11A	0.9(3)
C24A	C25A	C20A	C19A	-179.0(2)	C13A	N9A	C10A	N1A	-179.2(2)
C24A	C25A	C20A	C21A	0.9(4)	C13A	C12A	C8A	C7A	0.0(4)
C24A	C23A	C22A	C21A	-1.6(5)	C13A	C5A	C6A	C7A	-0.6(5)
C1A	C3A	C2A	N1A	-176.4(3)	C1	C3	C2	N1	-176.5(3)
C3A	C4A	C26A	C25A	-87.5(3)	C13	N9	C10	N11	-0.5(3)
C3A	C4A	C26A	C27A	96.5(3)	C13	N9	C10	N1	179.9(3)
C25	C26	C4	N11	-71.6(3)	C13	C12	C8	C7	-3.1(4)
C25	C26	C4	C3	104.9(3)	C13	C5	C6	C7	0.3(5)
C25	C26	C27	C18	-0.1(4)	C8	C12	C13	N9	-177.1(3)

A	B	C	D	Angle/°	A	B	C	D	Angle/°
C25	C26	C27	C14	179.7(2)	C8	C12	C13	C5	3.1(4)
C25	C20	C19	C18	-0.3(4)	C8	C7	C6	C5	-0.3(5)
C25	C20	C21	C22	-0.5(4)	C8A	C12A	C13A	N9A	-178.8(2)
C25	C24	C23	C22	0.0(5)	C8A	C12A	C13A	C5A	1.7(4)
C26	C25	C20	C19	1.3(4)	C8A	C7A	C6A	C5A	2.4(5)
C26	C25	C20	C21	-178.7(2)	C10	N11	C4	C26	175.7(2)
C26	C25	C24	C23	178.9(3)	C10	N11	C4	C3	-1.0(3)
C26	C4	C3	C1	0.5(4)	C10	N11	C12	C13	-0.5(3)
C26	C4	C3	C2	-177.4(2)	C10	N11	C12	C8	176.4(3)
C26	C27	C14	C15	177.0(3)	C10	N1	C2	C3	-0.1(4)
C12A	N11A	C4A	C26A	5.3(4)	C10	N9	C13	C12	0.1(3)
C12A	N11A	C4A	C3A	-176.2(2)	C10	N9	C13	C5	179.9(3)
C12A	N11A	C10A	N1A	-179.9(2)	C21A	C20A	C19A	C18A	179.9(3)
C12A	N11A	C10A	N9A	0.1(3)	C2	N1	C10	N11	-1.8(4)
C12A	C13A	C5A	C6A	-1.4(4)	C2	N1	C10	N9	177.8(3)
C12A	C8A	C7A	C6A	-2.0(4)	C24	C25	C26	C4	3.1(4)
C4	N11	C12	C13	177.0(3)	C24	C25	C26	C27	178.5(2)
C4	N11	C12	C8	-6.0(5)	C24	C25	C20	C19	-178.4(2)
C4	N11	C10	N1	2.4(4)	C24	C25	C20	C21	1.6(4)
C4	N11	C10	N9	-177.3(2)	C24	C23	C22	C21	1.1(5)
C4	C26	C27	C18	175.4(2)	C17A	C18A	C19A	C20A	179.3(3)
C4	C26	C27	C14	-4.8(4)	C21	C20	C19	C18	179.7(3)
C4	C3	C2	N1	1.4(5)	C17	C18	C27	C26	-177.4(2)
C10A	N11A	C4A	C26A	-179.6(2)	C17	C18	C27	C14	2.8(4)
C10A	N11A	C4A	C3A	-1.1(3)	C17	C18	C19	C20	177.5(2)
C10A	N11A	C12A	C13A	-1.0(3)	C17	C16	C15	C14	1.9(5)

Table S16. Hydrogen Atom Coordinates (Å×10^4) and Isotropic Displacement Parameters (Å^2×10^3) for **6c**.

Atom	*x*	*y*	*z*	U(eq)
H24A	221.1	3223.77	3374.61	63
H19A	2463.08	5842.14	4990.91	70
H14A	3071.82	778.22	4973	68
H19	3928.42	2504.2	2199.46	70
H8	5410.77	5296.55	783.99	76
H23A	-732.14	5157.16	2971.6	75
H8A	4624.82	3079.13	3890.31	72
H21A	1052.48	7054.45	4210.2	80
H14	9009.68	2095.6	734.83	76

Atom	*x*	*y*	*z*	U(eq)
H24	7468.34	6239.3	1865.14	76
H17A	3884.47	4638.28	5780.18	85
H5A	7159.41	-212.6	2658.27	89
H22A	-239.31	7085.42	3385.79	85
H21	3083.22	4549.95	2722.5	88
H7	3833.3	6799.33	237.31	86
H17	4742.61	428.01	1709.24	82
H5	7622.92	8478.71	-358.43	89
H6	4941.09	8332.27	-324.29	92
H15A	4421.64	781.77	5784.17	89
H7A	7167.44	3149.83	3470.16	87
H6A	8442.16	1488.06	2889.71	94
H16	6490.49	-826.6	1099.41	92
H15	8589.35	33.32	595.08	88
H23	5727.46	7412.35	2501.68	92
H16A	4822.35	2736.43	6200.12	98
H22	3509.67	6599.45	2923.37	98
H9A	824.85	9695.39	1974.83	168
H9B	2107.12	8621.78	2200.31	168
H11A	2203.57	7245.2	1453.81	168
H11B	2522.28	7906.73	2034.96	168
H2	12730(40)	4190(30)	1255(13)	72(9)
H2A	-530(30)	-670(30)	3811(12)	65(8)

Table S17. Atomic Occupancy for **6c**.

Atom	*Occupancy*	Atom	*Occupancy*	Atom	*Occupancy*
Cl	0.9	C9	0.9	H9A	0.9
H9B	0.9	Cl2	0.1	C11	0.1
H11A	0.1	H11B	0.1		

Crystal structure determination of 6c.

Crystal Data for $C_{51}H_{30}Cl_2N_8$ (*M* =825.73 g/mol): triclinic, space group P-1 (no. 2), a = 8.5135(4) Å, b = 10.4646(5) Å, c = 22.9053(12) Å, α = 88.784(4)°, β = 85.741(4)°, γ = 82.301(4)°, V = 2016.53(17) Å^3, Z = 2, T = 295(2) K, μ(MoKα) = 0.210 mm^{-1}, $Dcalc$ = 1.360 g/cm^3, 20634 reflections measured (7.384° ≤ 2Θ ≤ 60.982°), 10876 unique (R_{int} = 0.0577, R_{sigma} = 0.0845) which were used in all calculations. The final R_1 was 0.0767 (I > 2σ(I)) and wR_2 was 0.2616 (all data).

Refinement model description

Number of restraints - 14, number of constraints - unknown.

Details:

1. Fixed Uiso

At 1.2 times of:

All C(H) groups, All C(H,H) groups

2. Restrained distances

C11-Cl1

1.7 with sigma of 0.02

C11-Cl2

1.7 with sigma of 0.02

3. Uiso/Uaniso restraints and constraints

Uanis(Cl2) ≈ Ueq, Uanis(C11) ≈ Ueq: with sigma of 0.01 and sigma for terminal atoms of 0.02

Uanis(C11) = Uanis(C9)

4. Others

Fixed Sof: Cl(0.9) C9(0.9) H9A(0.9) H9B(0.9) Cl2(0.1) C11(0.1) H11A(0.1) H11B(0.1)

5.a Secondary CH2 refined with riding coordinates:

C9(H9A,H9B), C11(H11A,H11B)

5.b Aromatic/amide H refined with riding coordinates:

C24A(H24A), C19A(H19A), C14A(H14A), C19(H19), C8(H8), C23A(H23A), C8A(H8A), C21A(H21A), C14(H14), C24(H24), C17A(H17A), C5A(H5A), C22A(H22A), C21(H21), C7(H7), C17(H17), C5(H5), C6(H6), C15A(H15A), C7A(H7A), C6A(H6A), C16(H16), C15(H15), C23(H23), C16A(H16A), C22(H22)
